# Patterns of abnormal activations in severe mental disorders a transdiagnostic data-driven meta-analysis of task-based fMRI studies

**DOI:** 10.1017/S003329172400165X

**Published:** 2024-10

**Authors:** Mélanie Boisvert, Jules R. Dugré, Stéphane Potvin

**Affiliations:** 1 Research Center of the Institut Universitaire en Santé Mentale de Montréal, Montreal, Canada; 2 Department of Psychiatry and Addictology, Faculty of medicine, University of Montreal, Montreal, Canada; 3 School of Psychology and Centre for Human Brain Health, University of Birmingham, Birmingham, UK

**Keywords:** ALE, bipolar disorder, clustering analysis, fMRI, major depressive disorder, Schizophrenia, task, transdiagnostic

## Abstract

**Background:**

Studies suggest severe mental disorders (SMDs), such as schizophrenia, major depressive disorder and bipolar disorder, are associated with common alterations in brain activity, albeit with a graded level of impairment. However, discrepancies between study findings likely to results from both small sample sizes and the use of different functional magnetic resonance imaging (fMRI) tasks. To address these issues, data-driven meta-analytic approach designed to identify homogeneous brain co-activity patterns across tasks was conducted to better characterize the common and distinct alterations between these disorders.

**Methods:**

A hierarchical clustering analysis was conducted to identify groups of studies reporting similar neuroimaging results, independent of task type and psychiatric diagnosis. A traditional meta-analysis (activation likelihood estimation) was then performed within each of these groups of studies to extract their aberrant activation maps.

**Results:**

A total of 762 fMRI study contrasts were targeted, comprising 13 991 patients with SMDs. Hierarchical clustering analysis identified 5 groups of studies (meta-analytic groupings; MAGs) being characterized by distinct aberrant activation patterns across SMDs: (1) emotion processing; (2) cognitive processing; (3) motor processes, (4) reward processing, and (5) visual processing. While MAG1 was mostly commonly impaired, MAG2 was more impaired in schizophrenia, while MAG3 and MAG5 revealed no differences between disorder. MAG4 showed the strongest between-diagnoses differences, particularly in the striatum, posterior cingulate cortex, and ventromedial prefrontal cortex.

**Conclusions:**

SMDs are characterized mostly by common deficits in brain networks, although differences between disorders are also present. This study highlights the importance of studying SMDs simultaneously rather than independently.

## Introduction

Schizophrenia spectrum disorder (SCZ), bipolar disorder (BD), and major depressive disorder (MDD) are major causes of disability and premature death and are associated with impaired activities of daily living, and thus are referred to as severe mental disorders (SMDs) (National Institute of Mental Health, 1987). Although these three disorders are defined as distinct entities in clinical practice, SMDs share neurobiological alterations (McTeague et al., 2017, 2020) and significant levels of comorbid symptoms (Baethge et al., 2005). This has led a growing number of investigators to adopt a transdiagnostic approach to study the neurobiological bases of SMDs. This approach promises to help identify the neurobiological impairments shared by SMDs, and those that differ between them.

A growing body of research shows that SMDs share transdiagnostic genetic variants (The Brainstorm Consortium et al., 2018), risk factors (e.g. childhood adversity) (Xie et al., 2018) as well as overlap in pharmacological treatment. For example, atypical antipsychotics are primarily used for treating SCZ, but they can be used as main treatment for BD (Keramatian, Chakrabarty, Saraf, & Yatham, 2021) and as adjunctive treatment at lower doses for MDD (Mulder et al., 2018). SMDs share comorbidities, namely anxiety and depressive symptoms. Comorbidity rates for any anxiety disorder are estimated as high as 38%, 41%, and 75% for SCZ, BD, and MDD, respectively (Achim et al., 2011; Lamers et al., 2011; Yapici Eser, Kacar, Kilciksiz, Yalçinay-Inan, & Ongur, 2018). The majority of SCZ patients also experience mild to severe depressive symptoms (Herniman et al., 2019). It is also well established that BD patients will experience depressive symptoms a significant part of their life (Judd et al., 2003). Therefore, this shared symptomatology could possibly be explained by transdiagnostic neurobiological alterations.

Despite similarities across SMDs, differences can be noted between these disorders. For instance, there is reliable evidence of increased dopamine release in the associative striatum in psychosis, which is one of the main pillars of the aberrant salience hypothesis of psychosis (McCutcheon, Abi-Dargham, and Howes, 2019); by comparison, studies on dopamine alterations in MDD have produced mixed results (Belujon & Grace, 2017). Despite the high rates of polypharmacy in SMDs, there are differences in the pharmacological management of SMDs. For instance, antidepressants are not known to produce antipsychotic effects, and some antidepressants can increase the risk of experiencing a (hypo)manic episode (Bond, Noronha, Kauer-Sant'Anna, Lam, & Yatham, 2008). While mania, delusions and hallucinations can be noted in the three disorders, their prevalence, persistence, and severity differ from one to another. More specifically, the prevalence and severity of manic symptoms can be depicted as BD > SCZ > MDD (Angst et al., 2018; Malhi, Green, Fagiolini, Peselow, & Kumari, 2008), while the prevalence and persistence of delusions and hallucinations can be portrayed as SCZ > BD > MDD (Baethge et al., 2005). Finally, SMDs are all associated with global cognitive and theory of mind deficits, but significant variations in severity are observed between them (SCZ > BD > MDD) (Sheffield, Karcher, & Barch, 2018; Van Neerven, Bos, & Van Haren, 2021).

Structural neuroimaging studies highlighted abnormalities in the same regions across SMDs; however, effects were generally larger in SCZ compared to MDD (Cheon et al., 2022). In a cross-disorder review of ENIGMA findings, analyses on subcortical volumes, cortical thickness, cortical surface area, and diffusion tensor imaging metrics showed deficits in similar brain regions and pathways across SMDs. However, these abnormalities were scaled in severity across SMDs with SCZ showing moderate effects, BD intermediate effects and MDD minimal effects (Cheon et al., 2022). In regard to task-based functional neuroimaging studies, McTeague et al. (McTeague et al. 2017) conducted a meta-analysis of cognitive control tasks in SMDs and other psychiatric disorders, which highlighted common hypoactivation of the dorsal anterior cingulate cortex and common hyperactivation in the (right) ventrolateral prefrontal cortex (vlPFC). Hypoactivation was also observed in the (left) dorsolateral prefrontal cortex (dlPFC), but the effect was significantly larger in SCZ than other disorders. McTeague et al. (McTeague et al. 2020) conducted another meta-analysis with the same diagnosis groups but focusing on emotion processing tasks. Their results showed common aberrant activations in the amygdala, the (para-)hippocampus, the thalamus, the medial prefrontal cortex, the vlPFC, and the fusiform gyrus. While all three disorders contributed similarly to the medial prefrontal cortex cluster, differences were observed between SMDs in the case of the thalamus and the vlPFC. Finally, a transdiagnostic meta-analysis of 226 neuroimaging studies focusing on mood and anxiety disorders found common abnormal activation of limbic regions during affective and social processing experiments in mood disorders (Janiri et al., 2020).

Classical meta-analytic approaches consist of conducting meta-analyses on similar task-contrasts, as previously done (Janiri et al., 2020; McTeague et al., 2017, 2020). One key limitation of such study is that researchers manually classify task-contrasts and assume that these expert-driven categories rely on distinct brain networks, which is not always supported by the literature (Chen, Chaudhary, & Li, 2022; Lindquist, Wager, Kober, Bliss-Moreau, & Barrett, 2012). More importantly, this manual annotation can lead to jingle-jangle fallacy, preventing us to delineate the shared/distinct neural correlates in SMDs. Others rather perform task-independent analyses (i.e. across tasks) to identify whole-brain alterations (Schumer, Chase, Rozovsky, Eickhoff, & Phillips, 2023). However, this approach does not allow to investigate the heterogeneity of aberrant co-activation networks across studies, which may be of relevance to better clarify brain-behavior relationships in SMDs. In order to overcome these methodological limits, investigators performed cluster analyses to identify groups of experiments sharing similar aberrant co-activation patterns, regardless of the task in non-clinical participants (Bottenhorn et al., 2019; Dugré & Potvin, 2023; Laird et al., 2015) and in youth with psychiatric disorders (Dugré, Eickhoff, & Potvin, 2022). To the best of our knowledge, no transdiagnostic meta-analysis on SMDs has adopted this latter approach.

Using a data-driven meta-analytic approach designed to identify brain activity networks, we aimed to study brain activity alterations across and between SMDs, regardless of the experimental context. We focused on BD, MDD, and SCZ since these SMDs seem to present both shared and distinct neural alterations (Gong et al., 2020; Qi et al., 2022; Wang et al., 2021). Moreover, these SMDs have been widely studied, meaning that a sufficiently large number of studies will be available to run the planned clustering analyses. We hypothesized that at least three neural networks will be identified, involved in emotion, cognitive and reward processing. Coherently with the past literature, we expected to observe common effects across SMDs, especially in limbic regions (amygdala, hippocampus) (Sprooten et al., 2017), but specific alterations in some regions (e.g. thalamus in SCZ, ventrolateral prefrontal cortex in BD) (McTeague et al., 2020). Moreover, we expected a graded level of impairment in the dlPFC, which plays a key role in executive functions, with common alterations in the dorsal anterior cingulate cortex (McTeague et al., 2017).

## Methods

### Identification of studies

Since transdiagnostic meta-analyses have been published recently and therefore have conducted systematic literature searches up to 2018, we first extracted data from reference lists (Janiri et al., 2020; Sprooten et al., 2017). We also extracted data from the BrainMap database. We then conducted a literature search in accordance with the Preferred Reporting Items for Systematic Reviews and Meta-analyses criteria (http://www.prisma-statement.org/) to include studies published between 2018 and April 2022. The literature search was conducted on April 25th, 2022, in Embase (via Ovid), PubMed, and Web of Science (see online Supplementary Figure 1 and online Supplementary Figure 2 for the PRISMA flowcharts). The keywords used were as follow: ‘(schizophrenia OR psychosis OR bipolar OR depression) AND (fMRI OR functional magnetic resonance imaging) AND (task OR paradigm OR performance OR event-related)’. Results were restricted to studies published in 2018 or later and written in English or French. Inclusion criteria were: (1) original manuscript from a peer-reviewed journal, (2) functional magnetic resonance imaging (fMRI) studies that included a fMRI task, (3) whole-brain between-diagnoses results, (4) participants meeting DSM/ICD criteria for SCZ, MDD or BD. Included studies from the literature search can be found in online Supplementary Table 1 with the studies included based on reference lists from previous meta-analyses (Janiri et al., 2020; Sprooten et al., 2017).

### Meta-analytic approach

The task contrasts from each experiment were manually annotated and sorted in four non-mutually exclusive categories: (1) positive affect (i.e. response to positive stimuli); (2) negative affect (i.e. response to negative stimuli); (3) cognitive component (i.e. working memory processes, attentional processes, etc.); (4) social processes (i.e. social cognition task) (Sanislow et al., 2010; Sprooten et al., 2017).

Modeled activation map was created for each meta-analytic experiment (2 mm^3^ resolution), converted into a 1D feature vector of voxel values (2 mm^3^ grey matter mask in Montreal Neurological Institute (MNI) space), and concatenated together to form an experiment (*e*) by voxel (v) matrix (762 experiments by 226 654 voxels). Pairwise correlation was conducted between the 1D feature vector of each experiment to obtain spatial similarity between maps. Then, we identified meta-analytic experiments that showed similar brain topographic map, namely meta-analytic groupings (MAGs), through a Correlation-Matrix-Based Hierarchical Clustering (see (Dugré et al., 2022; Laird et al., 2015)). The hierarchical clustering was carried out using correlation distance (1 – *r*) and complete linkage method. We examined the most optimal number of clusters using the silhouette, Calinski–Harabasz indices, and adjusted rand index for a *k* range of 2–15 clusters (Eickhoff, Thirion, Varoquaux, & Bzdok, 2015). More precisely, at each *k*, metrics were compared against a null distribution of random spatial arrangement. To do so, 2500 datasets were created artificially by shuffling foci locations across meta-analyses but preserving original meta-analyses’ properties (e.g. number of foci, sample size). The average of each metric (i.e. silhouette and Calinski–Harabasz indices) derived from true dataset, was compared against the artificially created null distribution and then plotted for *k* range of 2–15 clusters. This improves our ability to select the most optimal *k* by considering the probabilities of getting a certain metric value in a random spatial arrangement. Given that the ground truth class labels are unknown, we compared, for each *k*, the consistency (adjusted rand index) between Label_TRUE_ with the Label_NULL_ then averaged across the 2500 iterations. A local minimum in the plots suggests a decrease in overlap between both sets of labels. We tested the best solution across different correlation distance (i.e. Spearman, Pearson) and linkages (i.e. average, complete). Each author gave their interpretation of the best fit for all three indices and metrics until a consensus was reached. After having found the optimal number of clusters, we randomly removed 10% of the meta-analyses and re-ran the clustering algorithm until the labels replicated in at least two consecutive iterations of 1000 repetitions, as a stopping rule. This was done to select the most stable labeling solution for a final hierarchical clustering (online Supplementary Figure 3). All these analyses were performed using Scikit-learn (version 0.21.3) in Python (version 3.7.4) (Pedregosa et al., 2012). Finally, clusters involving less than 1% were removed from subsequent analyses.

An activation likelihood estimation (ALE) meta-analysis was conducted on experiments within each of the resulting MAGs to extract their aberrant activation maps (see online Supplementary Material). Then, results *within* each MAG were thresholded using *p* < 0.001 uncorrected at a voxel-level with a family-wise correction *p* < 0.05 at a cluster-level. Between-group differences in probabilities of activation were tested at a network-level and region-level for hyperactivation and hypoactivation studies separately using Kruskal–Wallis with a threshold of *p* < 0.05 uncorrected. False-discovery rate (FDR) corrected results are also available in online Supplementary Material (Benjamini & Hochberg, 1995).

To allow comparison between results from the data-driven and the classical approaches, we conducted a meta-analysis on each disorder across tasks (see online Supplementary Material for more details and results).

To investigate the effect of confounding variables on the results, we extracted probabilities of activation for each region of each MAG. We performed Spearman rank correlations to assess potential relationships between results and age, sex, and medication rate.

### Functional decoding of meta-analytic groupings

The resulting MAGs were functionally characterized by Neurosynth meta-analytic term-based decoding (Yarkoni, Poldrack, Nichols, Van Essen, & Wager, 2011). This method uses Pearson correlation between two vectorized maps (user input and the Neurosynth meta-analytic maps). Top 10 most correlated terms were extracted.

Furthermore, we sought to examine the contribution of canonical networks in the resulting MAGs. We conducted network mapping approach to investigate the connectivity network linking peak coordinates of studies within each MAG (Peng, Xu, Jiang, & Gong, 2022; Stubbs et al., 2023). Briefly, a 4-mm sphere was created around each coordinate from each study to create a study-level mask. Then, we computed the normative functional connectivity map of each study-level mask using preprocessed resting-state data of 1000 healthy subjects (ages 18 to 35 years old, 50% females) of the Brain Genomics Superstruct Project (Holmes et al., 2015; Thomas Yeo et al., 2011). Information about preprocessing steps are available elsewhere (Thomas Yeo et al., 2011). First, we averaged the time-course of voxels within each of the study-level mask (4 mm spheres) to the time course of every other voxel in the brain for each of the 1000 healthy participants, yielding a subject-level study map (1000 participants × MAG_studies_). A group-level connectivity map was then computed using a voxel-wise one-sample *t* test across the 1000 participants for each study within a MAG. A subsequent voxel-wise one-sample *t* test using the unthresholded study-level maps was conducted to generate a resting-state connectivity map per MAG that is more consistent than expected by chance through permutation testing (Winkler, Ridgway, Webster, Smith, & Nichols, 2014). Contribution of each Schaefer's seven networks (Schaefer et al., 2018) and subcortex (Fischl et al., 2002) was then examined by computing its effect size compared to what was found outside a given network (Cohen's *d*).

## Results

### Meta-analytic groupings

Literature search yielded 762 experiments derived from 566 original fMRI studies (see online Supplementary Figure 1 and 2 for the PRISMA flowcharts and online Supplementary Table 1 for more details on the included studies). Most experiments included fMRI tasks associated with cognitive systems (*k* = 494), negative valence (*k* = 191), positive valence (*k* = 180), and social cognition (*k* = 180). Included studies involved 13 991 patients with a mean age of 34.33 years old (s.d. = 7.13). The average sex ratio across studies was 56.20% female, and the average rate of medication was 76.69%. Details on demographic per psychiatric disorder can be found in Table 1.

**Table 1.** Demographic and clinical characteristics of participants per psychiatric disorder

	SCZ	MDD	BD	Statistic	*p*-Value	Post-hoc
*n* cases	7948	3659	2384			
Mean age	33.50	35.84	35.10	*F*(2, 593) = 6.18	0.002	MDD > SCZ (*p* = 0.003)
Sex ratio (% male)	64.91	38.91	49.04	*F*(2, 587) = 72.52	<0.001	SCZ > BD > MDD (*p* < 0.001)
Medication rate (% medicated)	90.82	40.09	75.82	*H*(2, 537) = 175.21	<0.001	SCZ > BD > MDD (*p* < 0.001)

**Figure 1.**
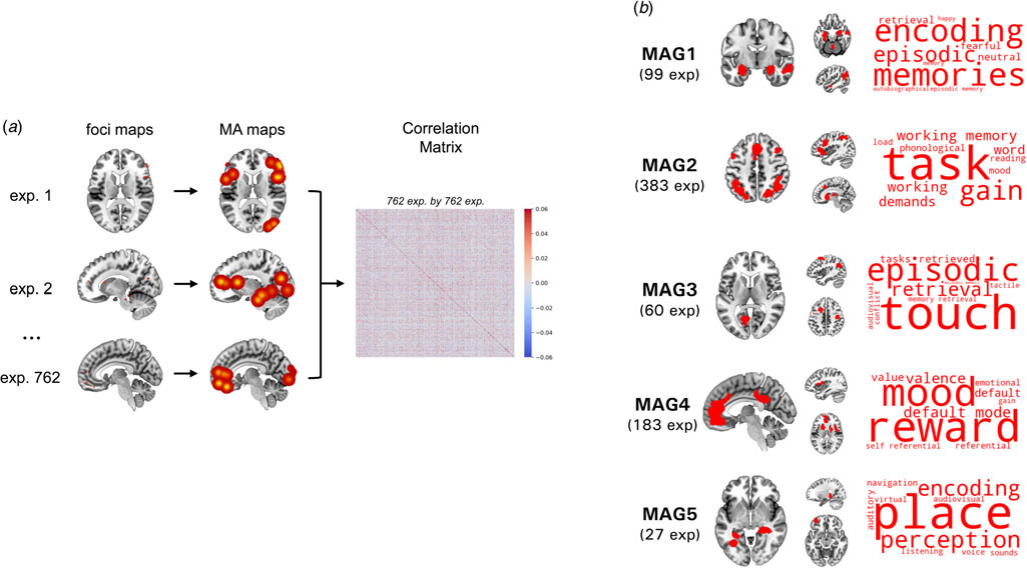
Summary of the meta-analytic groupings. (a) Calculating Similarity between Aberrant Activation Maps, (b) Identifying Main Meta-Analytic Groupings. *Note.* Wordclouds represent associations between MAGs and Neurosynth meta-analytic terms. Only the top 10 terms are shown. Font size illustration correlational strength.

As mentioned earlier, we investigated the clustering solutions for a range of *k* = 2–15 MAGs. Based on consensus, the Silhouette, the Calinski–Harabasz and the adjusted rand indices, clustering analyses revealed that the 7-MAG and the 11-MAG solutions were the most optimal. Stable label solutions were found after 10 and 31 iterations, respectively. We focused on the 7-MAG solution for parsimonious reasons (see Table 2 and Fig. 1 for results of the 7-clusters solution, online Supplementary Figure 3 and 4 and online Supplementary Table 2 for results of the metrics, and online Supplementary Figure 5 and online Supplementary Table 3 for details on the 11-MAG solution). However, due to the limited number of studies of two MAGs (<5%), only findings on the five MAGs are reported. The metrics for the 7-MAG solution were as follow: Silhouette *z* = 1.32, Calinski *z* = 1.62, and adjusted rand index *z* = 0.47. No differences were found across MAGs for age distribution (*F*(4, 736) = 1.59, *p* = 0.174), sex ratio (F(4, 728) = 2.07, *p* = 0.083), and rate of medication (H(4, 676) = 2.59, *p* = 0.629). MAGs did significantly differ in experiments annotated as part of cognitive systems (*X*^2^(4, 752) = 19.42, *p* < 0.001; MAG2 > MAG1 > MAG5), negative valence systems (*X*^2^(4, 752) = 11.08, *p* = 0.026; MAG3 > MAG2), and social processes (*X*^2^(4, 752) = 14.85, *p* = 0.005; MAG4 and MAG2 > MAG1), but not positive valence systems (*X*^2^(4, 752) = 6.78, *p* = 0.148).

**Table 2.** Meta-analytic grouping results (five-cluster solution)

Regions	L/R	MNI coordinates			ALE values	Cluster size (mm^3^)	Hyper-hypoactivation combined	
		x	y	z			Statistic	*p*-Value uncorrected
*MAG1*								
Network							*H*(2, 99) = 4.20	0.122
AMY/aHIP	R	26	−4	−16	0.02956	4552	*H*(2, 99) = 4.55	0.103
AMY/aHIP	L	−26	−10	−18	0.02734	7600	*H*(2, 99) = 1.74	0.418
Lobule V	R	2	−58	−16	0.02297	1328	*H*(2, 99) = 2.78	0.25
MTG	R	56	−8	−16	0.02219	2112	*H*(2, 99) = 0.82	0.664
IPL	R	50	−60	20	0.02413	5880	*H*(2, 99) = 12.79	0.002**
*MAG2*								
Network							*H*(2, 395) = 6.50	0.039**
dlPFC	L	−46	8	32	0.04794	8264	*H*(2, 395) = 2.28	0.32
Ains	L	−38	24	−2	0.04324	5368	*H*(2, 395) = 7.59	0.022*
SPL	L	−34	−56	46	0.04139	3872	*H*(2, 395) = 0.14	0.932
Thalamus	L	−4	−8	6	0.04063	6576	*H*(2, 395) = 5.80	0.055
aMCC	-	0	16	42	0.04595	9720	*H*(2, 395) = 3.41	0.182
aINS	R	34	22	−2	0.04506	25 288	*H*(2, 395) = 3.39	0.184
SPL	R	34	−54	50	0.04482	7288	*H*(2, 395) = 1.87	0.393
*MAG3*								
Network							*H*(2, 60) = 1.52	0.468
AG	L	−44	−60	28	0.02048	2720	*H*(2, 395) = 0.47	0.792
Frontal eye field	L	−30	10	52	0.02032	6312	*H*(2, 395) = 0.06	0.969
Intra-calcarine cortex	L	−10	−66	8	0.01831	2336	*H*(2, 60) = 2.87	0.238
Precentral gyrus	R	40	−16	46	0.02185	1776	*H*(2, 60) = 2.55	0.279
*MAG4*								
Network							*H*(2, 183) = 11.45	0.003**
Striatum	L	−16	12	10	0.03271	4592	*H*(2, 183) = 12.96	0.003**
pgACC/vmPFC	L	−2	42	4	0.03099	19 184	*H*(2, 183) = 12.96	0.002**
Striatum	R	18	2	10	0.02753	3000	*H*(2, 183) = 5.91	0.51
PCC	L	−4	−40	30	0.03032	3192	*H*(2, 183) = 9.32	0.009**
Central opercular	R	42	4	10	0.02733	2960	*H*(2, 183) = 2.01	0.366
*MAG5*								
Network							*H*(2, 27) = 2.93	0.231
Lingual gyrus	L	−28	−54	2	0.01308	4616	*H*(2, 27) = 3.02	0.222
pHIP	R	28	−34	0	0.01417	4552	*H*(2, 27) = 1.56	0.458
Lateral PFC	L	−34	50	−12	0.01367	1392	*H*(2, 27) = 1.21	0.546

*Note.* MNI, Montreal Neurological Institute; MAG, Meta-analytical Grouping; L, Left; R, Right; FDR, False-Discovery Rate; AMY, Amygdala; aHIP, Anterior Hippocampus; pHIP, Posterior Hippocampus; MTG, Middle Temporal Gyrus; pgACC/vmPFC, Perigenual Anterior Cingulate Cortex and Ventromedial Prefrontal Cortex; dlPFC, Dorsolateral Prefrontal Cortex; aMCC, Anterior Midcingulate Cortex; SPL, Superior Parietal Lobule; AG, Angular Gyrus; IPL, Inferior Parietal Lobule; PCC, Posterior Cingulate Cortex; aINS, Anterior Insula. ALE values, measure of coherency across experiments. *Significant with a threshold of *p* < 0.05 uncorrected, **Significant with a corrected threshold of pFDR<0.05 (See online Supplementary Table 4 for more details).

#### MAG1

The MAG1 was characterized by 99 experiments (2356 patients). Mean age of patients was 34.18 years old (s.d. = 7.36), the average sex ratio across studies was 54.25% female, and the average rate of medication was 81.40%.

**Figure 2.**
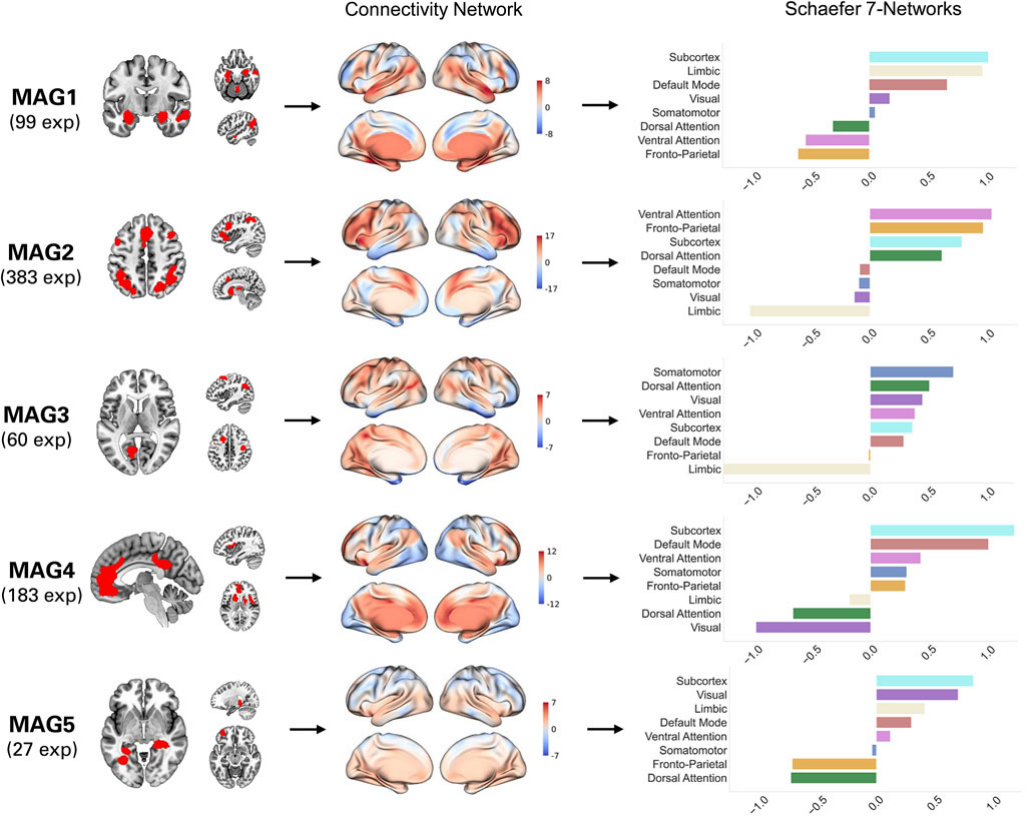
Summary of MAGs and their connectivity network (t-maps). Contribution of each of the Schaefer-400 7 Networks is represented by Cohen's *d*.

It was mainly composed by impaired activity in bilateral amygdala & hippocampus, lobule V, inferior parietal lobule, and middle temporal gyrus. Associations with Neurosynth meta-analytic terms revealed that this MAG was mainly linked to terms including memories, encoding, episodic, retrieval, neutral, fearful, episodic memory, autobiographical, memory, and happy. This MAG also showed associations with the limbic network and the subcortex (Fig. 2).

#### MAG2

The MAG2 was characterized by 383 experiments (8402 patients). Mean age of patients was 33.88 years old (s.d. = 6.87), the average sex ratio across studies was 57.01% female, and the average rate of medication was 76.22%.

The MAG2 was represented by altered brain functioning in dlPFC, bilateral anterior insula, bilateral superior parietal lobule, thalamus, and anterior median cingulate cortex (Table 2 and Fig. 1). Main Neurosynth meta-analytic terms included task, gain, working memory, working, demands, word, phonological, mood, reading, and load. MAG2 was also linked to the fronto-parietal network and the ventral attention network (Fig. 2).

#### MAG3

The MAG3 was composed of 60 experiments (1373 patients). Mean age of patients was 35.29 years old (s.d. = 5.94), the average sex ratio across studies was 51.46% female, and the average rate of medication was 73.43%.

The MAG3 was characterized by aberrant activity mostly in angular gyrus, frontal eye field, intra-calcarine cortex, and precentral gyrus (Table 2 and Fig. 1). Voxelwise associations with Neurosynth decoding revealed that this MAG correlated with touch, episodic, retrieval, retrieved, tasks, memory retrieval, conflict, audiovisual, tactile, and episodic memory. This MAG showed associations with two connectivity networks namely the dorsal attention network and the somatomotor network (Fig. 2).

#### MAG4

The MAG4 included 183 experiments (4445 patients). Mean age of patients was 34.93 years old (s.d. = 7.32), the average sex ratio across studies was 52.53% female, and the average rate of medication was 76.10%.

The MAG4 was mainly composed by abnormal activity in the mesolimbic system, more precisely in the bilateral associative striatum, the posterior cingulate cortex, perigenual anterior cingulate cortex/ventromédian prefrontal cortex (pgACC/vmPFC), and the central opercular (Table 2 and Fig. 1). Neurosynth meta-analytic terms indicated strong association between this MAG and reward, mood, default mode, valence, default, value, referential, emotional, self-referential, and gain. This MAG was linked to the default mode network and the subcortex (Fig. 2).

#### MAG5

The MAG5 was composed of 27 experiments (483 patients). Mean age of patients was 36.42 years old (s.d. = 6.05), the average sex ratio across studies was 62.77% female, and the average rate of medication was 77.70%.

MAG5 included lingual gyrus, posterior hippocampus, and lateral prefrontal cortex. Associations were found between MAG5 and Neurosynth meta-analytic terms such as place, perception, encoding, navigation, auditory, listening, audiovisual, voice, virtual, and sounds. Associations were made between MAG5 and the visual network and the subcortex (Fig. 2).

### Differences between psychiatric disorders

#### Descriptive and task domains

There were differences in number of studies per diagnosis for positive valence systems (*X*^2^(2, 762) = 54.64, *p* < 0.001), negative valence systems (*X*^2^ = 37.81 *p* < 0.001), and cognitive systems (X^2^ = 47.43, *p* < 0.001) but not social processes (X^2^ = 1.24, *p* = 0.539) (Table 3). For positive valence systems, distribution of experiments was equivalent to MDD > SCZ > BD. Moreover, SCZ has higher rates of studies involving negative valence systems, whereas MDD has lower rates in cognitive systems. It is important to note this information to ensure there is not a bias in the type of task administered, which could skew the results.

**Table 3.** Distribution of disorder experiments across task domains per meta-analytic groupings

Disorders by domains	Total	MAG1	MAG2	MAG3	MAG4	MAG5
**Social processes**	**180 (23.6%)**	**38 (38.4%)**	**79 (20.0%)**	**16 (26.7%)**	**40 (21.9%)**	**5 (18.5%)**
Schizophrenia	105 (24.1%)	24 (44.4%)	49 (20.3%)	6 (21.4%)	21 (23.3%)	3 (18.8%)
Major depressive disorder	38 (20.8%)	9 (31.0%)	16 (21.1%)	5 (23.8%)	6 (12.8%)	2 (33.3%)
Bipolar disorder	37 (25.7%)	5 (31.3%)	14 (21.2%)	5 (45.5%)	13 (28.3%)	0 (0.0%)
**Positive valence systems**	**180 (23.6%)**	**28 (28.3%)**	**86 (21.8%)**	**18 (30.0%)**	**43 (23.5%)**	**2 (7.4%)**
Schizophrenia	64 (14.7%)	10 (18.5%)	37 (11.5%)	5 (17.9%)	10 (11.1%)	0 (0.0%)
Major depressive disorder	77 (42.1%)	13 (44.8%)	33 (15.4%)	11 (52.3%)	17 (36.2%)	2 (33.3%)
Bipolar disorder	39 (27.1%)	5 (31.3%)	16 (24.2%)	2 (18.2%)	16 (34.8%)	0 (0.0%)
**Negative valence systems**	**191 (25.1%)**	**31 (31.3%)**	**82 (20.8%)**	**23 (38.3%)**	**47 (25.7%)**	**5 (18.5%)**
Schizophrenia	74 (17.0%)	10 (18.5%)	37 (15.4%)	7 (25.0%)	18 (20.0%)	1 (6.3%)
Major depressive disorder	72 (39.3%)	15 (51.7%)	27 (35.5%)	11 (52.4%)	13 (27.7%)	4 (66.7%)
Bipolar disorder	45 (31.3%)	6 (37.5%)	18 (27.3%)	5 (45.5%)	16 (34.8%)	0 (0.0%)
**Cognitive systems**	**449 (58.9%)**	**45 (45.5%)**	**236 (59.7%)**	**27 (45.0%)**	**112 (61.2%)**	**22 (81.5%)**
Schizophrenia	289 (66.4%)	26 (48.1%)	166 (68.9%)	17 (60.7%)	61 (67.8%)	15 (93.8%)
Major depressive disorder	68 (37.2%)	11 (37.9%)	25 (32.9%)	5 (23.8%)	22 (46.8%)	2 (33.3%)
Bipolar disorder	92 (63.9%)	8 (50.0%)	45 (68.2%)	5 (45.5%)	29 (63.0%)	5 (100.0%)
**Total experiments**	**762 (100.0%)**	**99 (13.0%)**	**395 (51.8%)**	**60 (7.9%)**	**183 (24.0%)**	**27 (3.5%)**
Schizophrenia	435 (100.0%)	54 (12.4%)	241 (55.4%)	28 (6.4%)	90 (20.7%)	16 (3.7%)
Major depressive disorder	183 (100.0%)	29 (15.8%)	76 (41.5%)	21 (11.5%)	47 (25.7%)	6 (3.3%)
Bipolar disorder	144 (100.0%)	16 (11.1%)	66 (45.8%)	11 (7.6%)	46 (31.9%)	5 (3.5%)

*Note.* MAG, meta-analytical grouping.

After Bonferroni correction, post hoc analyses revealed that disorders significantly differed in the number of experiments coded as positive valence systems in MAG4 (*X*^2^(2, 762) = 7.88, *p* = 0.019). Also, the between-diagnoses effect found for negative valence systems was driven by MAG2 (*X*^2^(2, 762) = 11.31, *p* = 0.004) and MAG4 (X^2^(2, 762) = 7.88, *p* < 0.019). Finally, between-diagnoses effect observed for cognitive systems was not mainly driven by any MAG.

#### Neurobiological differences between psychiatric disorders

**Figure 3.**
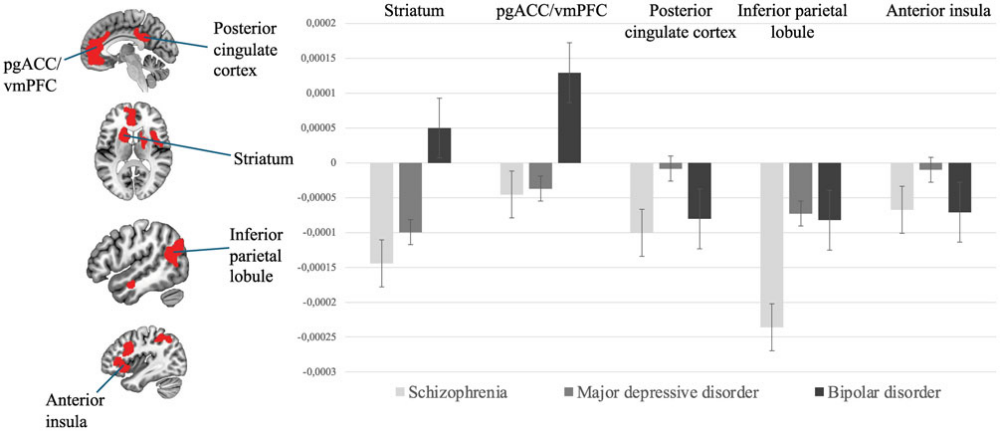
Significant differences between psychiatric disorders regarding their probabilities of activation. *Note.* vmPFC = ventromedial prefrontal cortex; pgACC = perigenual anterior cingulate cortex. Bars represent standard error.

*MAG1*. At a network-level, psychiatric disorders did not significantly differ in the probabilities of combined hyper and hypoactivation (*H*(2, 99) = 4.20, *p* = 0.122). At a region-level, differences were observed in probabilities of activation only in the inferior parietal lobule (*H*(2, 99) = 12.79, *p* = 0.002) (Fig. 3). Inspection of probabilities of activation indicated higher probabilities of decreased activation in SCZ compared to BD (*p* = 0.002). No other significant between-diagnoses differences were observed. For all MAGs, results from between-diagnoses comparison corrected with FDR are available in online Supplementary Table 4.

We found no significant association with age, sex or medication rate and regions of this MAG (all psFDR > 0.1) (online Supplementary Table 5).

*MAG2*. At a network-level, psychiatric disorders significantly differed in the probabilities of combined hyper-hypoactivation (*H*(2, 383) = 6.50, *p* = 0.039). Breakdown of this results highlighted higher probabilities of decreased activation in SCZ compared to MDD (*p* = 0.036). At a region-level, a significant between-diagnoses difference was observed for the right anterior insula extending to the inferior frontal gyrus (*H*(2, 383) = 7.59, *p* = 0.022) (Fig. 3). This cluster was associated with higher probabilities of decreased activation in the SCZ group compared to the MDD group (*p* = 0.038).

We found no significant association with age, sex, or medication rate and regions of this MAG (all psFDR > 0.056) (online Supplementary Table 5).

*MAG3*. At a network-level, psychiatric disorders did not significantly differ in the probabilities of activation across combined hypo-hyperactivation studies (*H*(2, 60) = 1.52, *p* = 0.468). At a region-level, no differences were noted (all ps > 0.1). However, it should be noted that MAG3 only relied on 60 experiments in total, regardless of diagnosis.

We found no significant association with age, sex, or medication rate and regions of this MAG (all psFDR > 0.1) (online Supplementary Table 5).

*MAG4*. At a network-level, psychiatric disorders did significantly differ in the probabilities of activation for the combined hyper-hypoactivation (*H*(2, 183) = 11.45, *p* = 0.003). Breakdown of the direction highlighted higher probabilities of hyperactivation of this network in BD compared to SCZ (*p* = 0.002). At a region-level, psychiatric disorders did significantly differ in the voxel-wise probabilities of activation in the vmPFC/pgACC (*H*(2, 183) = 12.96, *p* = 0.002), the left striatum (*H*(2, 183) = 11.58, *p* = 0.003), the posterior cingulate cortex (*H*(2, 183) = 9.32, *p* = 0.009), and the right striatum cluster (*H*(2, 183) = 5.951, *p* = 0.051). Regarding the vmPFC/pgACC cluster, breakdown of direction and posthoc comparisons revealed that probabilities of hyperactivation were significantly greater in BD than SCZ (*p* = 0.001) as well as BD compared to MDD (*p* = 0.020). Probabilities of hyperactivation were also noted for BD compared to SCZ for the left striatum. SCZ displayed higher probabilities of hypoactivation of the posterior cingulate cortex compared to both BD and MDD (*p* = 0.041 and *p* = 0.034, respectively). Posthoc analyses showed greater probabilities of activation in SCZ within the right striatum, compared to MDD (*p* = 0.021).

We found an association between age and probabilities of activation of the network (*r* = −0.205, pFDR = 0.036) (online Supplementary Table 5).

*MAG5*. At a network-level, psychiatric disorders did not significantly differ in the probabilities of activation across combined hypo-hyperactivation studies (H(2, 27) = 2.93, *p* = 0.231). At a region-level, no differences were noted (all ps > 0.2). However, it should be noted that MAG5 only relied on 27 experiments in total, regardless of diagnosis.

We found no significant association with age, sex, or medication rate and regions or network of this MAG (all psFDR > 0.1) (online Supplementary Table 5).

## Discussion

In this meta-analysis, we conducted hierarchical clustering analysis of abnormal task-related brain activity in SMDs to take into account heterogeneity between studies. This resulted in a 5-cluster solution. MAG1 was mainly associated abnormal brain activity of the limbic system (amygdala, hippocampus, etc.). MAG2 was linked to cognitive functions and was characterized by abnormal activity in bilateral anterior insula and left dorsolateral prefrontal cortex, while MAG3 was mostly associated with altered activity in motor regions (precentral gyrus, intra-calcarine cortex). MAG4 was linked to reward processing and abnormal activity of the mesolimbic system. MAG5 was associated with three regions namely the lingual gyrus, the parahippocampal gyrus and the lateral prefrontal cortex. For most regions of MAG1, no differences were noted between groups. MAG2 differed at a network-level, with SCZ displaying greater probabilities of hypoactivation than MDD. MAG3 and MAG5 displayed common alterations which may be due to the lack of power for these specific analyses. MAG4 revealed that BD was associated with greater probabilities of activation of the vmPFC/pgACC and the left striatum in comparison to SCZ, and greater probabilities of activation of the right striatum in SCZ in comparison to BD. SCZ showed higher level of hypoactivation than MDD in the posterior cingulate cortex. MAG4 differed at a network level, with BD presenting higher probabilities of hyperactivation of the whole network compared to SCZ.

Coherently with the past literature, SMDs depicted mostly common disruptions in MAG1 associated with emotion processing. MAG1 included bilateral amygdala and hippocampus, posterior middle temporal gyrus, lobule V, and inferior parietal lobule. However, differences were noted for the inferior parietal lobule which was hypoactivated in SCZ compared to BD. The inferior parietal lobule is implicated in self-other discrimination which is known to be altered in SCZ (Uddin, Molnar-Szakacs, Zaidel, & Iacoboni, 2006). On the other hand, the amygdala plays a key role in emotion processing, especially in rapid processing of facial expression and identification of negative stimuli (Davis & Whalen, 2001). Alertness of the amygdala is known to be in response to a fearful or unpleasant stimulus which can be modulated by the vmPFC via its direct projections (Diekhof, Geier, Falkai, & Gruber, 2011). Reactivity of the amygdala appears to be linked to automatic negative evaluations of facial expressions (negative bias) (Dannlowski et al., 2007). Notably, SMDs are associated with more negative rating tendencies (negative bias) that can be seen when evaluating neutral, ambiguous, negative stimuli, or even positive stimuli (Suslow, Roestel, & Arolt, 2003). Neuroimaging studies including patients with SMDs suggested the amygdala-hippocampal interaction could play a role in strengthening memory for negative information and increasing negative bias (Hamilton & Gotlib, 2008). Therefore, MAG1 deficits may represent the mechanisms underlying impaired emotional reactivity and/or negative bias across SMDs. Common alterations of the MAG1 are consistent with the shared anxio-depressive symptomatology observed across SMDs (Achim et al., 2011; Lamers et al., 2011; Yapici Eser et al., 2018).

Based on clinical observations, one may expect to observe larger alteration in mood disorders compared to SCZ; however, the evidence supporting this assumption remains unclear. In the meta-analysis from McTeague et al. (2020), no direct comparison was performed between MDD and the other two SMDs, since no spatial convergence was observed in MDD. Sprooten et al. (2017) also conducted a meta-analysis in SMDs, which adopted a similar approach to ours in that they performed analyses regardless of the tasks used in the scanner. Their results were similar to ours in that they found common alterations in the amygdala and hippocampus (among others), and that no differences between SMDs were observed in these regions when restricting analyses to whole-brain fMRI studies.

MAG2 included dlPFC, thalamus, anterior midcingulate cortex, bilateral superior parietal lobule, and anterior insula extending to the inferior frontal gyrus. These regions have been previously linked to cognitive functions such as cognitive control, response inhibition, semantic processing, and working memory (Levy & Wagner, 2011). Such functions are known to be impaired at varying degrees in SMDs (Sheffield et al., 2018). Notably, SCZ showed higher probabilities of hypoactivation of the overall network in comparison to MDD, which is coherent with past literature suggesting more severe cognitive deficits in SCZ than in mood disorders (Sheffield et al., 2018). Moreover, we found hypoactivation of a cluster located in the left anterior insula extending to the inferior frontal gyrus in SCZ compared to MDD. Notably, this cluster plays an important role in language functions, which are known to be altered and associated with formal thought disorder in SCZ (Maderthaner et al., 2023). Indeed, a recent meta-analysis from our team found alterations of the left inferior frontal gyrus in patients with SCZ during verbal tasks (Pilon, Boisvert, & Potvin, 2024). In theory, it has been proposed the prefrontal alterations underlying the cognitive deficits of SCZ would result from an imbalance between glutamatergic and GABAergic neurotransmission (Xu & Wong, 2018). In their neuroimaging meta-analysis on cognitive control studies, McTeague et al., found that the impaired activation of the left lateral prefrontal cluster was mostly associated with SCZ in comparison to other disorders (McTeague et al., 2017). Although our results appear similar to the results obtained by McTeague et al. (2017), it is important to point out that our left lateral prefrontal cluster was more ventral. A potential reason for this small discrepancy is that the meta-analysis of McTeague et al. (2017) focused on cognitive control tasks, while our analyses were performed in a task-blind manner.

MAG3 was linked to visual/motor regions and regions implicated in attention such as the frontal eye field, the angular gyrus, the intra-calcarine cortex and the precentral gyrus. MAG5 was also associated with a visual region (lingual gyrus), and cognitive regions such as the lateral prefrontal cortex and the para-hippocampal gyrus. At the network and the region levels, groups did not differ. However, this may be due to the lack of power induced by the lack of studies in these MAGs (60 and 27 experiments, respectively).

MAG4 was associated with default-mode network and reward processing and included fronto-limbic regions such as the bilateral striatum, the pgACC/vmPFC, the posterior cingulate cortex, and the central opercular. BD showed greater probabilities of hyperactivation of the whole network as well as of clusters located in the vmPFC/pgACC and the left striatum in comparison to SCZ. The vmPFC and the striatum are one of the core regions of the mesocorticolimbic brain reward system, and are known to play an important role in social decision-making, processing of social reward and making value-based decisions (Hiser & Koenigs, 2018). Hyperactivation of these regions could be associated with the higher reward sensitivity and responsiveness characterizing BD (Alloy, Olino, Freed, & Nusslock, 2016). This neurobiological mechanism may provide an explanation for the (hypo)manic symptoms of BD, which are characterized by feelings of euphoria, grandiosity, and behavioral impulsivity (compulsive spendings, etc.). The vmPFC and the pgACC are also related to the processing of negative valence stimuli, such as punishment (Santesso et al., 2012). Notably, it has been suggested that both unipolar and bipolar depression was associated with higher sensitivity to punishment (Adida et al., 2011; Eshel & Roiser, 2010). Taken together, the vmPFC/pgACC results are coherent with reward/punishment sensitivity model, which has been proposed to significantly distinguish BD from SCZ. The fact that a difference was noted between the BD and MDD suggests that the reward/punishment model may apply more closely to BD than MDD. It must be noted that the difference in vmPFC activation between BD and SCZ may stem from an increased probability of hyperactivation in BD as well as an increased of hypo-activation in SCZ. This latter result is coherent with a previous meta-analysis from Kuhn and Gallinat (2013), which showed a reduced vmPFC activity at rest in SCZ. Finally, Sprooten et al. (2017) performed a large-scale meta-analysis of fMRI studies performed across psychotic, mood and anxiety disorders. Although they did not adopt clustering approaches, they also observed an effect of diagnosis on medial prefrontal results, which were also influenced by task domain. However, this result was no longer significant when restricting analyses to fMRI studies using whole-brain rather than region-of-interest approaches.

For MAG4, a higher probability of hypoactivation of the posterior cingulate cortex was found in SCZ compared to MDD. This region is a core region of the default-mode network (Greicius, Krasnow, Reiss, & Menon, 2003), as confirmed by the association that we found between MAG4 and the resting-state functional connectivity maps. Considering that the posterior cingulate cortex plays a key role in social cognition, the increased probability of hypo-activations observed in SCZ in this region is coherent with the fact that socio-cognitive deficits are more severe in this disorder, including deficits in theory of mind (Van Neerven et al., 2021). At the neural level, however, results have been equivocal thus far. Indeed, a recent meta-analysis of 428 resting-state functional connectivity studies performed in SMDs showed no significant differences between disorders when examining the whole default-mode network (Grot et al., 2024). However, sub-analyses on specific brain regions belonging to the default-mode network showed a larger proportion of studies reporting alterations in SCZ as compared to BD and MDD. Moreover, some evidence suggests that the posterior default-mode network may be more prominently impaired in SCZ in reward-related contexts (Segarra et al., 2016).

SCZ depicted greater probabilities of hyperactivation of the right associative striatum cluster in comparison to MDD. The striatum plays an important role in reward processing, associative learning and voluntarily movement (Delgado, 2007). In SCZ, several positron emission tomography studies have shown that dopamine release is increased in the associative part of the striatum (McCutcheon, Beck, Jauhar, and Howes, 2018). The associative striatum translates motivational states into behavior while taking perceptual information into account (Liljeholm & O'Doherty, 2012). Thus, increased dopamine release in this part of the striatum could lead to an aberrant attribution of motivational value to irrelevant stimuli by making inappropriate associations (McCutcheon et al., 2019). In turn, such aberrant experiences may give rise to psychotic symptoms (Kapur, 2003; McCutcheon et al., 2019). While the brain regions of MAG4 were mostly associated with reward processing, it is noteworthy that MAG4 emerged from experiments related mostly to cognitive systems (see Table 3). Taken together, these results are consistent with the aberrant salience hypothesis of psychosis, as they show hyperactivations of the associative striatum in SCZ during cognitive contexts having no inherent motivational value (Kapur, 2003; McCutcheon et al., 2019). Methodologically speaking, these results might not have been found had we adopted a classic meta-analysis approach examining task-specific effects.

The data-driven meta-analytical approach adopted here has two main strengths. The first is that it directly tackles heterogeneity across studies. In classical approaches, analyses are based on diagnoses, and results are interpreted as though they had been present in each study involving the disorder of interest. However, meta-analytic findings are frequently driven by a limited number of studies. In fact, a growing number of fMRI meta-analyses found no spatial convergence in pediatric psychiatric disorders (Dugré et al., 2022), attention-deficit-hyperactivity disorder (Samea et al., 2019) and major depression disorder (Gray, Müller, Eickhoff, & Fox, 2020; Müller et al., 2017). Clustering brain alterations based on their similarity, regardless of diagnosis, helps circumvent this regional heterogeneity. Another strength of the approach adopted here is that in SMDs, alterations are frequently observed in brain regions that are not task-related (Taylor et al., 2012). This could be explained by the fact that experimental tasks are rarely purely cognitive or emotional. Coherently with this view, most experiments in each MAG used cognitive tests, even in the case of the MAG1 and MAG4, which elicited limbic and brain reward alterations, respectively. In terms of functional decoding, we identified a cognitive MAG (MAG2) which referred to both language and cognitive control functions, which are normally considered as close but separate functions (De Baene, Duyck, Brass, & Carreiras, 2015). This may explain why we observed a hypo-activation of the language-related left inferior frontal gyrus in schizophrenia. As for MAG4, it comprised alterations of regions of the brain reward system and the default mode network, which are usually considered as separate networks (Dobryakova & Smith, 2022). In this context, we were able to show that the vmPFC is hyperactived in BD, a result consistent with the seminal reward / punishment model of BD.

However, this study has also limitations that should be acknowledged. First, the number of studies available for certain task domains was limited. For instance, there was very few studies on auditory processing, and this may explain why no results were found in auditory cortices which are frequently reported as showing aberrant activity in hallucinated SCZ patients (Ćurčić-Blake et al., 2017). Second, some of the differences between diagnoses were noted at an uncorrected threshold but could not survive correction for multiple comparisons. Moreover, we could not observe differences in MAG3 and MAG5 which could suggest the meta-analysis was underpowered for certain subanalyses examining differences between disorders. Although the current meta-analysis has included 566 studies, several subanalyses had to be performed (5 MAGs × hyper- v. hypo-activations × 3 SMDs). Third, it would be ideal to perform the analyses with a second sample to replicate the results (e.g. split into two halves). However, at full capacity, we already lacked power to detect differences in some MAGs, and therefore we could not perform this analysis. Fourth, ALE only focuses on significant between-diagnosis results, and therefore does not consider negative results. This may have inflated the results and should be taken into account when interpreting the results. Finally, the rate of task domains differed between diagnosis, and this may have confounded results. It is important to point out, however, that the most robust between-diagnosis differences occurred in regions that were not related to the most prevalent task domain used in this subset of experiments (MAG4). As such, this pattern makes it unlikely that results were explained by a task domain confound.

Using a transdiagnostic approach focusing on SMDs, and cluster analyses seeking to handle the heterogeneity of fMRI results, our meta-analysis showed common brain alterations in an emotional network, subtle differences in an attentional network and graded levels of impairment in a cognitive control network (SCZ > MDD and BD). SCZ was associated with increased alterations in the (associative) striatum, a result consistent with the aberrant salience hypothesis of psychosis, while BD was associated with increased alterations in the vmPFC, a result consistent with the reward/punishment model of this mood disorder. Further fMRI studies are required using a transdiagnostic approach in SMDs. In that venue, the spectrum of the emotional or cognitive processes to be investigated will need to be broadened. The clinical characteristics (comorbid symptoms) will need to be better described to be able to perform finer investigations. Finally, additional fMRI task studies need to be carried out in a larger number of psychiatric disorders to enable the pursuit of data-driven meta-analyses using a fully transdiagnostic approach, while performing clustering analyses on neural alterations evoked by specific task categories.

## Supporting information

Boisvert et al. supplementary material Boisvert et al. supplementary material

## Supplementary material

The supplementary material for this article can be found at https://doi.org/10.1017/S003329172400165X.
