# Supplementary material for: Patterns of abnormal activations in severe mental disorders a transdiagnostic data-driven meta-analysis of task-based fMRI studies
Source: Psychol Med. 2024 Oct 14;54(13):3612–23. doi: 10.1017/S003329172400165X (PMC11536122; doi:10.1017/S003329172400165X)
Supplement: Boisvert et al. supplementary material [file S003329172400165Xsup001.docx]

– SUPPLEMENTARY MATERIAL –

**Patterns of abnormal activations in severe mental disorders:**

**a transdiagnostic data-driven meta-analysis of task-based fMRI studies**

Mélanie Boisvert, PhD c^1,2^ *; Jules R. Dugré, PhD^3^ *; Stéphane Potvin, PhD^1,2^

^1^ Research Center of the Institut Universitaire en Santé Mentale de Montréal; Montreal, Canada

^2^ Department of Psychiatry and Addictology, Faculty of medicine, University of Montreal; Montreal, Canada

^3^ School of Psychology and Centre for Human Brain Health, University of Birmingham, Birmingham, UK

* Co-primary authors

**Corresponding authors**

Stéphane Potvin, PhD & Jules R. Dugré; Centre de recherche de l'Institut Universitaire en Santé Mentale de Montréal; 7331 Hochelaga; Montréal, Canada; H1N 3V2; Email: [stephane.potvin@umontreal.ca](mailto:stephane.potvin@umontreal.ca) / [jules.dugre@umontreal.ca](mailto:jules.dugre@umontreal.ca)

Table of Contents

[Supplementary Figure 1. PRISMA flowchart. 3](#_Toc172188131)

[Supplementary Figure 2. PRISMA flowchart for schizophrenia studies between 2014 and 2017. 4](#_Toc172188132)

[Supplementary Table 1. Included studies 5](#_Toc172188133)

[Supplementary Figure 3. Results of the Clustering Analyses showing that the 7-cluster & 11-cluster solutions were the most optimal number of clusters. 50](#_Toc172188134)

[Supplementary Table 2. Metrics for goodness of clustering. 51](#_Toc172188135)

[Supplementary Figure 4. Results of the Clustering Analyses 52](#_Toc172188136)

[Supplementary Figure 5. Meta-analytic Results for the 11-MAG solution (pFWE<0.05). 53](#_Toc172188137)

[Supplementary Table 3. 11-MAG Solution 54](#_Toc172188138)

[Supplementary Table 4. ALE meta-analysis results corrected per Meta-analytical Groupings and results of between-group comparisons of probabilities of activation for each region using a threshold of p < 0.05 corrected with false discovery rate (FDR). 56](#_Toc172188139)

[Supplementary Table 5. Results from the Spearman rank correlations for confondant variables. 59](#_Toc172188140)

[Methods: ALE classical approach 62](#_Toc172188141)

[Results: Disorder-Specific ALE Meta-analyses 63](#_Toc172188142)

[Supplementary Figure 6. ALE meta-analysis on Severe Mental Disorders (Across Tasks, Irrespective of the Directionality) 67](#_Toc172188143)

[Supplementary Figure 7. ALE meta-analysis on Severe Mental Disorders (Across Tasks, Cases > Controls) 68](#_Toc172188144)

[Supplementary Figure 8. ALE meta-analysis on Severe Mental Disorders (Across Tasks, Controls > Cases) 69](#_Toc172188145)

# **Supplementary Figure 1.** PRISMA flowchart.

Web of Science (n=1,797)

PubMed

(n=2,866)

Embase

(n=2,452)

All records (n=7,115)

Records removed (duplicates) (n=2,286)

Records screened (n=4,829)

Records for retrieval (n=436)

Records not retrieved for various reasons (n=196)

Reports excluded for various reasons (n=130):

- Predetermined regions of interest (n=29)
- Incomplete data or no between-group results (n=93)
- Duplicate cohort (n=8)

Full-text records for eligibility (n=240)

Studies included in meta-analysis (n=110)

Records excluded based on inclusion critera (n=4,393)

# **Supplementary Figure 2.** PRISMA flowchart for schizophrenia studies between 2014 and 2017.

Web of Science (n=781)

PubMed

(n=747)

Embase

(n=1,188)

All records (n=2,716)

Records removed (duplicates) (n=1,010)

Records screened (n=1,706)

Records for retrieval (n=368)

Records not retrieved for various reasons (n=175)

Full-text records for eligibility (n=193)

Studies included in meta-analysis (n=67)

Records excluded based on inclusion critera (n=1,338)

Reports excluded for various reasons (n=126):

- Predetermined regions of interest (n=47)
- Incomplete data or no between-group results (n=36)
- Duplicate cohort (n=4)
- Already included (retrieved from BrainMap) (n=29)
- Connectivity study (n=6)
- No SCZ or HC (n=4)

| **Supplementary Table 1.** Included studies | | | | | | | | | | | |
| --- | --- | --- | --- | --- | --- | --- | --- | --- | --- | --- | --- |
| Dx | First author, year | Cases (n=) | HC  (n=) | Mean age | Male  (%) | Medicated  (%) | Task | Pos | Neg | Cogn | Social |
| SCZ | Arrondo 2015 (Arrondo et al., 2015) | 22 | 21 | 32.7 | 86.36 | 100 | Monetary incentive delay task | X |  |  |  |
| SCZ | Anticevic 2011 (Anticevic, Repovs, Corlett, & Barch, 2011) | 28 | 24 | 36.4 | 78.57 | 100 | Delayed response task |  | X | X |  |
| SCZ | Anticevic 2013 (Anticevic, Repovs, & Barch, 2013) | 28 | 24 | 36.4 | 78.57 | 100 | Delayed match-to-sample task |  |  | X |  |
| SCZ | Arce 2006 (Arce et al., 2006) | 17 | 17 | 40.9 | 76.47 | 100 | Go/no-go task |  |  | X |  |
| SCZ | Arcuri 2012 FTD (Arcuri et al., 2012) | 9 | 10 | 33 | 100 | NR | Semantic decision task |  |  | X |  |
| SCZ | Arcuri 2012 no FTD (Arcuri et al., 2012) | 9 | 10 | 38 | 100 | NR | Semantic decision task |  |  | X |  |
| SCZ | Avsar 2011 (Avsar et al., 2011) | 8 | 10 | 31.4 | 100 | 100 | Delayed match-to-sample task |  |  | X |  |
| SCZ | Avsar 2013 (Avsar et al., 2013) | 14 | 14 | 37 | 71.43 | NR | Delay-discounting task |  |  | X |  |
| SCZ | Backes 2011 (V. Backes et al., 2011) | 17 | 17 | 33.7 | 58.82 | 100 | Attentional network test |  |  | X |  |
| SCZ | Bak 2013 (Bak, Rostrup, Larsson, Glenthøj, & Oranje, 2014) | 23 | 20 | 36.4 | 100 | 73.91 | Somatosensory P50 suppression paradigm |  |  | X |  |
| SCZ | Barch 2007 (Barch & Csernansky, 2007) | 57 | 120 | 31.5 | 71.92 | NR | n-back task |  |  | X |  |
| SCZ | Barkataki 2008 with hx violence (Barkataki et al., 2008) | 12 | 14 | NR | 100 | NR | Go/no-go task |  |  | X |  |
| SCZ | Barkataki 2008 without hx violence (Barkataki et al., 2008) | 12 | 14 | NR | 100 | NR | Go/no-go task |  |  | X |  |
| SCZ | Becerril 2011 (Becerril & Barch, 2011) | 38 | 32 | 36.7 | 65.79 | 100 | n-back task |  |  | X |  |
| SCZ | Bedford 2012 (Bedford, Surguladze, Giampietro, Brammer, & David, 2012) | 11 | 8 | 39 | 63.64 | 100 | Self-evaluation task |  |  |  | X |
| SCZ | Bender 2013 (Bender et al., 2013) | 14 | 13 | 35.2 | 57.14 | 100 | Volitional and visually guided saccades |  |  | X |  |
| SCZ | Bjorquist 2013 (Bjorkquist & Herbener, 2013) | 14 | 14 | 31.6 | 71.43 | 78.57 | Emotional processing | X | X |  |  |
| SCZ | Blasi 2010 (Blasi et al., 2010) | 16 | 21 | 32.5 | 81.25 | 100 | Attentional control |  |  | X |  |
| SCZ | Bor 2011 (Bor et al., 2011) | 22 | 15 | 28.4 | 77.27 | 100 | n-back task |  |  | X |  |
| SCZ | Brahmbhatt 2006 (Brahmbhatt, Haut, Csernansky, & Barch, 2006) | 19 | 72 | 21.6 | 89.47 | 100 | Word and face n-back task |  |  | X |  |
| SCZ | Brüne 2011 (Brüne et al., 2011) | 22 | 26 | 26.8 | 63.64 | 100 | Theory of mind task |  |  |  | X |
| SCZ | Camchong 2008 (Camchong, Dyckman, Austin, Clementz, & McDowell, 2008) | 15 | 14 | 38 | NR | 86.67 | Antisaccades and ocular motor delayed response task |  |  | X |  |
| SCZ | Chen 2013 (P.-J. Chen et al., 2013) | 20 | 20 | 29.4 | 50 | NR | Semantic decision task |  |  | X |  |
| SCZ | Choi 2008 (J. W. Choi, Jeong, & Kim, 2008) | 10 | 10 | 29.2 | 30 | 100 | Stroop task |  |  | X |  |
| SCZ | Choi 2012 (J.-S. Choi et al., 2012) | 15 | 16 | 23.5 | 53.33 | 100 | Delayed-response task |  |  | X |  |
| SCZ | Choi 2013 (S.-H. Choi et al., 2013) | 14 | 15 | 29.1 | 57.14 | NR | Imaginary sentence completion test |  |  | X | X |
| SCZ | Cieslik 2013 (Cieslik et al., 2015) | 18 | 18 | 37.1 | 55.56 | 93.75 | Stimulus-response integration |  |  | X |  |
| SCZ | Costafreda 2011 (Costafreda et al., 2011) | 32 | 40 | 35.5 | 81.25 | 93.75 | Verbal fluency task |  |  | X |  |
| SCZ | Cuervo-Lombard 2012 (Cuervo-Lombard et al., 2012) | 13 | 14 | 30.7 | 100 | 100 | Autobiographical memory task |  |  | X |  |
| SCZ | Davalos 2011 (Davalos, Rojas, & Tregellas, 2011) | 16 | 18 | 48.4 | 50 | 87.5 | Auditory time discrimination task |  |  | X |  |
| SCZ | de la Fuente-Sandoval 2010 (de la Fuente-Sandoval, Favila, Gómez-Martin, Pellicer, & Graff-Guerrero, 2010) | 12 | 13 | 23.6 | 83.33 | 0 | Experimental pain-tolerance |  | X |  |  |
| SCZ | Derntl 2012 (Derntl et al., 2012) | 14 | 15 | 34.2 | 64.29 | 100 | Emotion recognition, perspective taking and affective responsiveness | X | X |  | X |
| SCZ | Dodell-Feder 2014 (Dodell-Feder, Tully, Lincoln, & Hooker, 2014) | 20 | 18 | 38.8 | 60 | 90 | False-belief stories |  |  |  | X |
| SCZ | Dyck 2014 (Dyck, Loughead, Gur, Schneider, & Mathiak, 2014) | 16 | 16 | 35.9 | 62.5 | 100 | Mood induction paradigm | X | X |  |  |
| SCZ | Dyckman 2011 (Dyckman et al., 2011) | 18 | 15 | 42 | 72.22 | 100 | Antisaccades task |  |  | X |  |
| SCZ | Eich 2014 (Eich, Nee, Insel, Malapani, & Smith, 2014) | 18 | 18 | 37.9 | 61.11 | 100 | Item-recognition task |  |  | X |  |
| SCZ | Fatjó-Vilas 2012 (Fatjó-Vilas et al., 2012) | 48 | 46 | 38.5 | 70.83 | 100 | n-back task |  |  | X |  |
| SCZ | Foucher 2011 (Foucher et al., 2011) | 17 | 17 | 29.7 | 88.24 | 94.12 | Working memory task |  |  | X |  |
| SCZ | [García-Martí](https://pubmed.ncbi.nlm.nih.gov/?term=Garc%C3%ADa-Mart%C3%AD%20G%5BAuthor%5D) 2012 (García-Martí, 2012) | 22 | 28 | 29.8 | 100 | 100 | Auditory paradigm to replicate AVH | X | X | X |  |
| SCZ | Gizewski 2013 (Gizewski et al., 2013) | 12 | 12 | 37.8 | 100 | 100 | Reading the mind in the eyes test |  |  |  | X |
| SCZ | Gradin 2012 (Gradin et al., 2012) | 13 | 16 | 41.2 | 84.61 | 100 | Cyberball social exclusion task |  | X |  | X |
| SCZ | Griego 2008 (Griego, Cortes, Nune, Fisher, & Tagamets, 2008) | 8 | 10 | 32 | 50 | 100 | Visual matching task |  |  | X |  |
| SCZ | Habel 2010 (Habel, Pauly, et al., 2010) | 14 | 14 | 37.1 | 100 | 95.71 | Emotional n-back task |  |  | X |  |
| SCZ | Hall 2010 (J. Hall et al., 2010) | 15 | 14 | 38.1 | 80 | 100 | Face-name pair memory task |  |  | X |  |
| SCZ | Harrison 2007 (Harrison et al., 2007) | 12 | 14 | 31.6 | 100 | 91.67 | Multi-source interference task |  |  | X |  |
| SCZ | Hasenkamp 2011 (Hasenkamp, James, Boshoven, & Duncan, 2011) | 10 | 10 | 42.5 | 100 | 100 | Simple target detection task |  |  | X |  |
| SCZ | Henseler 2009 (Henseler, Falkai, & Gruber, 2009) | 12 | 12 | 33 | 66.67 | 100 | Delayed match-to-sample task |  |  | X |  |
| SCZ | Holt 2011 (Holt et al., 2011) | 18 | 17 | 35.9 | 66.67 | 83.33 | Self-reflection task |  |  |  | X |
| SCZ | Holt 2012 (Holt, Coombs, Zeidan, Goff, & Milad, 2012) | 20 | 17 | 34.7 | 100 | 60 | Fear conditioning and extinction learning |  | X |  |  |
| SCZ | Hong 2005 (Hong et al., 2005) | 12 | 12 | 40 | 66.67 | 100 | Closed-loop pursuit gain |  |  | X |  |
| SCZ | Hughes 2012 (Hughes, Fulham, Johnston, & Michie, 2012) | 10 | 10 | 35.9 | 70 | 90 | Stop-signal task |  |  | X |  |
| SCZ | Hutcheson 2012 (Hutcheson et al., 2012) | 28 | 28 | 36.7 | 71.43 | 100 | Episodic memory task |  |  | X |  |
| SCZ | Jamadar 2013 (Sharna Jamadar et al., 2013) | 74 | 133 | 36.4 | 79.73 | 59 | The semantic object retrieval task |  |  | X |  |
| SCZ | Jiménez 2010 male (Jiménez, Mancini-Marïe, Lakis, Rinaldi, & Mendrek, 2010) | 16 | 18 | 33.2 | 100 | 100 | Mental rotation task |  |  | X |  |
| SCZ | Jiménez 2010 female (Jiménez et al., 2010) | 17 | 17 | 32.1 | 0 | 100 | Mental rotation task |  |  | X |  |
| SCZ | John 2011 (John, Halahalli, Vasudev, Jayakumar, & Jain, 2011) | 24 | 24 | 30.1 | 66.67 | 62.5 | Semantic category word generation |  |  | X |  |
| SCZ | Joyal 2007 (Joyal et al., 2007) | 12 | 12 | NR | NR | NR | Go/no-go task |  |  | X |  |
| SCZ | Kang 2009 avh (Kang et al., 2009) | 14 | 28 | 29.5 | 50 | 100 | Emotional auditory paradigm | X |  | X |  |
| SCZ | Kang 2009 no-avh (Kang et al., 2009) | 14 | 28 | 30.4 | 50 | 100 | Emotional auditory paradigm | X |  | X |  |
| SCZ | Kerns 2005 (Kerns et al., 2005) | 13 | 13 | 35.6 | 61.54 | 100 | Stroop task |  |  | X |  |
| SCZ | Kim 2010 (J. Kim, Matthews, & Park, 2010) | 12 | 13 | 40.2 | 58.33 | 100 | Delayed match-to-sample task |  |  | X |  |
| SCZ | Kircher 2007 (T. T. J. Kircher, Leube, Erb, Grodd, & Rapp, 2007) | 12 | 12 | 32.8 | 91.67 | 100 | Sentences with metaphoric vs literal meaning |  |  | X |  |
| SCZ | Koch 2008 (K. Koch et al., 2008) | 41 | 41 | 30.2 | 68.29 | 100 | Sternberg task |  |  | X |  |
| SCZ | Koch 2011 (Kathrin Koch et al., 2011) | 19 | 20 | 35.2 | 63.16 | 94.74 | Decision-making under uncertainty |  |  | X |  |
| SCZ | Koeda 2006 (Koeda et al., 2006) | 14 | 14 | 31.6 | 85.71 | 92.86 | Language processing task |  |  | X |  |
| SCZ | Kohler 2008 (Kohler et al., 2008) | 11 | 10 | 35.4 | 45.45 | 100 | Gaze discrimination task |  |  |  | X |
| SCZ | Krabbendam 2009 (Krabbendam et al., 2009) | 11 | 9 | 35.4 | 81.81 | 100 | Stroop task |  |  | X |  |
| SCZ | Kumari 2007 (Kumari et al., 2007) | 29 | 12 | 37.5 | 100 | 100 | Tactile prepulse inhibition paradigm |  |  | X |  |
| SCZ | Lakis 2011 (Lakis et al., 2011) | 37 | 37 | 32.5 | 51.35 | 100 | Emotional recognition paradigm |  | X |  |  |
| SCZ | Lee 2010 (S. J. Lee et al., 2010) | 15 | 18 | 26 | 46.67 | 100 | Empathy | X | X |  | X |
| SCZ | Lee 2014 (J. S. Lee, Chun, Yoon, Park, & Kim, 2014) | 15 | 16 | 36.7 | 60 | 100 | Facial expression task |  |  |  | X |
| SCZ | McIntosh 2008 (McIntosh et al., 2008) | 27 | 37 | 37 | 51.85 | 100 | Imaginary sentence completion |  |  | X |  |
| SCZ | Ragland 2004 (J. Daniel Ragland et al., 2004) | 14 | 15 | 32.7 | 57.14 | 100 | Word encoding/recognition |  |  | X |  |
| SCZ | Shergill 2000 (Shergill, Bullmore, Simmons, Murray, & McGuire, 2000) | 8 | 6 | 32 | 100 | 100 | Inner speech |  |  | X | X |
| SCZ | Simons 2010 (Simons et al., 2010) | 15 | 12 | 34.7 | 100 | 100 | Inner speech |  |  | X | X |
| SCZ | Vercammen 2012 (Vercammen et al., 2012) | 20 | 23 | 34.4 | 75 | 100 | Emotional go/no-go |  | X | X |  |
| SCZ | Walter 2009 (Walter, Kammerer, Frasch, Spitzer, & Abler, 2009) | 16 | 16 | 38 | 50 | 100 | Monetary incentive delay task | X |  |  |  |
| SCZ | Wolf 2011 (D. H. Wolf, 2011) | 26 | 25 | 38 | 53.85 | 96.15 | Forced choice recognition memory paradigm |  |  | X |  |
| SCZ | Wolf 2011b (C. Wolf et al., 2011) | 8 | 8 | 27.6 | 75 | NR | Emotional working memory task |  |  | X | X |
| SCZ | Zhang 2008 no-avh (Zhang et al., 2008) | 13 | 13 | 31.7 | 100 | 0 | Auditory laterality discrimination |  |  | X |  |
| SCZ | Zhang 2008 avh (Zhang et al., 2008) | 13 | 13 | 31.1 | 100 | 0 | Auditory laterality discrimination |  |  | X |  |
| SCZ | Regenbogan 2015 (Regenbogen et al., 2015) | 20 | 24 | 37.3 | NR | NR | Emotional rating |  |  |  | X |
| SCZ | Segarra 2015 (Segarra et al., 2016) | 21 | 21 | 32.2 | 85.71 | 100 | Slot-machine game | X |  |  |  |
| SCZ | Adamczyk 2021 (Adamczyk et al., 2021) | 30 | 30 | 41.9 | 46.67 | 100 | Metaphor comprehension |  |  | X |  |
| SCZ | Bartholomeusz 2018 (Bartholomeusz et al., 2018) | 14 | 22 | 20.4 | 57.14 | 100 | Theory of mind |  |  |  | X |
| SCZ | Berger 2018 (Berger, Bitsch, Nagels, Straube, & Falkenberg, 2018) | 31 | 19 | 32.6 | 70.97 | 77.42 | Humor processing |  |  |  | X |
| SCZ | Briend 2019 (Briend et al., 2019) | 20 | 28 | 39.7 | 65 | NR | Language processing task |  |  | X |  |
| SCZ | Cadena2018 (Cadena, White, Kraguljac, Reid, & Lahti, 2018) | 22 | 20 | 33 | 77.27 | 59.09 | Stroop task |  |  | X |  |
| SCZ | Choudhury2021 (Choudhury et al., 2021) | 42 | 36 | 34.3 | 78.57 | 100 | Speech-gesture combination processing |  |  | X | X |
| SCZ | Creyaufmüller 2020 (Creyaufmüller, Heim, Habel, & Mühlhaus, 2020) | 13 | 12 | 40.7 | 61.54 | 100 | Picture word interference paradigm |  |  | X |  |
| SCZ | Dar 2021 (Dar et al., 2021) | 31 | 17 | 34.7 | 80.65 | 100 | Emotional self-other paradigm, inner speech |  | X |  | X |
| SCZ | De Coster 2019 (De Coster, Lin, Mathalon, & Woolley, 2019) | 23 | 25 | 35.3 | 100 | NR | Theory of mind |  |  |  | X |
| SCZ | Deserno 2020 (Deserno et al., 2020) | 46 | 43 | 35.1 | 69.57 | 100 | Reward-based decision making |  |  | X |  |
| SCZ | Dzafic 2018 (Dzafic, Burianová, Martin, & Mowry, 2018) | 16 | 16 | 46.4 | 56.25 | 100 | Emotional processing |  |  |  | X |
| SCZ | Fryer 2019 (Fryer et al., 2019) | 23 | 72 | 22.5 | 73.91 | 86.96 | Go/no-go |  |  | X |  |
| SCZ | Fuentes-Claramonte 2020 (Paola Fuentes-Claramonte et al., 2020) | 23 | 27 | 37 | 69.57 | 100 | Self-reflection processing |  |  |  | X |
| SCZ | Fuentes-Claramonte 2021 (P. Fuentes-Claramonte et al., 2021) | 70 | 70 | 42.6 | 74.29 | 100 | n-back task |  |  | X |  |
| SCZ | Furuichi 2019 (Furuichi et al., 2019) | 15 | 15 | 27.5 | 53.33 | 100 | Self-other paradigm |  |  |  | X |
| SCZ | Garcia Leon 2021 (Garcia-Leon et al., 2021) | 26 | 30 | 38.5 | 92.31 | 100 | Emotional processing |  | X |  |  |
| SCZ | Gawne 2020 (Gawne et al., 2020) | 22 | 24 | 23.7 | 27.27 | 95.45 | Stroop task |  |  | X |  |
| SCZ | González-Vivas 2020 (González-Vivas et al., 2020) | 35 | 13 | 29.9 | 80 | 100 | Emotional auditory paradigm |  |  | X |  |
| SCZ | Guimond 2018 (Guimond et al., 2018) | 20 | 20 | 26.9 | 60 | 100 | Emotional n-back task |  | X | X |  |
| SCZ | Gurler 2021 (Gurler et al., 2021) | 17 | 17 | 31.2 | 82 | 0 | Memory encoding task |  |  | X |  |
| SCZ | Hahn 2018 (B. Hahn, Robinson, Leonard, Luck, & Gold, 2018) | 37 | 37 | 36.3 | 62.16 | 100 | Working memory task |  |  | X |  |
| SCZ | Hahn2021b (W. Hahn, Domahs, Straube, Kircher, & Nagels, 2021) | 15 | 15 | 38.8 | 86.67 | 100 | Spontaneous speech |  |  | X |  |
| SCZ | He 2021 (He et al., 2021) | 17 | 18 | 33.1 | 76.47 | 94.12 | Speech-gesture combination processing |  |  |  | X |
| SCZ | Herold 2018 (Herold et al., 2018) | 12 | 12 | 36.9 | 50 | 100 | Irony processing |  |  |  | X |
| SCZ | Huang 2019 (A. S. Huang et al., 2019) | 72 | 58 | 27.9 | 66.67 | 100 | Working memory task |  |  | X |  |
| SCZ | Iwashiro 2019 (Iwashiro et al., 2019) | 15 | 23 | 29.9 | 53.33 | 100 | Emotional word recognition |  | X | X |  |
| SCZ | Jia 2020 (Jia et al., 2020) | 38 | 38 | 22.6 | 60.53 | 100 | Perceptual decision-making task |  |  | X |  |
| SCZ | Jimenez 2018 (Jimenez, Lee, Reavis, Wynn, & Green, 2018) | 20 | 16 | 48.3 | 65 | 90 | Social perception |  |  |  | X |
| SCZ | Jimenez 2018 (Jimenez, Lee, Wynn, & Green, 2018) | 20 | 16 | 48.3 | 65 | 90 | Self-referential memory task |  |  | X | X |
| SCZ | Knolle 2018 (Knolle et al., 2018) | 13 | 34 | 23.9 | 75 | 0 | Visual oddball task |  |  | X |  |
| SCZ | Köhler 2019 (Köhler, Wagner, & Bär, 2019) | 28 | 27 | 33.1 | 64.29 | 100 | Stroop task |  |  | X |  |
| SCZ | Kronbichler 2019 (Kronbichler et al., 2019) | 24 | 24 | 26 | 100 | 100 | Visual perspective taking task |  |  |  | X |
| SCZ | Lefebvre 2021 (Lefebvre et al., 2021) | 15 | 19 | 31.5 | 86.67 | 100 | Visual consciousness |  |  | X |  |
| SCZ | Lemmers-Jansen 2019 (Lemmers-Jansen, Fett, Hanssen, Veltman, & Krabbendam, 2019) | 22 | 43 | 19.9 | 63.64 | 73 | Trust game | X |  |  | X |
| SCZ | Li 2018 (Z. Li et al., 2018) | 26 | 26 | 22.8 | 69.36 | 84.62 | Affective incentive delay task + monetary incentive delay task |  | X |  |  |
| SCZ | Li 2019 (X. Li et al., 2019) | 20 | 24 | 23.1 | 65 | 95 | n-back task |  |  | X |  |
| SCZ | Li 2021 (Z. Li et al., 2021) | 52 | 52 | 26.3 | 51.92 | NR | Sensory integration |  |  | X |  |
| SCZ | Liu 2021 (Liu et al., 2021) | 31 | 35 | 25 | 54.83 | 76.47 | Impaired face perception task |  |  |  | X |
| SCZ | Loeb 2018 (Loeb et al., 2018) | 32 | 39 | 21.3 | 50 | NR | n-back task |  |  | X |  |
| SCZ | Lundin 2021 (Lundin et al., 2021) | 31 | 43 | 36.8 | 64.52 | 81 | Delay eyeblink classical conditioning |  | X |  |  |
| SCZ | Martin-Subero 2021 (Martin-Subero et al., 2021) | 27 | 30 | 40.2 | 62.96 | 100 | Autobiographical memory task |  |  | X |  |
| SCZ | Moran 2019 (Moran, Culbreth, Kandala, & Barch, 2019) | 28 | 30 | 37.2 | 67 | 85.71 | Reinforcement-learning task | X |  |  |  |
| SCZ | Moser 2018 (Moser et al., 2018) | 92 | 48 | 27 | 93.5 | 93.5 | n-back task |  |  | X |  |
| SCZ | Oertel 2019 (Oertel et al., 2019) | 27 | 27 | 37.2 | 74.07 | 100 | Associative memory task |  |  | X |  |
| SCZ | Okruszek 2018 (Okruszek et al., 2018a) | 26 | 25 | 35.7 | 50 | 96.15 | Social cognition task |  |  |  | X |
| SCZ | Overbeek 2019 (Overbeek et al., 2019) | 17 | 21 | 23.2 | 76.47 | 100 | Stroop task |  |  | X |  |
| SCZ | Panagiotaropoulou 2019 (Panagiotaropoulou et al., 2019) | 30 | 30 | 26.6 | 83.33 | 100 | Eriksen flanker task |  |  | X |  |
| SCZ | Park 2018 (J. Park, Chun, Park, Kim, & Kim, 2018) | 17 | 20 | 27.2 | 41.18 | 100 | Emotional Stroop task | X | X | X | X |
| SCZ | Park 2019 (J.-I. Park, Kim, Jeong, & Yang, 2019) | 17 | 17 | 30.9 | 52.94 | 100 | Emotional working memory task |  | X | X |  |
| SCZ | Pinkham 2018 (Pinkham, Klein, Hardaway, Kemp, & Harvey, 2018) | 31 | 32 | 35.7 | 58.06 | 87.1 | Emotion recognition |  |  |  | X |
| SCZ | Pretus 2021 (Pretus, Bergé, Guell, Pérez, & Vilarroya, 2021) | 28 | 20 | 37.2 | 64.29 | 100 | Reward paradigm | X |  | X |  |
| SCZ | Rauer 2021 (Rauer, Trost, Petrovic, & Gruber, 2021) | 30 | 40 | 29.8 | 86.67 | 90 | Oddball task |  |  | X |  |
| SCZ | Rodrigue 2018 (Rodrigue, Schaeffer, Pierce, Clementz, & McDowell, 2018) | 23 | 21 | 39.9 | 39.13 | 73.91 | Antisaccade task |  |  | X |  |
| SCZ | Smucny 2018 (Smucny et al., 2018a) | 70 | 53 | 21 | 84.29 | 77.14 | AX version of the continuous performance task |  |  | X |  |
| SCZ | Stäblein 2019 (Stäblein et al., 2019) | 25 | 25 | 36.8 | 68 | 96 | Working memory |  |  | X |  |
| SCZ | Standke 2021 (Standke, Trempler, Dannlowski, Schubotz, & Lencer, 2021) | 22 | 22 | 36.4 | 68.18 | NR | Predictive processing |  |  | X |  |
| SCZ | Stegmayer 2018 (Stegmayer et al., 2018) | 22 | 25 | 37.5 | 63.64 | 100 | Impaired gesture processing |  |  |  | X |
| SCZ | Suttkus 2021 (Suttkus, Schumann, Cruz, & Bär, 2021) | 40 | 58 | 35.3 | 72.5 | 97.5 | n-back task |  |  | X |  |
| SCZ | Tikàsz 2019 (Tikàsz et al., 2019) | 47 | 23 | 34.4 | 100 | 100 | Balloon analogue risk-taking task | X |  | X |  |
| SCZ | Vanes 2018 (Vanes, Mouchlianitis, Collier, Averbeck, & Shergill, 2018) | 21 | 24 | 41.3 | 86 | NR | Reinforcement learning task | X |  |  |  |
| SCZ | Achim 2007 (Achim et al., 2007) | 26 | 20 | 22.6 | 69 | 84.62 | Associative memory task |  |  | X |  |
| SCZ | Calicott 2003 (Joseph H. Callicott et al., 2003) | 14 | 14 | 31.5 | 78.57 | 100 | n-back task |  |  | X |  |
| SCZ | Chung 2016 (Chung & Barch, 2016) | 36 | 27 | 38.96 | 69.4 | 100 | Reward paradigm | X |  |  |  |
| SCZ | Culbreth 2015 (Culbreth, Gold, Cools, & Barch, 2016) | 58 | 40 | 37 | 66.7 | 89.66 | Reversal-learning paradigm | X |  |  |  |
| SCZ | Das 2012 (Das, Lagopoulos, Coulston, Henderson, & Malhi, 2012) | 20 | 19 | 34.5 | 100 | 100 | Theory of mind |  |  |  | X |
| SCZ | Eyler 2004 (Eyler, Olsen, Jeste, & Brown, 2004) | 9 | 10 | 58.9 | 44.44 | 100 | Continuous performance task |  |  | X |  |
| SCZ | Heinze 2006 (Heinze et al., 2006) | 18 | 15 | 35.6 | 61.11 | 100 | Verbal learning and memory task |  |  | X |  |
| SCZ | Hofer 2003 (Hofer et al., 2003) | 10 | 10 | 30.4 | 100 | 0 | Recognition paradigm |  |  | X |  |
| SCZ | Honey 2003 (Honey et al., 2003) | 30 | 27 | 36.9 | 90 | NR | Working memory + psychomotor paradigm |  |  | X |  |
| SCZ | Johnson 2006 (M. R. Johnson et al., 2006) | 18 | 18 | 36.9 | 88.89 | 100 | Sternberg task |  |  | X |  |
| SCZ | Keedy 2009 (Keedy et al., 2009) | 9 | 9 | NR | 66.67 | 0 | Saccade task |  |  | X |  |
| SCZ | Kumari 2010 (Kumari et al., 2010) | 63 | 20 | 37.95 | 74.6 | 100 | Self-monitoring task |  |  |  | X |
| SCZ | Makowski 2016 (Makowski, Lepage, & Harvey, 2016) | 15 | 15 | 33.1 | 73.33 | NR | Social approval task |  |  |  | X |
| SCZ | Manoach 2005 (Manoach et al., 2005) | 16 | 12 | 42 | 87.5 | 87.5 | Visual working memory task |  |  | X |  |
| SCZ | Ragland 2006 (J. Daniel Ragland, Valdez, Loughead, Gur, & Gur, 2006) | 13 | 13 | 35.2 | 84.62 | 100 | Source monitoring task |  |  | X |  |
| SCZ | Salgado-Pineda 2004 (Salgado-Pineda et al., 2004) | 14 | 14 | 25.05 | 50 | 100 | Continuous performance test |  |  | X |  |
| SCZ | Schneider 2007 (Schneider et al., 2007) | 75 | 81 | 32.8 | 100 | 100 | n-back task |  |  | X |  |
| SCZ | Tan 2005 (H.-Y. Tan, Choo, Fones, & Chee, 2005) | 11 | 11 | 25 | 45.5 | 100 | Working memory task |  |  | X |  |
| SCZ | Ursu 2011 (Ursu et al., 2011) | 23 | 24 | 29.4 | 74 | 73.91 | Emotional processing | X |  |  |  |
| SCZ | Weiss 2003 (E. M. Weiss et al., 2003) | 13 | 13 | 32.7 | 100 | 100 | Stroop task |  |  | X |  |
| SCZ | Weiss 2006 (A. P. Weiss et al., 2006) | 16 | 16 | 46.9 | 87.5 | 100 | Verbal memory task |  |  | X |  |
| SCZ | White 2015 (White, Kraguljac, Reid, & Lahti, 2015) | 22 | 19 | 39.41 | 77.3 | 100 | Probabilistic reward decision task | X |  |  |  |
| SCZ | Broome 2009 (Broome et al., 2009) | 10 | 15 | 25.5 | 70 | 70 | Working memory + verbal fluency task |  |  | X |  |
| SCZ | Ciaramidaro 2017 (Ciaramidaro et al., 2018) | 20 | 25 | 24.7 | 70 | 40 | Emotional processing |  | X |  |  |
| SCZ | Fakra 2008 (Fakra, Salgado-Pineda, Delaveau, Hariri, & Blin, 2008) | 14 | 14 | 37.29 | 64.29 | 100 | Emotional face matching |  |  |  | X |
| SCZ | Kircher 2001 FTD (T. T. J. Kircher et al., 2001) | 6 | 7 | 34.3 | 100 | 100 | Sentence completion and semantic decision |  |  | X |  |
| SCZ | Kircher 2001 no-FTD (T. T. J. Kircher et al., 2001) | 6 | 7 | 31.2 | 100 | 100 | Sentence completion and semantic decision |  |  | X |  |
| SCZ | Kirschner 2016 (Kirschner et al., 2016) | 27 | 25 | 31.9 | 66.67 | 100 | Monetary incentive delay task | X |  |  |  |
| SCZ | Öngür 2006 (Öngür et al., 2006) | 15 | 15 | 39.7 | 73.33 | NR | Episodic memory task |  |  | X |  |
| SCZ | Richter 2015 (Richter et al., 2015) | 16 | 16 | 31.1 | 87.5 | 100 | Desire-reason dilemma paradigm | X |  | X |  |
| SCZ | Perlstein 2003 (Perlstein, Dixit, Carter, Noll, & Cohen, 2003) | 16 | 15 | 36.8 | 68.75 | 100 | n-back + AX continuous performance task |  |  | X |  |
| SCZ | Sapara 2014 with insight (Sapara et al., 2014) | 18 | 20 | 35.3 | 77.78 | NR | n-back task |  |  | X |  |
| SCZ | Sapara 2014 without insight (Sapara et al., 2014) | 14 | 20 | 37.7 | 64.29 | NR | n-back task |  |  | X |  |
| SCZ | Sass 2013 (Sass et al., 2014) | 14 | 14 | 36.4 | 57.14 | 100 | Lexical decision task |  |  | X |  |
| SCZ | Satterthwaite 2010 (Satterthwaite et al., 2010) | 16 | 21 | 37.6 | 60 | 87.5 | Emotional memory task | X | X | X |  |
| SCZ | Scheuerecker 2008 (J. Scheuerecker et al., 2008) | 23 | 23 | 31.6 | 82.61 | 0 | n-back task |  |  | X |  |
| SCZ | Schlagenhauf 2008 (Schlagenhauf et al., 2008) | 10 | 10 | 34.6 | 80 | 100 | n-back task |  |  | X |  |
| SCZ | Schlösser 2008 (Schlösser et al., 2008) | 41 | 41 | 30.17 | 68.29 | 100 | Sternberg task |  |  | X |  |
| SCZ | Seok Jeong 2005 (Seok Jeong et al., 2005) | 10 | 10 | 29.2 | 30 | 100 | Stroop task |  |  | X |  |
| SCZ | Shad 2012 (Shad et al., 2012) | 17 | 15 | 40 | 82.35 | NR | Self-other paradigm |  |  |  | X |
| SCZ | Siemerkus 2012 (Siemerkus, Irle, Schmidt-Samoa, Dechent, & Weniger, 2012) | 16 | 16 | 29.5 | 68.75 | 100 | Virtual maze |  |  | X |  |
| SCZ | Silverstein 2010 (S. M. Silverstein et al., 2010) | 14 | 13 | 32.62 | 64.29 | 92.86 | Visuospatial perception task |  |  | X | X |
| SCZ | Silverstein 2010 (Steven M. Silverstein et al., 2010) | 14 | 16 | 32.62 | 64.29 | 92.86 | Target detection |  |  | X |  |
| SCZ | Smee 2011 (Smee et al., 2011) | 9 | 9 | 36.8 | 88.89 | 100 | Verbal fluency task |  |  | X |  |
| SCZ | Stoltz 2012 (Stolz et al., 2012) | 22 | 28 | 28.35 | 50 | 100 | Episodic memory task |  |  | X |  |
| SCZ | Straube 2013 (Straube, Green, Sass, Kirner-Veselinovic, & Kircher, 2013) | 16 | 16 | 38 | 62.5 | 100 | Gesture-speech combination processing |  |  |  | X |
| SCZ | Sugranyes 2012 (Sugranyes et al., 2012) | 22 | 19 | 17.1 | 63.64 | 100 | n-back task |  |  | X |  |
| SCZ | Surguladze 2001 (Simon A Surguladze et al., 2001) | 14 | 7 | 35.9 | 71.43 | NR | Audiovisual speech perception, inner speech |  |  | X |  |
| SCZ | Tagamets 2014 (Tagamets, Cortes, Griego, & Elvevåg, 2014) | 11 | 11 | 40 | 81.82 | 100 | Word pairing |  |  | X |  |
| SCZ | Takahashi 2004 (Hidehiko Takahashi et al., 2004) | 15 | 15 | 29 | 66.67 | 73.33 | Emotional processing |  | X |  |  |
| SCZ | Takahashi 2008 (H. Takahashi et al., 2010) | 12 | 12 | 31.8 | 50 | 100 | Motion perception |  |  | X |  |
| SCZ | Taylor 2011 (Taylor, Chen, Tso, Liberzon, & Welsh, 2011) | 21 | 21 | 40.7 | 66.67 | 100 | Social judgement task |  |  |  | X |
| SCZ | Tendolkar 2004 (Tendolkar et al., 2004) | 12 | 12 | NR | 100 | 100 | Semantic decision task |  |  | X |  |
| SCZ | Tregellas 2012 (Tregellas, Smucny, Eichman, & Rojas, 2012) | 22 | 17 | 37.6 | 81.82 | 95.45 | Auditory oddball task |  |  | X |  |
| SCZ | Van Der Meer 2013 (L. Van Der Meer et al., 2013) | 47 | 21 | 34.3 | 75 | 95.74 | Self-other paradigm |  |  |  | X |
| SCZ | Walter 2009 (Walter, Ciaramidaro, et al., 2009) | 12 | 12 | 29.5 | 50 | 100 | Theory of mind |  |  |  | X |
| SCZ | Waltz 2008 (Waltz et al., 2009) | 18 | 18 | 37.7 | 72.22 | 100 | Classical conditioning paradigm | X |  |  |  |
| SCZ | Weiss 2007 (E. M. Weiss et al., 2007) | 8 | 8 | 29.5 | 100 | 0 | Stroop task |  |  | X |  |
| SCZ | Weiss 2009 (A. P. Weiss et al., 2009) | 18 | 18 | 42.5 | 66.67 | 100 | Recognition memory paradigm |  |  | X |  |
| SCZ | Wilmsmeier 2010 (Wilmsmeier et al., 2010) | 36 | 28 | 27.6 | 55.56 | 100 | Wisconsin card sorting |  |  | X |  |
| SCZ | Woodward 2009 (Woodward et al., 2009) | 25 | 32 | 26.9 | 80 | 40 | Choice-reaction |  |  | X |  |
| SCZ | Yoon 2013 (J. H. Yoon, Minzenberg, Raouf, D’Esposito, & Carter, 2013) | 18 | 19 | 33.1 | 66.7 | 100 | Working memory task |  |  | X |  |
| SCZ | Zedkova 2006 (Zedkova, Woodward, Harding, Tibbo, & Purdon, 2006) | 10 | 15 | 33.5 | 80 | 100 | Serial reaction time task |  |  | X |  |
| SCZ | Zierhut 2010 (Zierhut et al., 2010) | 11 | 11 | 29 | 63.64 | 100 | Episodic memory task |  |  | X |  |
| SCZ | Lee 2006 (K.-H. Lee et al., 2006) | 14 | 14 | 31.7 | 92.86 | 100 | Empathic judgements |  |  |  | X |
| SCZ | Lee 2008 (J. Lee, Folley, Gore, & Park, 2008) | 8 | 7 | 34.9 | 62.5 | 100 | Working memory task |  |  | X |  |
| SCZ | Lee 2011 (J. Lee, Quintana, Nori, & Green, 2011) | 12 | 13 | 38.3 | 83.33 | 100 | Theory of mind |  |  |  | X |
| SCZ | Leitman 2011 (Leitman et al., 2011) | 24 | 28 | 34.1 | 65.22 | NR | Emotion recognition | X | X |  | X |
| SCZ | Lencer 2005 (Lencer, Nagel, Sprenger, Heide, & Binkofski, 2005) | 17 | 16 | 35.5 | 100 | 94.12 | Smooth pursuit |  |  | X |  |
| SCZ | Leube 2003 (Leube et al., 2003) | 10 | 10 | NR | 60 | NR | Episodic memory task |  |  | X |  |
| SCZ | Leube 2010 (Leube, Knoblich, Erb, Schlotterbeck, & Kircher, 2010) | 10 | 10 | 34.2 | 50 | 100 | Perception of movement synchronization |  |  |  |  |
| SCZ | Linnman 2013 (Linnman, Coombs, Goff, & Holt, 2013) | 15 | 13 | 32 | 100 | 60 | Classical conditioning paradigm (shock) |  | X |  |  |
| SCZ | Luck 2009 (Luck et al., 2009) | 17 | 17 | 29.71 | NR | 100 | Working memory task |  |  | X |  |
| SCZ | Maïza 2010 (Maïza, 2010) | 10 | 10 | 34.2 | 80 | 100 | Story comprehension |  |  | X |  |
| SCZ | Martinez 2011 (Martinez et al., 2012) | 35 | 29 | 38.8 | 94.29 | 100 | Attention |  |  | X |  |
| SCZ | Matsuo 2013 (Kayako Matsuo et al., 2013) | 46 | 46 | 31.5 | 50 | NR | Sternberg task |  |  | X |  |
| SCZ | McAllindon 2010 (McAllindon, Wilman, Purdon, & Tibbo, 2010) | 15 | 14 | 37.4 | 100 | 100 | 2-choice reaction time |  |  | X |  |
| SCZ | Mendrek 2012 (Mendrek, Bourque, Dubé, Lakis, & Champagne, 2012) | 17 | 15 | 32.86 | 0 | 100 | Emotional valence rating task |  | X |  |  |
| SCZ | Mier 2010 (D. Mier et al., 2010) | 16 | 16 | 34.25 | 68.75 | 100 | Theory of mind | X | X |  | X |
| SCZ | Mier 2014 (Daniela Mier et al., 2014) | 11 | 16 | 32.45 | 64 | 100 | Emotion recognition |  |  |  | X |
| SCZ | Morris 2012 (Morris et al., 2012) | 21 | 16 | 33 | 56.25 | 100 | Reward paradigm | X |  |  |  |
| SCZ | Mukherjee 2014 (Mukherjee et al., 2014a) | 20 | 24 | 37.5 | 60 | 100 | Approachability judgement task |  |  |  | X |
| SCZ | Muprhy 2010 (Murphy et al., 2010) | 11 | 10 | 26.7 | 63.64 | NR | Self-other paradigm |  |  |  | X |
| SCZ | Nagel 2012 (Nagel, Sprenger, Steinlechner, Binkofski, & Lencer, 2012) | 17 | 16 | 29.6 | NR | 100 | Pursuit eye tracking |  |  | X |  |
| SCZ | Ngan 2003 (Ngan et al., 2003) | 14 | 29 | 35.1 | 85.71 | 100 | Auditory oddball task |  |  | X |  |
| SCZ | Pae 2008 (Pae et al., 2008) | 12 | 11 | 27.8 | 57.14 | 100 | n-back task |  |  | X |  |
| SCZ | Papagni 2011 (Papagni et al., 2011) | 40 | 48 | 35.22 | 82.5 | 100 | Verbal fluency task |  |  | X |  |
| SCZ | Park 2009 (K.-M. Park et al., 2009) | 15 | 16 | NR | NR | 100 | Emotion recognition | X |  |  | X |
| SCZ | Payoux 2004 (Payoux et al., 2004) | 6 | 6 | 5.7 | 100 | 100 | Motor task |  |  | X |  |
| SCZ | Pedersen 2012 (Pedersen, Koelkebeck, et al., 2012) | 15 | 14 | 29 | 60 | 100 | Theory of mind |  |  |  | X |
| SCZ | Pedersen 2012 achievers (Pedersen, Wilmsmeier, et al., 2012) | 21 | 25 | 26.8 | 47.62 | 100 | Wisconsin card sorting task |  |  | X |  |
| SCZ | Pedersen 2012 learners (Pedersen, Wilmsmeier, et al., 2012) | 10 | 25 | 30.8 | 70 | 100 | Wisconsin card sorting task |  |  | X |  |
| SCZ | Pedersen 2012 non-retainers (Pedersen, Wilmsmeier, et al., 2012) | 5 | 25 | 24.8 | 60 | 100 | Wisconsin card sorting task |  |  | X |  |
| SCZ | Pomarol-Clotet 2008 (E. Pomarol-Clotet et al., 2008) | 32 | 32 | 41.56 | 65.63 | 100 | n-back task |  |  | X |  |
| SCZ | Potvin 2013 (Potvin et al., 2013) | 14 | 21 | 32.6 | 100 | 100 | Mental rotation |  |  | X |  |
| SCZ | Prata 2011 (Prata et al., 2011) | 44 | 53 | 35.04 | 81.82 | 86.36 | Verbal fluency task |  |  | X |  |
| SCZ | Ragland 2008 (J.D. Ragland et al., 2008) | 13 | 14 | 36.2 | 46.15 | 100 | Semantic fluency task |  |  | X |  |
| SCZ | Ragland 2012 (John D. Ragland et al., 2012) | 19 | 20 | 26.5 | 75 | 89.47 | Working memory task |  |  | X |  |
| SCZ | Rametti 2009 (Rametti et al., 2009) | 22 | 24 | 31.7 | 50 | 100 | Emotional word recognition |  |  | X |  |
| SCZ | Rapp 2013 (Rapp et al., 2013) | 15 | 15 | 28.1 | 0 | 100 | Irony processing |  |  |  | X |
| SCZ | Rasetti 2009 (Rasetti et al., 2009) | 34 | 20 | 36.7 | 73.53 | 100 | n-back task |  |  | X |  |
| SCZ | Reid 2010 (Reid et al., 2010) | 14 | 18 | 40.4 | 69.23 | 100 | Stroop task |  |  | X |  |
| SCZ | Reiss 2006 (Reiss et al., 2006) | 10 | 10 | 29.1 | 90 | 100 | Reaction time task |  |  | X |  |
| SCZ | Royer 2009 (Royer et al., 2009) | 19 | 12 | 33 | NR | 100 | Hayling task + n-back |  |  | X |  |
| SCZ | Russell 2000 (Russell et al., 2000) | 5 | 7 | 36 | 100 | 100 | Reading the mind in the eyes test |  |  |  | X |
| SCZ | Assaf 2006 (Assaf et al., 2006) | 16 | 16 | 38.6 | 75 | 93.75 | Semantic memory task |  |  | X |  |
| SCZ | Bertolino 2009 (Bertolino et al., 2009) | 46 | 90 | 28.1 | 81.32 | 100 | n-back task |  |  | X |  |
| SCZ | Bonner-Jackson 2005 (Bonner-Jackson, Haut, Csernansky, & Barch, 2005) | 17 | 26 | 21.8 | 88.2 | 94.12 | Episodic memory task |  |  | X |  |
| SCZ | Brüne 2008 (Brüne et al., 2008) | 9 | 13 | 27.89 | 33.33 | 100 | Theory of mind |  |  |  | X |
| SCZ | Callicott 2000 (J. H. Callicott, 2000) | 37 | 32 | 33.9 | 76.92 | NR | n-back task |  |  | X |  |
| SCZ | Dowd 2012 (Dowd & Barch, 2012) | 25 | 20 | 31.44 | 72 | 100 | Reward paradigm | X |  |  |  |
| SCZ | Ettinger 2011 (Ettinger et al., 2011) | 45 | 19 | 37.33 | 77.78 | 97.78 | n-back task |  |  | X |  |
| SCZ | Eyler 2008 (Eyler, Jeste, & Brown, 2008) | 17 | 14 | 47.2 | 88.24 | 100 | Verbal learning |  |  | X |  |
| SCZ | Ferri 2014 (Ferri et al., 2014) | 22 | 22 | 27.45 | 63.64 | 100 | Emotional processing | X | X |  |  |
| SCZ | Fu 2005 acute (Fu et al., 2005) | 9 | 11 | 34.6 | 100 | 100 | Verbal fluency task |  |  | X |  |
| SCZ | Fu 2005 remission (Fu et al., 2005) | 10 | 11 | 36 | 100 | 100 | Verbal fluency task |  |  | X |  |
| SCZ | Gradin 2013 (Gradin et al., 2013) | 14 | 18 | 42.71 | 85.71 | 100 | Reward paradigm | X |  |  |  |
| SCZ | Habel 2010 (Habel, Chechko, et al., 2010) | 17 | 17 | 34.4 | NR | 94.12 | Emotion recognition |  |  |  | X |
| SCZ | Hall 2008 (Jeremy Hall et al., 2008) | 24 | 24 | 37.7 | 63.16 | 100 | Emotional processing |  | X |  |  |
| SCZ | Hamilton 2009 (Hamilton et al., 2009) | 20 | 38 | 32.4 | 70 | 100 | Working memory task |  |  | X |  |
| SCZ | Hazlett 2008 (Hazlett et al., 2008) | 13 | 13 | 38.5 | 76.92 | 0 | Prepulse inhibition |  |  | X |  |
| SCZ | Holmes 2005 (Holmes et al., 2005) | 7 | 9 | 39 | 86 | 42.86 | Continuous performance task |  |  | X |  |
| SCZ | Hugdahl 2004 (Hugdahl et al., 2004) | 12 | 12 | 32.4 | 50 | 100 | Arithmetic working memory task |  |  | X |  |
| SCZ | Jamadar 2010 (S. Jamadar, Michie, & Karayanidis, 2010) | 11 | 11 | 36.75 | 72.73 | 100 | Switching task |  |  | X |  |
| SCZ | Kaladjian 2007 (Kaladjian et al., 2007) | 21 | 21 | 34.9 | 90.48 | 100 | Go/no-go task |  |  | X |  |
| SCZ | Kircher 2008 (T. Kircher, Whitney, Krings, Huber, & Weis, 2008) | 12 | 12 | 26.83 | 100 | 100 | Verbal fluency task |  |  | X |  |
| SCZ | Kosaka 2002 (Kosaka et al., 2002) | 12 | 12 | 26 | 50 | 100 | Emotional processing | X |  |  |  |
| SCZ | Kubicki 2003 (Kubicki, 2003) | 9 | 9 | 39.7 | 100 | NR | Semantic memory task |  |  | X |  |
| SCZ | Laurens 2003 (K. R. Laurens, 2003) | 10 | 16 | 32.9 | 90 | 100 | Go/no-go task |  |  | X |  |
| SCZ | Laurens 2005 (Kristin R. Laurens, Kiehl, Ngan, & Liddle, 2005) | 28 | 28 | 31.6 | 68.97 | 100 | Oddball task |  |  | X |  |
| SCZ | Lee 2014 (S.-K. Lee et al., 2014) | 15 | 14 | 31.7 | 53.33 | 100 | Emotional judgement | X |  |  | X |
| SCZ | Lepage 2006 (Martin Lepage et al., 2006) | 15 | 18 | 34 | 67 | 93.33 | Episodic memory task |  |  | X |  |
| SCZ | Lepage 2011 (M. Lepage et al., 2011) | 26 | 26 | 31.8 | 58 | 88.46 | Emotional processing | X | X |  |  |
| SCZ | Li 2012 (H. Li et al., 2012) | 12 | 12 | 29.8 | 50 | NR | Emotional processing | X |  |  |  |
| SCZ | Liddle 2006 (Liddle, Laurens, Kiehl, & Ngan, 2006) | 28 | 28 | 31.6 | 67.86 | 100 | Auditory oddball task |  |  | X |  |
| SCZ | Meisenzahl 2006 (Meisenzahl et al., 2006) | 12 | 12 | 33.5 | 91.67 | 0 | n-back task |  |  | X |  |
| SCZ | Michalopoulou 2008 (Michalopoulou et al., 2008) | 11 | 9 | 35 | 81.82 | 100 | Emotional processing |  | X |  |  |
| SCZ | Mothersill 2014 (Mothersill et al., 2014) | 25 | 21 | 42.88 | 80 | NR | Emotional processing |  | X |  |  |
| SCZ | Ragland 2005 (J. Daniel Ragland et al., 2005) | 14 | 14 | 35.1 | 85.71 | 100 | Episodic memory task |  |  | X |  |
| SCZ | Rowland 2010 (Rowland, Griego, Spieker, Cortes, & Holcomb, 2010) | 17 | 17 | 41.9 | 52.94 | 100 | Episodic memory task |  |  | X |  |
| SCZ | Rubia 2001 (Rubia et al., 2001) | 6 | 7 | 40 | 100 | 100 | Go/no-go task |  |  | X |  |
| SCZ | Schlagenhauf 2014 (Schlagenhauf et al., 2014) | 22 | 24 | 27.5 | 91.67 | 0 | Reversal learning task | X |  |  |  |
| SCZ | Sergerie 2010 (Sergerie, Armony, Menear, Sutton, & Lepage, 2010) | 30 | 25 | 31.8 | 55 | 100 | Emotional memory task | X |  | X |  |
| SCZ | Shin 2015 (Shin et al., 2015) | 16 | 16 | 32 | 100 | 100 | Emotional processing |  | X |  |  |
| SCZ | Spilka 2015 (Spilka, Arnold, & Goghari, 2015) | 28 | 27 | 41.07 | 53.57 | 100 | Emotional processing |  | X |  |  |
| SCZ | Ungar 2010 (Ungar, Nestor, Niznikiewicz, Wible, & Kubicki, 2010) | 15 | 15 | 43 | 100 | 100 | Stroop task |  |  | X |  |
| SCZ | Walter 2007 (Walter, Vasic, Höse, Spitzer, & Wolf, 2007) | 19 | 17 | 33.1 | 66.67 | 100 | Working memory task |  |  | X |  |
| SCZ | Williams 2007 paranoid (Williams et al., 2007) | 13 | 22 | 26.9 | 61.54 | 100 | Emotional processing |  | X |  |  |
| SCZ | Williams 2007 nonparanoid (Williams et al., 2007) | 13 | 22 | 27.8 | 64.29 | 100 | Emotional processing |  | X |  |  |
| SCZ | Yoo 2005 (Yoo et al., 2005) | 10 | 10 | 24.9 | 66.67 | 100 | n-back task |  |  | X |  |
| SCZ | Gur 2007 (Gur et al., 2007) | 22 | 28 | 30.5 | 59.09 | 95.45 | Oddball task |  |  | X |  |
| SCZ | Adamczyk 2017 (Adamczyk et al., 2017) | 20 | 20 | 40 | 50 | 96,00 | Humor comprehension | X |  |  | X |
| SCZ | Arnold 2016 (Arnold, Iaria, & Goghari, 2016) | 27 | 24 | 41 | 48.15 | NR | Functional localizer task |  |  |  | X |
| SCZ | Arrondo 2015 (Arrondo et al., 2015) | 22 | 21 | 33 | 13.64 | 100 | Monetary incentive delay | X |  |  |  |
| SCZ | Bergé 2014 (Bergé et al., 2014) | 18 | 19 | 25 | 44.40 | 0 | Emotional processing | X | X |  | X |
| SCZ | Brandt 2014 (Brandt et al., 2014) | 100 | 100 | 32 | 33.00 | 76 | n-back task |  |  | X |  |
| SCZ | Chen 2016 (X. Chen et al., 2016) | 22 | 25 | 33 | 36.36 | 100 | Prospective memory task |  |  | X |  |
| SCZ | Collier 2014b (Collier et al., 2014) | 16 | 21 | NR | 45.00 | 100 | Oddball task |  |  | X |  |
| SCZ | Dowd 2016 (Dowd, Frank, Collins, Gold, & Barch, 2016) | 37 | 38 | 35 | 36.80 | 92.11 | Probabilistic stimulus selection task |  |  | X |  |
| SCZ | Eryilmaz 2016 (Eryilmaz et al., 2016) | 40 | 40 | 42 | 22.50 | 72 | Working memory |  |  | X |  |
| SCZ | Fassbender 2014 (Fassbender, Scangos, Lesh, & Carter, 2014) | 25 | 26 | 20 | 28.00 | 64 | Stroop task |  |  | X |  |
| SCZ | Francis 2016 (Francis et al., 2016) | 35 | 20 | 23 | 25.71 | 100 | Episodic memory |  |  | X |  |
| SCZ | Gaebler 2015 (Gaebler et al., 2015) | 24 | 24 | 36 | 41.67 | 95.83 | Auditory mismatch task |  |  | X |  |
| SCZ | Garrisson 2017 (Garrison, Fernandez-Egea, Zaman, Agius, & Simons, 2017) | 20 | 20 | 36 | 10.00 | 100 | Reality monitoring |  |  | X |  |
| SCZ | Genzel 2015 (Genzel et al., 2015) | 16 | 16 | 39 | 50.00 | 100 | Finger-tapping task |  |  | X |  |
| SCZ | Grot 2017 (Grot et al., 2017) | 19 | 23 | 36 | 26.32 | 100 | Working memory task |  |  | X |  |
| SCZ | Guimond 2016 (Guimond et al., 2016) | 18 | 19 | 24 | 39.00 | 100 | Episodic memory task |  |  | X |  |
| SCZ | Guimond 2017 (Guimond, Hawco, & Lepage, 2017) | 35 | 23 | 33 | 14.29 | 100 | Semantic encoding memory task |  |  | X |  |
| SCZ | Harvey 2014 (Harvey & Lepage, 2014) | 28 | 26 | 31 | 39.29 | 89.29 | Picture recognition task |  |  | X | X |
| SCZ | Hawco 2015 (Hawco et al., 2015) | 24 | 23 | 24 | 15.38 | 0 | Source memory task |  |  | X | X |
| SCZ | Horan 2016 (Horan et al., 2016) | 21 | 21 | 48 | 28.60 | 100 | Empathy task |  | X |  | X |
| SCZ | Huang 2016 (J. Huang et al., 2016) | 23 | 23 | 34 | 13.04 | 100 | Effort-expenditure for reward task | X |  |  |  |
| SCZ | Jiang 2015 (Jiang et al., 2015) | 20 | 20 | 23 | 35.00 | 100 | n-back task |  |  | X |  |
| SCZ | Karpouzian 2017 (Karpouzian et al., 2017) | 29 | 23 | 33 | 41.38 | NR | Facial affect perception | X | X |  | X |
| SCZ | Keedy 2015 (Keedy, Reilly, Bishop, Weiden, & Sweeney, 2015) | 21 | 21 | 24 | 23.80 | 33.33 | Prosaccades |  |  | X |  |
| SCZ | Kim 2015b (G.-W. Kim, Yang, & Jeong, 2015) | 15 | 15 | 28 | 53.33 | 100 | Emotional task |  | X | X |  |
| SCZ | Krug 2014 (Krug et al., 2014) | 57 | 57 | 36 | 31.58 | NR | Decision-making |  |  | X |  |
| SCZ | landin-romero 2015 (Landin-Romero et al., 2015) | 28 | 56 | 36 | 33.33 | 100 | n-back |  |  | X |  |
| SCZ | Lee 2014 (H. Lee et al., 2014) | 15 | 16 | 31 | 33.00 | 100 | Handshake task |  | X |  | X |
| SCZ | Lee 2015b (J. S. Lee et al., 2015) | 20 | 20 | 37 | 50.00 | 100 | Reality evaluation |  |  | X |  |
| SCZ | Lee 2015c (K.-H. Lee et al., 2015) | 28 | 17 | 41 | 17.86 | NR | Go/no-go |  |  | X |  |
| SCZ | Lindner 2014 (Lindner et al., 2014) | 36 | 40 | 31 | 38.89 | 100 | Emotional task |  | X |  | X |
| SCZ | lopez-garcia 2016 (Lopez-Garcia et al., 2016) | 15 | 20 | 39 | 20.00 | 100 | Context processing |  |  | X |  |
| SCZ | Losak 2016 (Lošák et al., 2016) | 28 | 27 | 32 | 21.43 | 100 | Predictive motor timing paradigm |  |  | X |  |
| SCZ | Luck 2016 (Luck, Joober, Malla, & Lepage, 2016) | 24 | 20 | 25 | 20.83 | 87.50 | Visual memory task | X | X | X |  |
| SCZ | Madre 2014 (Madre et al., 2014) | 22 | 22 | 45 | 41.00 | 100 | n-back task |  |  | X |  |
| SCZ | Mukherjee 2014 (Mukherjee et al., 2014b) | 20 | 24 | 35 | 33.33 | 100 | Approachability judgement task | X | X |  | X |
| SCZ | Oh 2015 (Oh et al., 2015) | 16 | 16 | 30 | 50.00 | 100 | Emotion identification |  |  |  |  |
| SCZ | Okruszek 2018 (Okruszek et al., 2018b) | 25 | 26 | 36 | 48.00 | 96 | Judge if people are acting together |  |  |  | X |
| SCZ | Phillips 2015 (Phillips, Salo, & Carter, 2015) | 32 | 23 | 25 | 21.88 | 81.25 | AX continuous performance |  |  | X |  |
| SCZ | Poppe 2016 (Poppe et al., 2016) | 47 | 56 | 36 | 25.50 | 97.87 | Dot pattern expectancy task |  |  | X |  |
| SCZ | Ragland 2015b (J. Daniel Ragland et al., 2015) | 52 | 57 | 34 | 23.00 | 92.31 | Relational encoding and retrieval |  |  | X |  |
| SCZ | Ragland 2017 (J.D. Ragland et al., 2017) | 24 | 26 | 25 | 25.00 | 100 | Spatial memory task |  |  | X |  |
| SCZ | Rasetti 2014 (Rasetti et al., 2014) | 62 | 181 | 34 | 30.65 | 100 | Declarative memory paradigm |  |  | X |  |
| SCZ | Razafimandimby 2016 (Razafimandimby et al., 2016) | 21 | 25 | 34 | 23.80 | 100 | Emotional task | X | X |  |  |
| SCZ | Reinen 2016 (Reinen et al., 2016) | 16 | 23 | 34 | 43.75 | 0 | Probabilistic learning task | X | X |  |  |
| SCZ | Schnell 2016 (Schnell et al., 2016) | 24 | 21 | NR | 57.14 | NR | Irony comprehension |  |  |  | X |
| SCZ | Shergill 2014 (Shergill et al., 2014) | 19 | 19 | 36 | 21.05 | 100 | Sensorimotor task |  |  | X |  |
| SCZ | Singh 2015 (S. Singh et al., 2015) | 14 | 14 | 32 | 21.40 | NR | Emotional task |  | X |  | X |
| SCZ | Smieskova 2015 (Smieskova et al., 2015) | 29 | 19 | 26 | 25.64 | 41.38 | Cognitive empathy | X | X |  | X |
| SCZ | Smith 2015 (Smith et al., 2015) | 30 | 24 | 34 | 40.00 | NR | Salience attribution task | X | X |  | X |
| SCZ | Smucny 2018 (Smucny et al., 2018b) | 70 | 53 | 21 | 15.71 | 80.00 | AX continuous performance task |  |  | X |  |
| SCZ | Spaniel 2016 (Spaniel et al., 2016) | 35 | 35 | 29 | 48.60 | 100 | Motor task |  |  | X |  |
| SCZ | Spilka 2017 (Spilka & Goghari, 2017) | 28 | 27 | 41 | 46.00 | 100 | Emotional task | X | X |  | X |
| SCZ | Tan 2015 (S. Tan et al., 2015) | 18 | 17 | 41 | 38.89 | 100 | Personality trait judgement task |  |  |  | X |
| SCZ | Thoresen 2014 (Thoresen et al., 2014) | 19 | 20 | 29 | 53.00 | 61.90 | Reality monitory |  |  | X |  |
| SCZ | Tikasz 2016 (Tikàsz et al., 2016) | 20 | 10 | 30 | 0 | 100 | Emotional task |  | X |  | X |
| SCZ | Tseng 2016 (H.-H. Tseng et al., 2016) | 18 | 21 | 27 | 27.78 | 55.56 | Emotional task | X | X |  | X |
| SCZ | van meer 2014 (Lisette Van Der Meer et al., 2014) | 20 | 20 | 35 | 20.00 | 100 | Emotion regulation task |  | X |  |  |
| SCZ | Vistolli 2017 (Vistoli, Lavoie, Sutliff, Jackson, & Achim, 2017) | 27 | 21 | 30 | 14.80 | 100 | Emotional task |  | X |  |  |
| SCZ | Wende 2015 (Wende et al., 2015) | 18 | 18 | 36 | 11.11 | 100 | Judgement of causality |  |  |  | X |
| SCZ | Wojtalik 2014 (Wojtalik & Barch, 2014) | 20 | 20 | 36 | 45.00 | 100 | n-back |  |  | X |  |
| SCZ | Zheng 2016 (Zheng et al., 2016) | 20 | 16 | 32 | 45.00 | 100 | Speech recognition |  |  | X |  |
| MDD | Admon 2015 (Admon et al., 2015) | 33 | 35 | 47.4 | 48.48 | 39.4 | Psychological stress task |  | X |  |  |
| MDD | Arrondo 2015 (Arrondo et al., 2015) | 24 | 21 | 33.1 | 70.83 | 54.17 | Monetary incentive delay task | X |  |  |  |
| MDD | Aust 2013 (Aust, 2013) | 14 | 14 | 55.1 | NR | NR | Mood induction | X |  |  |  |
| MDD | Backes 2014 (H. Backes et al., 2014) | 33 | 33 | 35.8 | 39.39 | 87.88 | Semantic word generation |  |  | X |  |
| MDD | Bermpohl 2009 (Bermpohl et al., 2009) | 15 | 21 | 43.4 | 20 | 93.33 | Emotional processing | X | X |  |  |
| MDD | Briceno 2013 (Briceño et al., 2013) | 24 | 22 | 37.8 | 0 | 58.33 | Emotional processing | X | X |  | X |
| MDD | Cerullo 2014 (Cerullo et al., 2014) | 25 | 25 | 27 | 32 | 0 | Continuous performance task | X | X | X |  |
| MDD | Chandrasekhar 2015 (Chandrasekhar Pammi et al., 2015) | 10 | 10 | 31.9 | 80 | NR | Reward paradigm | X |  |  |  |
| MDD | Chase 2013 (Chase et al., 2013) | 40 | 37 | 31 | 22.5 | NR | Reward paradigm | X |  |  |  |
| MDD | Chechko 2013 (Chechko et al., 2013) | 18 | 18 | 36.5 | 27.78 | 66.67 | Emotional judgement | X | X | X | X |
| MDD | Demenescu 2011 (Demenescu et al., 2011) | 59 | 56 | 36.2 | 33.9 | 23.7 | Emotional processing | X |  |  |  |
| MDD | Derntl 2011 (Derntl et al., 2011) | 15 | 15 | 34.1 | 40 | 86.67 | Approach/avoidance paradigm | X |  |  | X |
| MDD | Dichter 2009 (Dichter, Felder, & Smoski, 2009) | 14 | 15 | 34.8 | 50 | 0 | Emotional oddball task |  | X |  |  |
| MDD | Dichter 2012 (Dichter, Kozink, McClernon, & Smoski, 2012) | 19 | 19 | 24.5 | 21.05 | 89.47 | Monetary incentive delay task | X |  |  |  |
| MDD | Dietsche 2014 (Dietsche et al., 2014) | 23 | 23 | 36.7 | 39.13 | 86.96 | Episodic memory task |  |  | X |  |
| MDD | Epstein 2006 (Epstein et al., 2006) | 10 | 12 | 35.6 | 10 | 0 | Emotional processing | X |  |  |  |
| MDD | Fitzgerald 2008 (Fitzgerald et al., 2008) | 13 | 13 | 38.4 | 61.54 | 84.62 | Tower of London + n-back task |  |  | X |  |
| MDD | Fournier 2013 (Fournier et al., 2013) | 26 | 28 | 30.6 | 30.77 | 69 | Emotional processing | X | X |  |  |
| MDD | Frodl 2009 (Frodl et al., 2009) | 12 | 12 | 43.3 | 41.67 | 66.67 | Emotional face matching | X | X |  | X |
| MDD | Fu 2007 (Fu et al., 2007) | 19 | 19 | 43.2 | 31.58 | 0 | Emotional processing | X |  |  |  |
| MDD | Garrett 2011 psychotic (Garrett et al., 2011) | 16 | 19 | 34.1 | 56.25 | 87.5 | n-back task |  |  | X |  |
| MDD | Garrett 2011 non-psychotic (Garrett et al., 2011) | 15 | 19 | 39.8 | 33.33 | 60 | n-back task |  |  | X |  |
| MDD | Gotlib 2005 (Gotlib et al., 2005) | 18 | 18 | 35.2 | 27.78 | 50 | Emotional processing |  |  |  |  |
| MDD | Greening 2013 (Greening, Osuch, Williamson, & Mitchell, 2013) | 18 | 18 | 26.6 | 33.33 | 0 | Emotional processing |  | X |  |  |
| MDD | Greening 2014 (Greening, Osuch, Williamson, & Mitchell, 2014) | 19 | 19 | 26.8 | 31.58 | 0 | Emotion regulation | X |  |  |  |
| MDD | Grimm 2009 (Grimm et al., 2009) | 19 | 29 | 40 | 42.11 | 0 | Emotional processing | X | X |  | X |
| MDD | Hao 2015 (Hao et al., 2015) | 20 | 22 | 32.3 | 70 | NR | Empathy | X | X |  | X |
| MDD | Harvey 2005 (Harvey et al., 2005) | 10 | 10 | 33.8 | 30 | NR | n-back task |  |  | X |  |
| MDD | Heller 2009 (Heller et al., 2009) | 27 | 19 | 31.5 | 44.44 | 0 | Emotion regulation | X | X |  |  |
| MDD | Hugdahl 2007 (Hugdahl et al., 2007) | 9 | 12 | 36 | 44.44 | 100 | Arithmetic working memory task |  |  | X |  |
| MDD | Johnston 2015 (Johnston et al., 2015) | 19 | 21 | 50.8 | 21.05 | 100 | Reward paradigm |  | X |  |  |
| MDD | Johnstone 2007 (Johnstone, Van Reekum, Urry, Kalin, & Davidson, 2007) | 21 | 18 | 33 | 38.1 | 0 | Emotion regulation | X | X |  |  |
| MDD | Kassel 2016 (Kassel et al., 2016) | 40 | 42 | 43.3 | 52.5 | 40.5 | Semantic list learning task |  |  | X |  |
| MDD | Keedwell 2005 (Keedwell, Andrew, Williams, Brammer, & Phillips, 2005) | 12 | 12 | 43 | 33.33 | 91.67 | Mood induction | X |  | X |  |
| MDD | Langenecker 2007 (Langenecker et al., 2007) | 16 | 17 | 41 | 43.75 | 0 | Go/no-go task |  |  | X |  |
| MDD | Lisiecka 2013 (Lisiecka et al., 2013) | 50 | 25 | 42.7 | 34 | 74 | Emotional processing | X | X | X | X |
| MDD | Matsuo 2007 (K Matsuo et al., 2007) | 15 | 15 | 34.3 | 33.33 | 0 | n-back task |  |  | X |  |
| MDD | Matthews 2009 (Matthews et al., 2009) | 12 | 15 | 24.5 | NR | 0 | Stop-signal task |  |  | X |  |
| MDD | Mingtian 2012 (Mingtian et al., 2012) | 27 | 25 | 20.4 | 40.74 | 0 | Emotional face matching |  | X |  | X |
| MDD | Mitterschiffthaler 2008 (M. T. Mitterschiffthaler et al., 2008) | 17 | 17 | 39.3 | 17.65 | 0 | Stroop task |  | X | X |  |
| MDD | Murrough 2015 (Murrough et al., 2015) | 18 | 20 | 38.1 | 55.56 | 0 | Emotional valence rating task | X |  |  | X |
| MDD | Naismith 2010 (Naismith et al., 2010) | 19 | 20 | 56.1 | 26 | NR | Implicit learning motor sequencing paradigm |  |  | X |  |
| MDD | Norbury 2009 (Norbury, Selvaraj, Taylor, Harmer, & Cowen, 2010) | 16 | 21 | 36.2 | 43.75 | 0 | Emotional face matching task |  | X |  | X |
| MDD | Pizzagalli 2009 (Pizzagalli et al., 2009) | 26 | 31 | 43.2 | 57.69 | 0 | Monetary incentive delay task | X |  |  |  |
| MDD | Rao 2015 old (Rao et al., 2015) | 20 | 17 | 66.8 | NR | 78 | Go/no-go task |  |  | X |  |
| MDD | Rao 2015 young (Rao et al., 2015) | 16 | 18 | 26.4 | NR | 32 | Go/no-go task |  |  | X |  |
| MDD | Regenbogen 2015 (Regenbogen et al., 2015) | 24 | 24 | 36.4 | NR | NR | Empathy |  |  |  | X |
| MDD | Remijnse 2009 (Remijnse et al., 2009) | 20 | 27 | 35 | 60 | 0 | Reversal learning paradigm | X |  | X |  |
| MDD | Ritchey 2011 (Ritchey, Dolcos, Eddington, Strauman, & Cabeza, 2011) | 22 | 14 | 36.1 | 40.9 | 0 | Emotional valence rating task | X | X |  | X |
| MDD | Rizvi 2013 (Rizvi et al., 2013) | 21 | 18 | 38.9 | 33.33 | 0 | Emotional processing | X |  |  |  |
| MDD | Robinson 2012 (O. J. Robinson, Cools, Carlisi, Sahakian, & Drevets, 2012) | 13 | 14 | 36 | 61.54 | 0 | Reversal learning paradigm | X |  |  |  |
| MDD | Rodríguez-Cano 2014 (E. Rodríguez-Cano et al., 2014) | 26 | 52 | 46.5 | 38.46 | 92.31 | n-back task |  |  | X |  |
| MDD | Rose 2006 (Rose, Simonotto, & Ebmeier, 2006) | 9 | 9 | 32 | 22.22 | 88.89 | n-back task |  |  | X |  |
| MDD | Sarsam 2013 (Sarsam, Parkes, Roberts, Reid, & Kinderman, 2013) | 13 | 14 | 32.7 | 23.08 | 0 | Self-referential task |  |  |  | X |
| MDD | Scheuerecker 2010 (Johanna Scheuerecker et al., 2010) | 13 | 15 | 37.9 | 76.92 | 0 | Emotion recognition task |  | X |  | X |
| MDD | Schöning 2009 (Schöning et al., 2009) | 28 | 28 | 34.2 | 42.86 | 96.43 | n-back task |  |  | X |  |
| MDD | Segarra 2015 (Segarra et al., 2016) | 24 | 21 | 33.1 | 70.83 | 54.17 | Slot-machine game | X |  |  |  |
| MDD | Shi 2015 (Shi et al., 2015) | 29 | 33 | 20.5 | 37.93 | 0 | Gender discrimination task | X | X |  |  |
| MDD | Smoski 2009 (Smoski et al., 2009) | 14 | 15 | 34.8 | 50 | 0 | Wheel of fortune task | X |  | X |  |
| MDD | Smoski 2011 (Smoski, Rittenberg, & Dichter, 2011) | 9 | 13 | 34.4 | NR | 44.44 | Monetary incentive delay task | X |  |  |  |
| MDD | Surguladze 2005 (S. Surguladze et al., 2005) | 16 | 14 | 42.3 | 62.5 | 100 | Emotional processing | X |  |  |  |
| MDD | Surguladze 2010 (Simon A. Surguladze et al., 2010) | 9 | 9 | 42.8 | 55.56 | 100 | Emotional processing |  | X |  |  |
| MDD | Takamura 2016 (Takamura et al., 2016) | 16 | 16 | 39.4 | 62.5 | NR | Verbal fluency task |  |  | X |  |
| MDD | Townsend 2010 (Townsend et al., 2010) | 15 | 15 | 45.6 | 60 | 0 | Emotional face matching task | X | X |  | X |
| MDD | Tozzi 2016 (Tozzi et al., 2016) | 40 | 43 | NR | 32.5 | 75 | Mood induction | X | X |  | X |
| MDD | Tremblay 2005 (Tremblay et al., 2005) | 12 | 12 | 34.8 | 50 | 0 | Emotional valence rating task | X | X |  | X |
| MDD | Van Wingen 2011 (Van Wingen et al., 2011) | 18 | 30 | 33.3 | 38.89 | 0 | Emotional face matching task |  | X |  | X |
| MDD | Victor 2010 (Victor, Furey, Fromm, Öhman, & Drevets, 2010) | 22 | 25 | 31.1 | 45 | 0 | Emotional processing |  | X |  |  |
| MDD | Wagner 2015 (Wagner, Schachtzabel, Peikert, & Bär, 2015) | 19 | 20 | 39.9 | 42.11 | 100 | Self-referential task |  | X |  | X |
| MDD | Walsh 2007 (Walsh et al., 2007) | 20 | 20 | 43.7 | 30 | 0 | n-back task |  |  | X |  |
| MDD | Walter 2007 (Walter, Wolf, Spitzer, & Vasic, 2007) | 12 | 17 | 37.2 | 66.67 | 100 | Delayed match-to-sample task |  |  | X |  |
| MDD | Wang 2008 (L. Wang et al., 2008) | 19 | 20 | 39.3 | 36.84 | 57.89 | Emotional oddball task |  | X | X |  |
| MDD | Wang 2012 (Y. Wang et al., 2012) | 18 | 18 | 31.6 | 38.89 | 0 | Emotional processing | X |  |  |  |
| MDD | Werner 2009 (Werner et al., 2009) | 11 | 11 | 37.2 | 27.27 | 73 | Associative learning paradigm |  |  | X |  |
| MDD | Whalley 2012 (M. G. Whalley, Rugg, & Brewin, 2012) | 15 | 15 | 33.8 | 26.67 | 66.67 | Autobiographical memory task |  |  | X |  |
| MDD | Yang 2016 (X. Yang et al., 2016) | 25 | 25 | 29 | 48 | 0 | Reward paradigm | X |  |  |  |
| MDD | Young 2012 (K. D. Young et al., 2012) | 12 | 14 | 34 | 67 | 0 | Autobiographical memory task |  |  | X |  |
| MDD | Zhong 2011 (Zhong et al., 2011) | 29 | 31 | 20.5 | 44.83 | 0 | Emotional face matching task |  | X |  | X |
| MDD | Bär 2007 (Bär et al., 2007) | 13 | 13 | 35.9 | 0 | 0 | Thermal pain processing |  | X |  |  |
| MDD | Canli 2004 (Canli et al., 2004) | 15 | 15 | 35.1 | 20 | 46.67 | Emotional processing | X |  |  |  |
| MDD | Cooney 2010 (Cooney, Joormann, Eugène, Dennis, & Gotlib, 2010) | 14 | 14 | 40.6 | 42.86 | 42.86 | Rumination processing |  | X |  | X |
| MDD | Elliott 2002 (Elliott, Rubinsztein, Sahakian, & Dolan, 2002) | 10 | 11 | 42.2 | 30 | 100 | Emotional go/no-go task | X | X |  |  |
| MDD | Fu 2004 (Fu et al., 2004) | 19 | 19 | 43.2 | 31.58 | 0 | Emotion recognition |  | X |  | X |
| MDD | Kumari 2003 (Kumari et al., 2003) | 6 | 6 | 40 | 100 | 100 | Emotional processing |  | X |  |  |
| MDD | López-Solà 2010 (López-Solà et al., 2010) | 13 | 20 | 44.6 | 15.38 | 0 | Pain processing |  | X |  |  |
| MDD | Meusel 2013 (Meusel, Hall, Fougere, McKinnon, & MacQueen, 2013) | 35 | 15 | 48.4 | 20 | NR | n-back task + recollection memory task |  |  | X |  |
| MDD | Mitterschiffth 2003 (Martina T. Mitterschiffthaler et al., 2003) | 7 | 7 | 46.3 | 0 | 100 | Emotional processing | X |  |  |  |
| MDD | Rodríguez-Cano 2017 (Elena Rodríguez-Cano et al., 2017) | 26 | 26 | 46.5 | 38.46 | 100 | n-back task |  |  | X |  |
| MDD | Strigo 2008 (Irina A. Strigo, Simmons, Matthews, Craig, & Paulus, 2008) | 15 | 15 | 24.07 | 20 | 0 | Pain processing |  | X |  |  |
| MDD | Strigo 2013 (I A Strigo, Matthews, & Simmons, 2013) | 31 | 22 | 27.6 | 51.61 | 0 | Pain processing |  | X |  |  |
| MDD | Yang 2004 (J.-C. Yang, 2004) | 10 | 10 | 34.4 | 100 | 0 | Erotic video processing | X |  |  |  |
| MDD | Yang 2009 (T. T. Yang et al., 2009) | 13 | 13 | 16 | 46.15 | 0 | Stop-signal task |  |  | X |  |
| MDD | Holmes 2005 (Holmes et al., 2005) | 10 | 9 | 32 | 70 | NR | Continuous performance test |  |  | X |  |
| MDD | Lee 2013 (T.-W. Lee, Liu, Wai, Ko, & Lee, 2013) | 14 | 14 | 65.1 | 78.57 | 100 | n-back task |  |  | X |  |
| MDD | Wagner 2010 (Wagner et al., 2010) | 20 | 20 | 39.88 | 10 | 0 | Stroop task |  |  | X |  |
| MDD | Young 2016 (Kymberly D Young, Bodurka, & Drevets, 2016) | 30 | 30 | 37.1 | 12.5 | 0 | Autobiographical memory task | X |  | X |  |
| MDD | Ai 2018 (Ai et al., 2018) | 103 | 26 | 37.2 | 60.94 | 35 | Executive planning |  |  | X |  |
| MDD | Alders2019 (Alders et al., 2019) | 48 | 30 | 37.2 | 60.94 | 0 | Emotional Stroop task |  |  | X | X |
| MDD | Breukelaar 2019 (Breukelaar et al., 2020) | 25 | 25 | 34.7 | 31 | 100 | n-back task |  |  | X |  |
| MDD | Burrows 2021 high C-reactive (Burrows et al., 2021) | 44 | 44 | 34.8 | 47 | 31.8 | Monetary incentive delay task | X |  |  |  |
| MDD | Burrows 2021 low C-reactive (Burrows et al., 2021) | 44 | 44 | 34.5 | 27.27 | 27.3 | Monetary incentive delay task | X |  |  |  |
| MDD | Dai 2018 (Dai, Yin, Li, & Feng, 2018) | 30 | 33 | 48.8 | 23.33 | 100 | Emotional processing | X | X | X |  |
| MDD | Davis 2018 (Davis, Foland-Ross, & Gotlib, 2018) | 16 | 20 | 33.7 | 0 | 0 | Emotion regulation |  |  |  |  |
| MDD | De La Peña-Arteaga 2021 (De La Peña-Arteaga et al., 2021) | 20 | 19 | 49.8 | 30 | NR | Emotion regulation |  |  |  |  |
| MDD | DeVille 2018 (DeVille et al., 2018) | 24 | 21 | 29.3 | 37.5 | 0 | Interoceptive encoding and recall |  |  | X |  |
| MDD | Dong 2022 with childhood maltreatment (Dong et al., 2022) | 44 | 43 | 25.9 | 0 | 0 | Montreal imaging stress task |  | X |  |  |
| MDD | Dong 2022 without childhood maltreatment (Dong et al., 2022) | 32 | 94 | 25.4 | 0 | 0 | Montreal imaging stress task |  | X |  |  |
| MDD | Finlayson-Short 2021 (Finlayson-Short, Harrison, & Davey, 2021) | 17 | 98 | 19.8 | 47.1 | 0 | Self-other referential processing |  |  |  | X |
| MDD | Foell 2021 (Foell et al., 2021) | 26 | 24 | 34.5 | 23 | NR | Gambling task | X |  |  |  |
| MDD | Gao 2021 (Gao et al., 2021) | 55 | 44 | 21.2 | 63.64 | 40 | Balloon analogue risk-taking task |  |  | X |  |
| MDD | Gätner 2018 (Gärtner et al., 2018) | 57 | 61 | 40.5 | 43.86 | 38.6 | Working memory task |  |  | X |  |
| MDD | Goodin 2019 (Goodin, Lamp, Hughes, Rossell, & Ciorciari, 2019) | 13 | 14 | 30.6 | 23.08 | 69.23 | Working memory task |  | X | X |  |
| MDD | Groves 2018 (Groves et al., 2018) | 16 | 10 | 35.4 | 37 | 0 | Emotional processing |  | X |  | X |
| MDD | Huang 2019b (C.-M. Huang et al., 2019) | 55 | 40 | 66.4 | 30.91 | NR | Emotional Stroop task | X | X | X |  |
| MDD | Insel 2019 (Insel, Glenn, Nock, & Somerville, 2019) | 56 | 26 | 17.6 | 0 | 67.9 | Reward paradigm | X |  |  |  |
| MDD | Katayama 2019 (Katayama et al., 2019) | 23 | 23 | 36.7 | 30.43 | 86.96 | Future-thinking |  |  | X |  |
| MDD | Langenecker 2018 (Langenecker et al., 2018) | 21 | 39 | 21 | 33.33 | 0 | Go/no-go task |  |  | X |  |
| MDD | Lemke 2021 (Lemke et al., 2022) | 333 | 333 | 36.8 | 41.44 | 84.98 | Emotional face matching task |  | X |  | X |
| MDD | Li 2022 (Liyuan Li et al., 2022) | 37 | 37 | 31.2 | 32.43 | NR | Emotional processing | X | X |  |  |
| MDD | Loeffler 2018 (Loeffler et al., 2018) | 26 | 26 | 35.3 | 46.15 | NR | Emotion regulation |  |  |  | X |
| MDD | Malejko 2021 (Malejko et al., 2021) | 16 | 17 | 28.7 | 0 | 100 | Eriksen flanker, Go/no-go task |  |  | X |  |
| MDD | Nagy 2021 (Nagy et al., 2021) | 21 | 21 | 35.5 | 33.33 | 100 | Emotional face matching task |  |  |  | X |
| MDD | Nichols 2021 (Nichols et al., 2021) | 73 | 37 | NR | NR | 40.54 | Emotion regulation |  |  |  |  |
| MDD | Park 2022 very high repetitive negative thinking (H. Park et al., 2022) | 55 | 29 | 34.4 | 14.55 | 65.45 | Reward paradigm | X |  |  |  |
| MDD | Park 2022 high repetitive negative thinking (H. Park et al., 2022) | 52 | 29 | 35.3 | 26.92 | 65.45 | Reward paradigm | X |  |  |  |
| MDD | Parlar 2018 (Parlar, Densmore, Hall, Lanius, & McKinnon, 2018) | 20 | 20 | 40.1 | 50 | NR | Autobiographical memory task |  |  | X |  |
| MDD | Quevedo 2018 (Quevedo et al., 2018) | 43 | 38 | 14.7 | 48.84 | 51.16 | Self-other face recognition task | X | X |  | X |
| MDD | Rai 2021 (Rai et al., 2021) | 33 | 37 | 36.7 | 48.7 | 21 | Emotion regulation | X | X |  |  |
| MDD | Reinen 2021 (Reinen et al., 2021) | 23 | 24 | 26.6 | 52.17 | 0 | Reward paradigm | X |  |  |  |
| MDD | Rütgen 2019 (Rütgen et al., 2019) | 29 | 35 | 29.6 | 27.59 | 0 | Empathy |  |  |  | X |
| MDD | Trettin 2022 (Trettin et al., 2022) | 36 | 30 | 40.7 | 41.67 | 100 | Emotional processing |  | X |  |  |
| MDD | Van Kleef 2022 (Van Kleef et al., 2022) | 46 | 24 | 35.1 | 27.78 | 0 | Emotion regulation |  |  |  |  |
| MDD | Wang 2022 (X. Wang et al., 2022) | 18 | 18 | 42.1 | 27.78 | 77.78 | Self-other attribution |  |  |  | X |
| MDD | Yoon 2022 (L. Yoon et al., 2022) | 45 | 43 | 15.9 | 51.11 | NR | Gambling, reward paradigm | X |  |  |  |
| MDD | Yüksel 2018 (Yüksel et al., 2018) | 74 | 74 | 36.8 | 44.59 | 90.54 | n-back task |  |  | X |  |
| MDD | Zweerings 2019 (Zweerings et al., 2019) | 25 | 25 | 37.9 | 68 | 92 | Auditory mismatch paradigm |  |  | X |  |
| MDD | Willinger 2022 (Willinger et al., 2022) | 30 | 33 | 16.1 | 33.33 | 66.67 | Monetary incentive delay task | X |  |  |  |
| BD | Allin 2010 (Allin et al., 2010) | 18 | 19 | 39.2 | 38.89 | 72.22 | Verbal fluency task |  |  | X |  |
| BD | Alonso-Lana 2016 (S. Alonso-Lana et al., 2016) | 20 | 40 | 41 | 25 | 100 | n-back task |  |  | X |  |
| BD | Alonso-Lana 2016 (Silvia Alonso-Lana et al., 2016) | 27 | 28 | 44.5 | 55.55 | 100 | n-back task |  |  | X |  |
| BD | Altshuler 2005 (L. L. Altshuler et al., 2005) | 11 | 13 | 36 | 36 | 63.64 | Go/no-go task |  |  | X |  |
| BD | Altshuler 2008 (L. Altshuler et al., 2008) | 11 | 13 | 32 | 45.45 | 81.82 | Emotional face matching task |  | X |  | X |
| BD | Brooks 2015 (Brooks et al., 2015) | 19 | 19 | 36.7 | 57.89 | 0 | n-back task |  |  | X |  |
| BD | Caseras 2013 (Caseras, Lawrence, Murphy, Wise, & Phillips, 2013) | 15 | 19 | 41.7 | 38.7 | 81.25 | Reward paradigm | X |  |  |  |
| BD | Cerullo 2014 (Cerullo et al., 2014) | 25 | 25 | 30 | 32 | 92 | Emotional continuous performance task | X | X | X |  |
| BD | Chase 2013 (Chase et al., 2013) | 23 | 37 | 33.9 | 17.39 | NR | Reward paradigm | X |  |  |  |
| BD | Chen 2006 manic (C.-H. Chen et al., 2006) | 8 | 8 | 39 | 100 | 100 | Emotion recognition |  | X |  | X |
| BD | Chen 2006 depressive (C.-H. Chen et al., 2006) | 8 | 8 | 41.9 | 62.5 | 100 | Emotion recognition | X | X |  | X |
| BD | Costafreda 2011 (Costafreda et al., 2011) | 32 | 40 | 41.4 | 43.75 | 81.25 | Verbal fluency task |  |  | X |  |
| BD | Dima 2016 (Dima, De Jong, Breen, & Frangou, 2016) | 41 | 46 | 44.3 | 48.78 | NR | Emotion recognition | X | X |  | X |
| BD | Dutra 2015 (Dutra, Cunningham, Kober, & Gruber, 2015) | 24 | 25 | 31.4 | 37.5 | NR | Monetary incentive delay task | X |  |  |  |
| BD | Favre 2013 (Favre et al., 2013) | 16 | 16 | 40.4 | 43.75 | 93.75 | Emotional Stroop task | X | X | X | X |
| BD | Favre 2015 (Favre, Polosan, Pichat, Bougerol, & Baciu, 2015) | 14 | 13 | 44.1 | 42.86 | 85.71 | Emotional Stroop task | X | X | X | X |
| BD | Fernández-Corcuera 2013 (Fernández-Corcuera et al., 2013) | 41 | 41 | 40.4 | 56.09 | 100 | n-back task |  |  | X |  |
| BD | Fleck 2011 (Fleck et al., 2011) | 8 | 10 | 30 | 25 | 87.5 | Go/no-go task |  |  | X |  |
| BD | Foland-Ross 2012 (Foland-Ross et al., 2012) | 24 | 26 | 38.8 | 62.5 | 67 | Emotional face matching task | X | X |  | X |
| BD | Foland 2008 (Foland et al., 2008) | 9 | 9 | 34.6 | 33.33 | 77.78 | Emotion recognition, emotional face matching | X | X |  | X |
| BD | Glahn 2010 (Glahn et al., 2010) | 15 | 24 | 38 | 36 | 93.33 | Associative memory task |  |  | X |  |
| BD | Gruber 2009 (Gruber et al., 2009) | 18 | 18 | 38.2 | 55.6 | 83.33 | Delayed match-to-sample task |  |  | X |  |
| BD | Hamilton 2009 (Hamilton et al., 2009) | 21 | 38 | 36.4 | 61.9 | 80.95 | Working memory task |  |  | X |  |
| BD | Hassel 2008 (Hassel et al., 2008) | 19 | 24 | 32.5 | 52.63 | 94.74 | Emotional processing | X | X |  |  |
| BD | Hulvershorn 2012 (Hulvershorn et al., 2012) | 30 | 30 | 34 | 36.67 | 0 | Emotional face matching task |  | X |  | X |
| BD | Jamadar 2013 (Sharna Jamadar et al., 2013) | 32 | 133 | 36.7 | 46.88 | 65 | The semantic object retrieval task |  |  | X |  |
| BD | Jogia 2008 (Jogia, Haldane, Cobb, Kumari, & Frangou, 2008) | 8 | NR | 42.1 | 37.5 | 0 | Emotion recognition |  | X |  | X |
| BD | Jogia 2012 (Jogia, Dima, Kumari, & Frangou, 2012) | 36 | 37 | 42.5 | 47.22 | 61.11 | Iowa Gambling Task + n-back task | X |  | X |  |
| BD | Joshi 2016 (Joshi et al., 2016) | 45 | 45 | 39.9 | 53.33 | 76 | Go/no-go task |  |  | X |  |
| BD | Kaladjian 2009 (Kaladjian, Jeanningros, Azorin, Nazarian, Roth, & Mazzola-Pomietto, 2009) | 20 | 20 | 37.9 | 50 | 95 | Go/no-go task |  |  | X |  |
| BD | Kaladjian 2009 (Kaladjian, Jeanningros, Azorin, Nazarian, Roth, Anton, et al., 2009) | 10 | 10 | 40.1 | 50 | 100 | Go/no-go task |  |  | X |  |
| BD | Keener 2012 (Keener et al., 2012) | 27 | 27 | 32.4 | 37.04 | 96.3 | Emotional processing | X | X |  | X |
| BD | Killgore 2008 (Killgore, Gruber, & Yurgelun-Todd, 2008) | 14 | 13 | 28.1 | 78.57 | 100 | Emotional processing |  | X |  |  |
| BD | Kim 2009 (E. Kim et al., 2009) | 14 | 14 | 30.4 | 57.14 | 100 | Virtual reality social cognition task |  | X |  | X |
| BD | Kronhaus 2006 (Kronhaus et al., 2006) | 10 | 11 | 40.9 | 60 | 100 | Stroop task |  |  | X |  |
| BD | Lagopoulos 2007 (Lagopoulos, Ivanovski, & Malhi, 2007) | 10 | 10 | 32.4 | 0 | 70 | Working memory task |  |  | X |  |
| BD | Malhi 2005 (Malhi, Lagopoulos, Sachdev, Ivanovski, & Shnier, 2005) | 12 | 12 | 34.9 | 0 | 66.67 | Emotional Stroop task | X | X |  |  |
| BD | Malhi 2007 (Malhi, Lagopoulos, Sachdev, et al., 2007) | 10 | 10 | 33.5 | 0 | 70 | Emotion recognition |  | X |  | X |
| BD | Malhi 2007 (Malhi, Lagopoulos, Owen, et al., 2007) | 10 | 10 | 32.4 | 0 | 70 | Emotional working memory task | X |  | X |  |
| BD | Malhi 2008 (Malhi et al., 2008) | 20 | 20 | 35.3 | 55 | 65 | Theory of mind |  |  |  | X |
| BD | Marchand 2011 (Marchand et al., 2011) | 16 | 19 | 32.9 | 100 | 0 | Emotional processing | X |  |  |  |
| BD | Mazzola-Pomietto 2009 (Mazzola-Pomietto, Kaladjian, Azorin, Anton, & Jeanningros, 2009) | 16 | 16 | 35.8 | 37.5 | 100 | Go/no-go |  |  | X |  |
| BD | Mckenna 2013 (McKenna, Sutherland, Legenkaya, & Eyler, 2014) | 23 | 23 | 45.3 | 34.78 | 100 | Delayed match-to-sample task |  |  | X |  |
| BD | McIntosh 2008 (McIntosh et al., 2008) | 42 | 37 | 39.3 | 50 | NR | Imaginary sentence completion, inner speech |  |  | X |  |
| BD | Oertel-Knöchel 2013 (Oertel-Knöchel et al., 2013) | 26 | 25 | 37.3 | 50 | NR | Episodic memory task |  |  | X |  |
| BD | Oertel-Knöchel 2014 (Oertel-Knöchel et al., 2014) | 21 | 20 | 35.7 | 57.14 | 100 | Episodic memory task |  |  | X |  |
| BD | Penfold 2015 (Penfold, Vizueta, Townsend, Bookheimer, & Altshuler, 2015) | 19 | 20 | 36.3 | 47.37 | 0 | Go/no-go task |  |  | X |  |
| BD | Perlman 2012 (Perlman et al., 2012) | 31 | 25 | 32.6 | 32.26 | 100 | Emotional processing | X | X |  |  |
| BD | Pomarol-Clotet 2012 (Edith Pomarol-Clotet et al., 2012) | 29 | 46 | 40.8 | 62.07 | 100 | n-back task |  |  | X |  |
| BD | Pomarol-Clotet 2015 manic (Edith Pomarol-Clotet et al., 2015) | 38 | 38 | 39.7 | 47.37 | 100 | n-back task |  |  | X |  |
| BD | Pomarol-Clotet 2015 depressive (Edith Pomarol-Clotet et al., 2015) | 38 | 38 | 39.9 | 44.74 | 100 | n-back task |  |  | X |  |
| BD | Pomarol-Clotet 2015 euthymic (Edith Pomarol-Clotet et al., 2015) | 38 | 38 | 40 | 44.74 | 100 | n-back task |  |  | X |  |
| BD | Pompei 2011 (Pompei et al., 2011) | 39 | 48 | 39.4 | 48.71 | 76.92 | Stroop task |  |  | X |  |
| BD | Robinson 2009 (J. L. Robinson et al., 2009) | 15 | 15 | 39 | 47 | 93.33 | Delayed non-match-to-sample task |  |  | X |  |
| BD | Roth 2006 (Roth et al., 2006) | 11 | 11 | 37.7 | 63.64 | 100 | Stroop task |  |  | X |  |
| BD | Sepede 2012 (Sepede et al., 2012) | 24 | 24 | 34.8 | 41.67 | 83.3 | Continuous performance test |  |  | X |  |
| BD | Sepede 2015 (Sepede et al., 2015) | 23 | 24 | 35.2 | 39.13 | 82.6 | Emotional processing |  | X |  |  |
| BD | Strakowski 2005 (Strakowski et al., 2005) | 16 | 16 | 28 | 37.5 | 50 | Stroop task |  |  | X |  |
| BD | Surguladze 2010 (S.A. Surguladze et al., 2010) | 20 | 20 | 42.7 | 45 | 80 | Emotional processing | X | X |  |  |
| BD | Townsend 2012 (Townsend et al., 2012) | 32 | 30 | 37 | 65.63 | 72 | Go/no-go task |  |  | X |  |
| BD | Townsend 2013 (Townsend et al., 2013) | 30 | 26 | 37.9 | 63.33 | 70 | Emotion regulation |  | X |  |  |
| BD | Tseng 2016 (W.-L. Tseng et al., 2016) | 14 | 14 | 37.9 | 21.43 | 85.7 | Emotional processing |  | X |  |  |
| BD | Vizueta 2012 (Vizueta et al., 2012) | 21 | 21 | 38.4 | 52.38 | 0 | Emotional face matching task |  | X |  | X |
| BD | Wessa 2007 (Wessa et al., 2007) | 17 | 17 | 44.9 | 58.82 | 88.24 | Emotional go/no-go task | X | X | X |  |
| BD | Whalley 2009 (H. C. Whalley et al., 2009) | 14 | 14 | 41.5 | 64.29 | NR | Emotional memory paradigm | X | X |  |  |
| BD | Apazoglou 2019 (Apazoglou et al., 2019) | 20 | 20 | 33 | 50 | 100 | Introspection |  |  |  | X |
| BD | Breukelaar 2020 (Breukelaar et al., 2020) | 25 | 25 | 34.9 | 35 | 100 | n-back task |  |  | X |  |
| BD | Grant 2018 (Grant, Hassel, Bobyn, Hall, & MacQueen, 2018) | 25 | 25 | 35.4 | 48 | NR | Theory of mind |  | X |  | X |
| BD | Johnson 2019 (S. L. Johnson, Mehta, Ketter, Gotlib, & Knutson, 2019) | 24 | 24 | 37 | 50 | NR | Monetary incentive delay task | X |  |  |  |
| BD | King 2018 (King et al., 2018) | 35 | 35 | 15.7 | 42.86 | 71.43 | Motor task |  |  | X |  |
| BD | Lee 2019 (J. Lee et al., 2019) | 53 | 53 | 45.3 | 66.2 | 58.49 | Object discrimination task |  |  | X |  |
| BD | Li 2019 (Linling Li et al., 2019) | 13 | 16 | 29.6 | 23.08 | 100 | Emotional processing | X |  |  |  |
| BD | Lois 2020 (Lois, Schneider, Kaurin, & Wessa, 2020) | 41 | 41 | 44.6 | 95.24 | NR | Social decision-making |  | X |  | X |
| BD | Moser 2018 (Moser et al., 2018) | 37 | 48 | 27.5 | 67.57 | 81.1 | n-back task |  |  | X |  |
| BD | Negoias 2019 (Negoias et al., 2019) | 27 | 22 | 35.6 | 40.74 | 70.37 | Sniffin’ Sticks olfactory test | X | X |  |  |
| BD | Rai 2021 (Rai et al., 2021) | 38 | 37 | 39.2 | 40 | 28 | Emotion regulation |  |  |  |  |
| BD | Raucher-Chéné 2021 (Raucher‐Chéné et al., 2021) | 13 | 16 | 38.5 | NR | 84.6 | Semantic task |  |  | X |  |
| BD | Rauer 2021 (Rauer et al., 2021) | 20 | 40 | 39.3 | 40 | 75 | Oddball task |  |  | X |  |
| BD | Roybal 2022 (Roybal et al., 2022) | 19 | 14 | 15 | 52.63 | 53 | Cyberball social exclusion task |  |  |  | X |
| BD | Sepede 2020 (Sepede et al., 2020) | 26 | 26 | 37.3 | 62.5 | 100 | Continuous performance test |  |  | X |  |
| BD | Smucny 2018 (Smucny et al., 2018a) | 24 | 53 | 22.6 | 78.95 | 70.83 | AX version of the continuous performance task |  |  | X |  |
| BD | Xiao 2021 manic (Xiao et al., 2021) | 16 | 17 | 14.9 | 37.5 | NR | Emotional go/no-go | X | X | X | X |
| BD | Xiao 2021 euthymic (Xiao et al., 2021) | 18 | 17 | 15.2 | 50 | NR | Emotional go/no-go | X | X | X | X |
| BD | Zarp Petersen 2022 (Zarp Petersen et al., 2022) | 62 | 52 | 31 | 26 | 56 | n-back task |  |  | X |  |
| BD | Alonso‐Lana 2019 (Alonso‐Lana et al., 2019) | 26 | 26 | 39.19 | 57.69 | 100 | n-back task |  |  | X |  |
| BD | Chang 2004 (Chang et al., 2004) | 12 | 10 | 15.3 | 100 | 91.7 | n-back task |  |  | X |  |
| BD | Elliott 2004 (Elliott et al., 2004) | 8 | 11 | 33.5 | 50 | 87.5 | Emotional go/no-go |  |  | X |  |
| BD | Fernández-Corcuera 2013 (Fernández-Corcuera et al., 2013) | 41 | 41 | 40.39 | 56.1 | 100 | n-back task |  |  | X |  |
| BD | Goikolea 2019 (Goikolea et al., 2019) | 31 | 31 | 30.52 | 51.61 | 96.77 | n-back task |  |  | X |  |
| BD | Han 2018 (Han et al., 2018) | 10 | 10 | 38.6 | 60 | 100 | Pain processing | X |  |  |  |
| BD | Lagopoulos 2007 (Lagopoulos & Malhi, 2007) | 10 | 10 | 31.29 | 0 | 70 | Emotional Stroop task |  | X | X |  |
| BD | Lennox 2004 (Lennox, Jacob, Calder, Lupson, & Bullmore, 2004) | 10 | 12 | 37.3 | 80 | 100 | Emotional rating |  | X |  | X |
| BD | Monks 2004 (Monks et al., 2004) | 12 | 12 | 45.83 | 100 | 100 | n-back task |  |  | X |  |
| BD | Pavuluri 2010 (Pavuluri, Passarotti, Harral, & Sweeney, 2010) | 13 | 13 | 14.4 | 77 | 0 | Stop-signal task |  |  | X |  |
| BD | Rodríguez-Cano 2017 (Elena Rodríguez-Cano et al., 2017) | 26 | 26 | 45.58 | 38.46 | 100 | n-back task |  |  | X |  |
| BD | Singh 2010 (M. K. Singh et al., 2010) | 26 | 22 | 13.2 | 46.67 | 0 | Go/no-go task |  |  | X |  |
| BD | Strakowski 2004 (Strakowski, Adler, Holland, Mills, & DelBello, 2004) | 10 | 10 | 25.5 | 40 | 0 | Continuous performance test |  |  | X |  |
| BD | Strakowski 2008 (Strakowski et al., 2008) | 16 | 16 | 19 | 75 | 50 | Stop-signal task |  |  | X |  |
| BD | Young 2016 (Kymberly D Young et al., 2016) | 30 | 30 | 37.6 | 12.5 | 0 | Autobiographical memory task | X |  | X |  |
| BD | Adler 2004 (Adler, Holland, Schmithorst, Tuchfarber, & M Strakowski, 2004) | 15 | 15 | 29 | NR | 66.67 | n-back task |  |  | X |  |
| BD | Curtis 2001 (Curtis et al., 2001) | 5 | 5 | 33.2 | 100 | 100 | Verbal fluency task + semantic decision task |  |  | X |  |
| BD | Deckerbasch 2008 (Deckersbach et al., 2008) | 9 | 17 | 27.6 | 0 | 100 | Emotional n-back task |  |  | X |  |
| BD | Dickstein 2007 (Dickstein et al., 2007) | 16 | 16 | 14.1 | 56.25 | 81.25 | Reversal learning task |  | X | X |  |
| BD | Drapier 2008 (Drapier et al., 2008) | 20 | 20 | 42.7 | 45 | 80 | n-back task | X |  |  |  |
| BD | Rey 2012 hypomanic (Rey et al., 2014) | 9 | 12 | 42.6 | 66.67 | 100 | Emotional Stroop task |  |  | X |  |
| BD | Rey 2012 depressive (Rey et al., 2014) | 9 | 12 | 42.6 | 66.67 | 100 | Emotional Stroop task |  |  | X | X |
| BD | Rey 2012 euthymic (Rey et al., 2014) | 11 | 12 | 42.6 | 66.67 | 100 | Emotional Stroop task |  |  | X | X |
| BD | Weathers 2013 child (Weathers et al., 2013) | 15 | 20 | 14.74 | 53.3 | 60 | Stop-signal task |  |  | X | X |
| BD | Weathers 2013 adult (Weathers et al., 2013) | 23 | 27 | 40.85 | 55 | 95.7 | Stop-signal task |  |  | X |  |
| *Note.* Dx = diagnosis; SCZ = schizophrenia; MDD = major depressive disorder; BD = bipolar disorder; FTD = forma thought disorder; NR = not reported; Hx = history; AVH = auditory-verbal hallucination; Pos = positive valence system; Neg = negative valence system; Cogn = cognitive system; Social = social processes. Studies published in 2018 or later are from the literature search. | | | | | | | | | | | |

# **Supplementary Figure 3**. Results of the Clustering Analyses showing that the 7-cluster & 11-cluster solutions were the most optimal number of clusters.


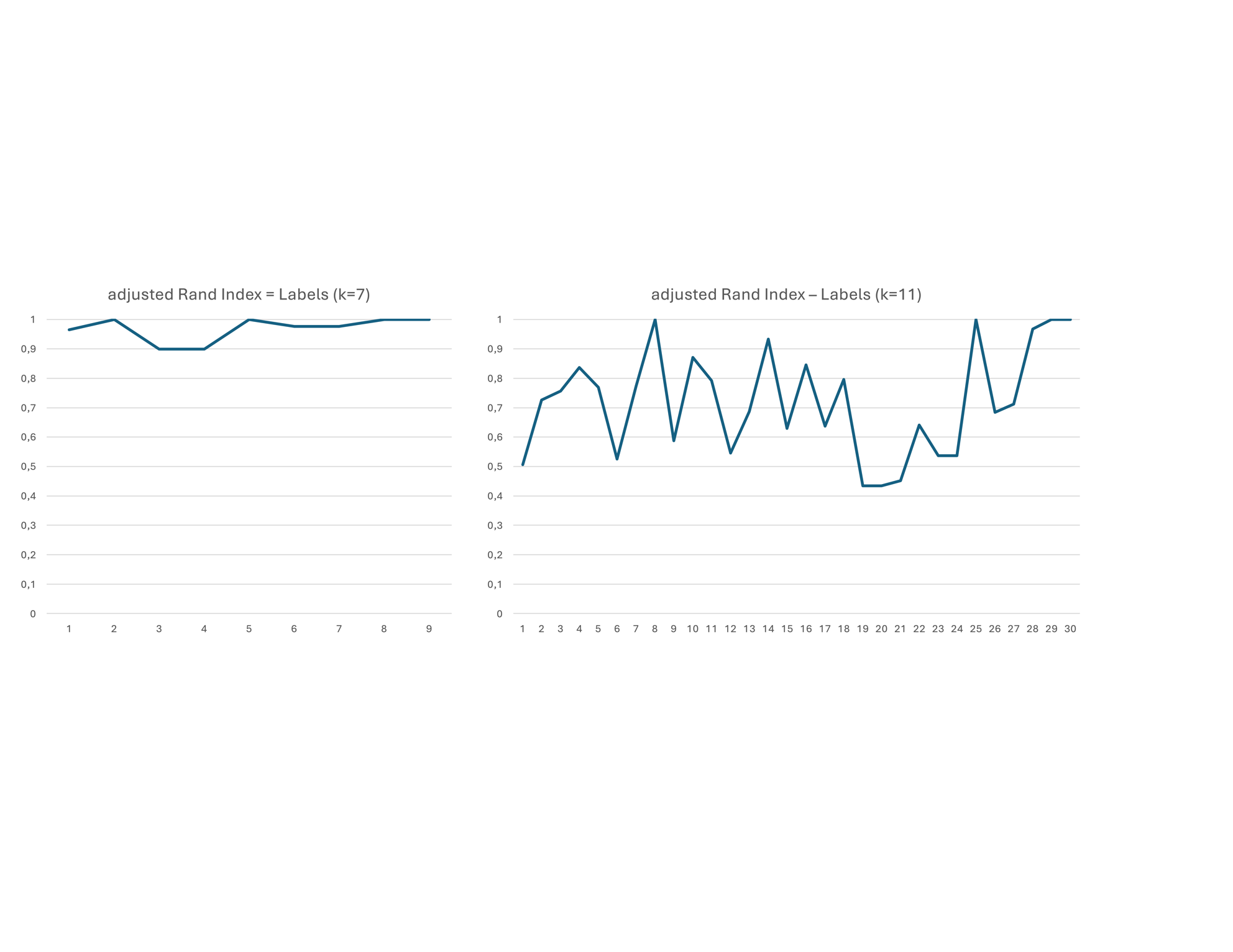


We identified the most stable labeling solutions by comparing labels between iterations using the adjusted Rand index, repeating the process until we got consistent results for at

| **Supplementary Table 2.** Metrics for goodness of clustering. | | | | | | | | | | | | | | |
| --- | --- | --- | --- | --- | --- | --- | --- | --- | --- | --- | --- | --- | --- | --- |
| Metrics | Cluster Solution | | | | | | | | | | | | | |
|  | k=2 | k=3 | k=4 | k=5 | k=6 | k=7 | k=8 | k=9 | k=10 | k=11 | k=12 | k=13 | k=14 | k=15 |
| **Silhouette Index** |  |  |  |  |  |  |  |  |  |  |  |  |  |  |
| Spearman_Average | -0,250 | 1,079 | 0,810 | 0,515 | 0,115 | 1,317 | 1,336 | 2,082 | 1,633 | 1,616 | 1,527 | 1,142 | 1,514 | 1,484 |
| Spearman_Complete | 2,729 | 2,187 | 2,416 | 2,478 | 1,988 | 1,580 | 1,771 | 1,945 | 1,794 | 1,952 | 1,924 | 1,801 | 1,858 | 2,185 |
| Pearson_Average | -1,275 | -1,149 | -0,486 | 0,873 | 0,849 | 1,716 | 1,763 | 1,767 | 1,909 | 1,748 | 1,956 | 1,753 | 2,084 | 1,964 |
| Pearson_Complete | 1,922 | 0,739 | 1,371 | 2,501 | 1,980 | 2,356 | 2,749 | 2,401 | 2,187 | 1,190 | 1,730 | 2,112 | 2,367 | 2,804 |
|  |  |  |  |  |  |  |  |  |  |  |  |  |  |  |
| **Calinski-Harabasz Index** | |  |  |  |  |  |  |  |  |  |  |  |  |  |
| Spearman_Average | 1,728 | 1,672 | 1,666 | 1,552 | 1,515 | 1,623 | 1,548 | 1,593 | 1,648 | 1,638 | 1,687 | 1,652 | 1,671 | 1,672 |
| Spearman_Complete | 1,955 | 2,065 | 1,895 | 1,806 | 1,689 | 1,723 | 1,741 | 1,681 | 1,683 | 1,639 | 1,710 | 1,680 | 1,677 | 1,643 |
| Pearson_Average | 0,019 | 0,464 | 0,924 | 1,235 | 1,278 | 1,433 | 1,473 | 1,546 | 1,573 | 1,569 | 1,594 | 1,616 | 1,639 | 1,642 |
| Pearson_Complete | 1,940 | 1,944 | 1,885 | 1,787 | 1,724 | 1,696 | 1,775 | 1,856 | 1,832 | 1,890 | 1,892 | 1,883 | 1,882 | 1,835 |
|  |  |  |  |  |  |  |  |  |  |  |  |  |  |  |
| **adjusted Rand Index** | |  |  |  |  |  |  |  |  |  |  |  |  |  |
| Spearman_Average | -0,109 | 0,425 | 0,247 | 0,296 | 0,339 | -0,467 | -0,344 | 0,281 | 0,260 | 0,244 | -0,174 | -0,168 | -1,061 | -1,058 |
| Spearman_Complete | -0,066 | 0,134 | 0,180 | 0,217 | 0,142 | 0,209 | -0,439 | -0,479 | -0,533 | -0,541 | 0,502 | 0,543 | 0,577 | 0,584 |
| Pearson_Average | 0,084 | 0,090 | -0,002 | 0,002 | 0,010 | 0,161 | 0,199 | 0,192 | 0,227 | 0,215 | 0,226 | 0,240 | 0,288 | 0,280 |
| Pearson_Complete | 0,141 | 0,217 | 0,369 | 0,359 | 0,427 | 0,447 | 0,493 | 0,616 | 0,662 | 0,674 | 0,695 | 0,783 | 0,801 | 0,664 |

# **Supplementary Figure 4**. Results of the Clustering Analyses


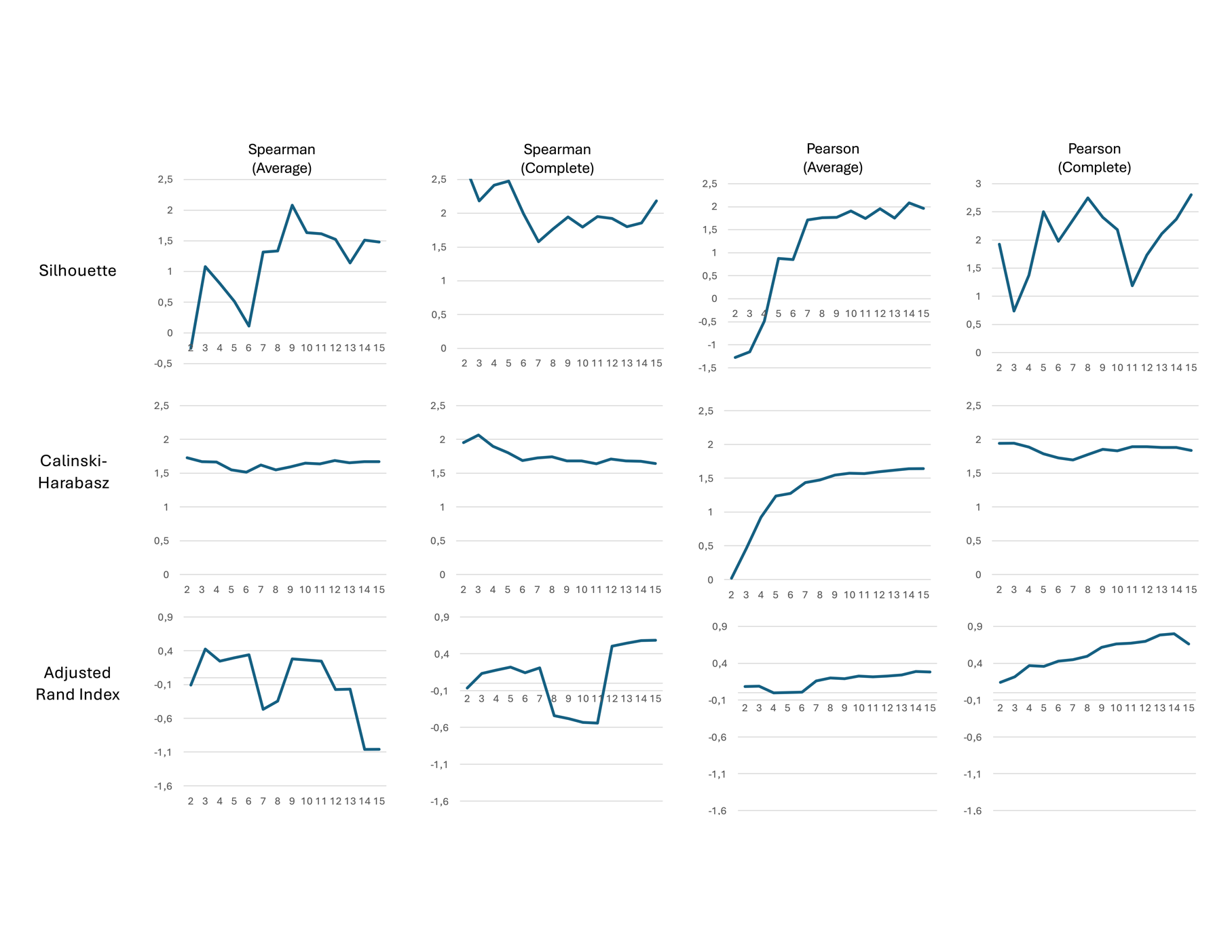


# **
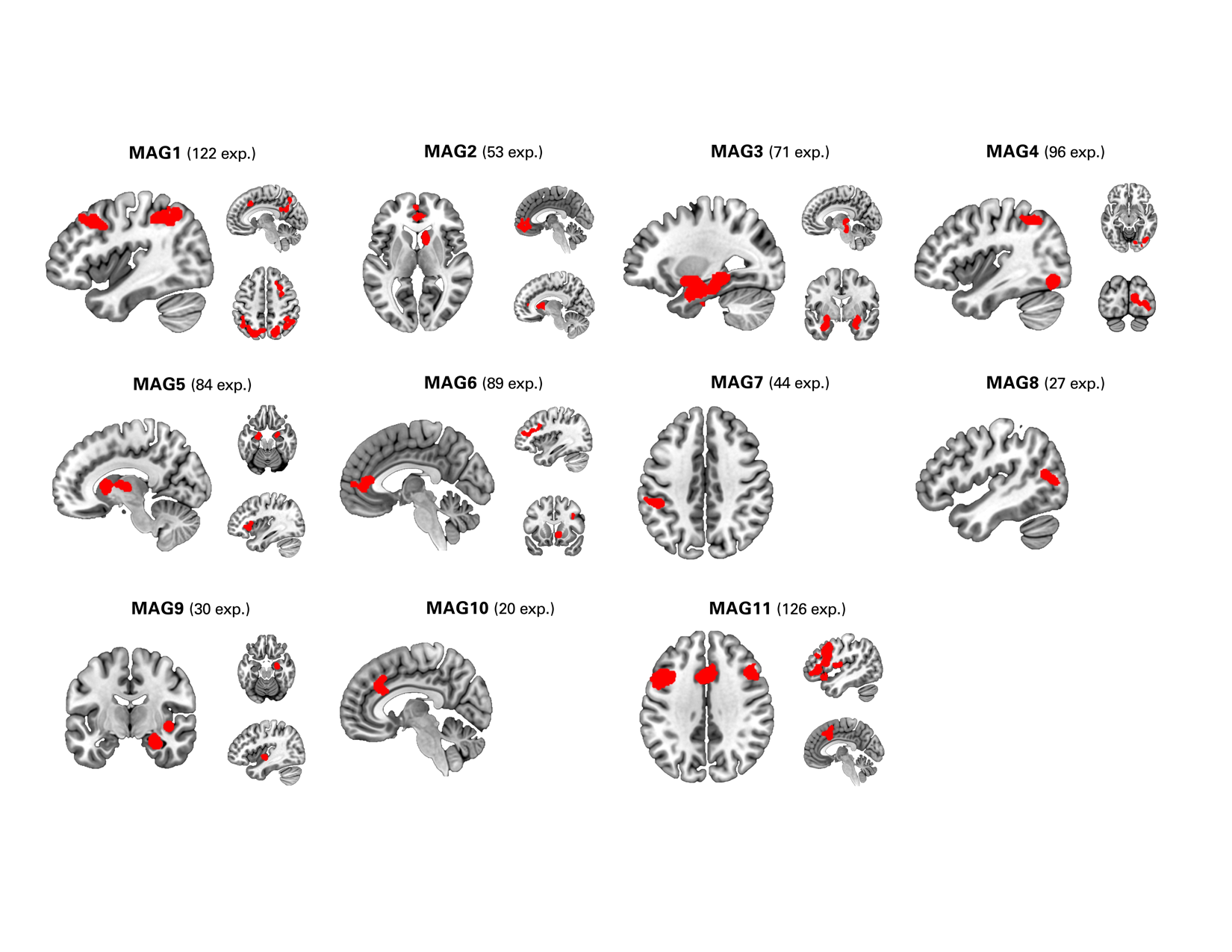
Supplementary Figure 5.** Meta-analytic Results for the 11-MAG solution (pFWE<0.05).

| **Supplementary Table 3**. 11-MAG Solution | | | | |  |
| --- | --- | --- | --- | --- | --- |
| Findings | MNI Coordinates | | | Cluster size (mm3) |  |
|  | x | y | z |  |  |
| MAG1 (k=122) | |  |  |  |  |
| sLOC | -22 | -66 | 46 | 7160 |  |
| aMCC | -2 | 20 | 38 | 1744 |  |
| SPL | 32 | -56 | 46 | 7312 |  |
| dlPFC | 42 | 18 | 40 | 1720 |  |
| FEF | 24 | 10 | 48 | 1272 |  |
| MAG2 (k=53) | |  |  |  |  |
| vmPFC | -4 | 44 | -6 | 6184 |  |
| Caudate | 10 | 12 | 6 | 1216 |  |
| MAG3 (k=71) | |  |  |  |  |
| HIP | -28 | -18 | -16 | 9728 |  |
| BrainStem | -6 | -26 | -8 | 1976 |  |
| HIP | 26 | -34 | -10 | 2360 |  |
| AMY | 26 | 0 | -16 | 2704 |  |
| MAG4 (k=96) | |  |  |  |  |
| Lingual Gyrus | 18 | -88 | 2 | 3152 |  |
| SPL | 36 | -50 | 48 | 1304 |  |
| iLOC | 40 | -72 | -12 | 1656 |  |
| MAG5 (k=84) | |  |  |  |  |
| AMY | -20 | -8 | -16 | 2248 |  |
| Thalamus | 4 | -4 | 6 | 6832 |  |
| AMY | 24 | 0 | -14 | 2304 |  |
| aINS | 36 | 20 | 2 | 2432 |  |
| MAG6 (k=89) | |  |  |  |  |
| pgACC | -2 | 42 | 4 | 4504 |  |
| dlPFC | 36 | 30 | 22 | 2240 |  |
| Caudate | 10 | 16 | -4 | 1112 |  |
| MAG7 (k=44) | |  |  |  |  |
| SMG | -48 | -34 | 40 | 1000 |  |
| MAG8 (k=27) | |  |  |  |  |
| iLOC | -46 | -68 | 12 | 1704 |  |
| MAG9 (k=30) | |  |  |  |  |
| AMY | 26 | -6 | -22 | 3040 |  |
| Insula | 40 | -12 | -4 | 904 |  |
| MAG10 (k=20) | |  |  |  |  |
| aMCC | 4 | 24 | 34 | 2536 |  |
| MAG11 (k=126) | |  |  |  |  |
| IFG | -42 | 16 | 14 | 19432 |  |
| Putamen | -20 | 2 | 8 | 1624 |  |
| aMCC | 0 | 16 | 42 | 6216 |  |
| dlPFC | 48 | 16 | 26 | 2584 |  |
| Lateral PFC | 40 | 40 | 18 | 1336 |  |
| Note. sLOC = superior Lateral Occipital Cortex; aMCC = anterior MidCingulate Cortex; SPL = Superior Parietal Lobule; dlPFC = dorsolateral PFC; FEF = Frontal Eye Fields; vmPFC = ventromedial PFC; HIP = Hippocampus; AMY = Amygdala; SPL = Superior Parietal Lobule; iLOC = Inferior LOC; aINS = anterior Insula; pgACC = perigenual ACC; IFG = Inferior Frontal Gyrus | | | | |  |
|  |  |  |  |  |  |

| **Supplementary Table 4**. ALE meta-analysis results corrected per Meta-analytical Groupings and results of between-group comparisons of probabilities of activation for each region using a threshold of p < 0.05 corrected with false discovery rate (FDR). | | | | | | | | |
| --- | --- | --- | --- | --- | --- | --- | --- | --- |
| Regions | L/R | MNI Coordinates | | | ALE values | Cluster size (mm^3^) | Hyper-Hypoactivation combined | |
|  |  | x | y | z |  |  | Statistic | pFDR corrected |
| *MAG1* | | |  |  |  |  |  |  |
| Network |  |  |  |  |  |  | H(2, 99)=4.20 | 0.122 |
| AMY/aHIP | R | 26 | -4 | -16 |  | 4552 | H(2, 99)=4.55 | 0.2575 |
| AMY/aHIP | L | -26 | -10 | -18 |  | 7600 | H(2, 99)=2.78 | 0.4167 |
| Lobule V | R | 2 | -58 | -16 |  | 1328 | H(2, 99)=1.74 | 0.5225 |
| MTG | R | 56 | -8 | -16 |  | 2112 | H(2, 99)=0.82 | 0.664 |
| IPL | R | 50 | -60 | 20 |  | 5880 | H(2, 99)=12.79 | 0.01* |
| *MAG2* | | |  |  |  |  |  |  |
| Network |  |  |  |  |  |  | H(2, 395)=6.50 | 0.039* |
| dlPFC | L | -46 | 8 | 32 |  | 8264 | H(2, 395)=2.28 | 0.448 |
| aINS | L | -38 | 24 | -2 |  | 5368 | H(2, 395)=7.59 | 0.154 |
| SPL | L | -34 | -56 | 46 |  | 3872 | H(2, 395)=0.14 | 0.932 |
| Thalamus | L | -4 | -8 | 6 |  | 6576 | H(2, 395)=5.80 | 0.193 |
| aMCC | - | 0 | 16 | 42 |  | 9720 | H(2, 395)=3.41 | 0.322 |
| aINS | R | 34 | 22 | -2 |  | 25288 | H(2, 395)=3.39 | 0.322 |
| SPL | R | 34 | -54 | 50 |  | 7288 | H(2, 395)=1.87 | 0.459 |
| *MAG3* | |  |  |  |  |  |  |  |
| Network |  |  |  |  |  |  | H(2, 60)=1.52 | 0.468 |
| AG | L | -44 | -60 | 28 |  | 2720 | H(2, 395)=0.47 | 0.969 |
| Frontal Eye Field | L | -30 | 10 | 52 |  | 6312 | H(2, 395)=0.06 | 0.969 |
| Intracalcarine Cortex | L | -10 | -66 | 8 |  | 2336 | H(2, 60)=2.87 | 0.558 |
| Precentral Gyrus | R | 40 | -16 | 46 |  | 1776 | H(2, 60)=2.55 | 0.558 |
| *MAG4* | | |  |  |  |  |  |  |
| Network |  |  |  |  |  |  | H(2, 183)=11.45 | 0.003* |
| Striatum | L | -16 | 12 | 10 |  | 4592 | H(2, 183)= 12.96 | 0.008* |
| pgACC/vmPFC | L | -2 | 42 | 4 |  | 19184 | H(2, 183)=12.96 | 0.008* |
| Striatum | R | 18 | 2 | 10 |  | 3000 | H(2, 183)=5.91 | 0.064 |
| PCC | L | -4 | -40 | 30 |  | 3192 | H(2, 183)=9.32 | 0.015* |
| Central opercular | R | 42 | 4 | 10 |  | 2960 | H(2, 183)=2.01 | 0.366 |
| *MAG5* | | |  |  |  |  |  |  |
| Network |  |  |  |  |  |  | H(2, 27)=2.93 | 0.231 |
| Lingual Gyrus | L | -28 | -54 | 2 |  | 4616 | H(2, 27)=3.02 | 0.546 |
| pHIP | R | 28 | -34 | 0 |  | 4552 | H(2, 27)=1.56 | 0.546 |
| Lateral PFC | L | -34 | 50 | -12 |  | 1392 | H(2, 27)=1.21 | 0.546 |
| *Note.* MNI = Montreal Neurological Institute; MAG = Meta-analytical Grouping; L = Left; R = Right; FDR = False-Discovery Rate; AMY = Amygdala; aHIP = Anterior Hippocampus; pHIP = Posterior Hippocampus; MTG = Middle Temporal Gyrus; pgACC/vmPFC = Perigenual Anterior Cingulate Cortex and Ventromedial Prefrontal Cortex; dlPFC = Dorsolateral Prefrontal Cortex; aMCC = Anterior Midcingulate Cortex; SPL = Superior Parietal Lobule; AG = Angular Gyrus; IPL = Inferior Parietal Lobule; PCC = Posterior Cingulate Cortex; aINS = Anterior Insula. * = Significant with a threshold of pFDR<0.05 corrected. | | | | | | | | |

| **Supplementary Table 5**. Results from the Spearman rank correlations for confondant variables. | | | | | | | | | |
| --- | --- | --- | --- | --- | --- | --- | --- | --- | --- |
| Region | Medication rate | | | Age | | | Sex ratio (% females) | | |
|  | Correlation coefficient | p-value uncorrected | pFDR corrected | Correlation coefficient | p-value uncorrected | pFDR corrected | Correlation coefficient | p-value uncorrected | pFDR corrected |
| MAG1 – R AMY/aHIP | 0.102 | 0.332 | 0.488 | 0.006 | 0.956 | 0.978 | -0.105 | 0.307 | 0.581 |
| MAG1 – L AMY/aHIP | -0.106 | 0.312 | 0.488 | 0.003 | 0.978 | 0.978 | 0.043 | 0.676 | 0.676 |
| MAG1 – Lobule V | -0.038 | 0.716 | 0.716 | 0.065 | 0.523 | 0.978 | -0.072 | 0.484 | 0.581 |
| MAG1 – MTG | -0.087 | 0.407 | 0.488 | -0.105 | 0.302 | 0.978 | 0.103 | 0.318 | 0.581 |
| MAG1 – IPL | -0.206 | 0.047 | 0.141 | -0.038 | 0.710 | 0.978 | 0.100 | 0.335 | 0.581 |
| MAG1 - Network | -0.241 | 0.020 | 0.120 | -0.064 | 0.530 | 0.978 | -0.075 | 0.467 | 0.581 |
| MAG2 – dlPFC | 0.017 | 0.755 | 0.961 | -0.079 | 0.127 | 0.339 | 0.120 | 0.020 | 0.080 |
| MAG2 – aINS | -0.003 | 0.959 | 0.961 | -0.087 | 0.091 | 0.339 | 0.140 | 0.007 | 0.056 |
| MAG2 – SPL | 0.057 | 0.299 | 0.961 | -0.051 | 0.326 | 0.6368 | 0.001 | 0.989 | 0.989 |
| MAG2 – Thalamus | -0.022 | 0.691 | 0.961 | -0.014 | 0.792 | 0.792 | 0.004 | 0.933 | 0.989 |
| MAG2 – aMCC | -0.061 | 0.265 | 0.961 | -0.034 | 0.506 | 0.661 | 0.046 | 0.379 | 0.606 |
| MAG2 – aINS | -0.003 | 0.961 | 0.961 | -0.044 | 0.398 | 0.637 | 0.038 | 0.460 | 0.613 |
| MAG2 – SPL | 0.033 | 0.544 | 0.961 | -0.029 | 0.578 | 0.661 | -0.061 | 0.239 | 0.478 |
| MAG2 – Network | -0.034 | 0.534 | 0.961 | -0.084 | 0.106 | 0.339 | 0.093 | 0.072 | 0.192 |
| MAG3 – AG | 0.114 | 0.407 | 0.671 | 0.076 | 0.566 | 0.708 | 0.146 | 0.270 | 0.628 |
| MAG3 – Frontal eye field | 0.082 | 0.551 | 0.671 | 0.104 | 0.430 | 0.708 | 0.112 | 0.399 | 0.628 |
| MAG3 – Intracalcarine cortex | 0.128 | 0.352 | 0.671 | -0.273 | 0.035 | 0.175 | 0.139 | 0.295 | 0.628 |
| MAG3 – Precentral gyrus | -0.249 | 0.067 | 0.335 | -0.042 | 0.750 | 0.750 | -0.089 | 0.502 | 0.628 |
| MAG3 – Network | 0.058 | 0.671 | 0.671 | -0.116 | 0.378 | 0.708 | 0.026 | 0.846 | 0.846 |
| MAG4 – L striatum | -0.113 | 0.147 | 0.364 | -0.137 | 0.070 | 0.150 | 0.063 | 0.408 | 0.490 |
| MAG4 – pgACC/vmPFC | -0.008 | 0.924 | 0.983 | 0.081 | 0.285 | 0.342 | -0.105 | 0.053 | 0.159 |
| MAG4 – R striatum | -0.027 | 0.727 | 0.983 | 0.009 | 0.910 | 0.910 | 0.173 | 0.022 | 0.132 |
| MAG4 – PCC | 0.002 | 0.983 | 0.983 | -0.124 | 0.100 | 0.150 | 0.095 | 0.210 | 0.315 |
| MAG4 – Central opercular | -0.104 | 0.182 | 0.364 | -0.126 | 0.095 | 0.150 | 0.042 | 0.581 | 0.581 |
| MAG4 – Network | -0.135 | 0.083 | 0.364 | -0.205 | 0.006 | 0.036* | 0.100 | 0.185 | 0.315 |
| MAG5 – Lingual gyrus | 0.039 | 0.857 | 0.857 | -0.204 | 0.316 | 0.557 | -0.070 | 0.735 | 0.926 |
| MAG5 – pHIP | -0.042 | 0.846 | 0.857 | 0.857 | 0.418 | 0.557 | 0.024 | 0.906 | 0.926 |
| MAG5 – Lateral PFC | 0.174 | 0.417 | 0.834 | -0.058 | 0.779 | 0.779 | -0.130 | 0.528 | 0.926 |
| MAG5 - Network | 0.381 | 0.066 | 0.264 | -0.238 | 0.242 | 0.557 | -0.019 | 0.926 | 0.926 |
| *Note.* MNI = Montreal Neurological Institute; MAG = Meta-analytical Grouping; L = Left; R = Right; FDR = False-Discovery Rate; AMY = Amygdala; aHIP = Anterior Hippocampus; pHIP = Posterior Hippocampus; MTG = Middle Temporal Gyrus; pgACC/vmPFC = Perigenual Anterior Cingulate Cortex and Ventromedial Prefrontal Cortex; dlPFC = Dorsolateral Prefrontal Cortex; aMCC = Anterior Midcingulate Cortex; SPL = Superior Parietal Lobule; AG = Angular Gyrus; IPL = Inferior Parietal Lobule; PCC = Posterior Cingulate Cortex; aINS = Anterior Insula. * = Significant with a threshold of pFDR<0.05 corrected. | | | | | | | | | |

# **Methods: ALE classical approach**

A disorder-specific meta-analysis was performed for each diagnostic group using ALE approach (GingerALE version 3.0.2, http://www.brainmap.org/ale/). Study results were extracted, irrespectively of the direction (decreased/increased) or task-contrast effect, to create an aberrant activation map. Coordinates of experiments that were reported originally in Talairach stereotaxic space were converted into MNI (Montreal Neurologic Institute) space before using them in the analyses. First, a modeled activation map (MA) was created by modeling coordinate foci (x,y,z) with a spherical Gaussian probability distribution, weighted by the number of subjects in each experiment. This is performed to account for spatial uncertainty due to template and between-subject variance (504) and ensure that multiple coordinates from a single experiment does not jointly influence the modeled activation value of a single voxel. Voxel-wise ALE scores were then computed as the union of MA maps, which provide a quantitative assessment of convergence between brain activation across experiments. Then, these maps were cut off by a cluster-forming threshold. In fact, the size of the supra-threshold clusters was compared against a null distribution of cluster sizes derived from artificially created datasets in which foci were shuffled across experiments, but the other properties of original experiments (e.g., number of foci, uncertainty) were kept. Finally, this resulted in calculating the above chance of observing a cluster of the given size (505). Consistent with previous meta-analyses, we use the following statistical threshold: a voxel-level cluster forming threshold of p < 0.001 and a cluster-level family- wise correction (pFWE < 0.05), with 5,000 permutations (506).

# **Results: Disorder-Specific ALE Meta-analyses**

Briefly, regardless of directionality, the meta-analysis focusing on schizophrenia (SCZ) depicted alterations of the bilateral (dorso)lateral prefrontal cortex (dlPFC) and the midcingulate cortex, with a large cluster in the right anterior insula (see Supplementary Tables 5, 6 and 7 as well as Supplementary Figures 4, 5 and 6 for complete results of each meta-analysis). The meta-analysis grouping major depressive disorder (MDD) studies showed alterations of (para)limbic (perigenual anterior cingulate cortex and amygdala) and motor regions (e.g. precentral gyrus and supplementary motor area). The meta-analysis on bipolar disorder (BD) showed alterations of the lateral prefrontal cortex, putamen and limbic regions (e.g., amygdala), with a large cluster in the ventromedial prefrontal cortex (vmPFC). Subanalyses revealed large clusters of hyperactivation in the left dlPFC in SCZ and the vmPFC/OFC in BD, and a large cluster of hypoactivation in the right anterior insular in SCZ.

| **Supplementary Table 6.** Meta-analysis of fMRI studies on Schizophrenia | | | | | |  |
| --- | --- | --- | --- | --- | --- | --- |
| Analyses | MNI Coordinates | | | Cluster Mass (Z) | Cluster Size (mm3) |  |
|  | X | Y | Z |  |  |  |
| Across Direction |  |  |  |  |  |  |
| dlPFC | -46 | 10 | 32 | 3,353 | 3760 |  |
| Thalamus/Caudate | 2 | -8 | 6 | 3,353 | 4504 |  |
| dACC | 2 | 18 | 38 | 3,353 | 5568 |  |
| vSTR/AMY/aINS | 20 | 12 | -4 | 3,353 | 7984 |  |
| vlPFC/aINS | -34 | 20 | -6 | 3,156 | 2072 |  |
| dlPFC | 50 | 18 | 22 | 3,036 | 3000 |  |
| dStr | -18 | 8 | 10 | 2,878 | 1440 |  |
| Lateral PFC | 40 | 40 | 20 | 2,770 | 2280 |  |
| SPL | 40 | -50 | 48 | 2,562 | 2008 |  |
| SPL | -40 | -46 | 48 | 0,283 | 720 |  |
| pITG | -46 | -54 | -10 | 0,217 | 528 |  |
| SPL | -32 | -58 | 50 | 0,141 | 472 |  |
| Increased Activation |  |  |  |  |  |  |
| dlPFC | -50 | 12 | 30 | 3,353 | 2984 |  |
| Auditory Cortex | -48 | -36 | 16 | 2,489 | 936 |  |
| SFG | 20 | 22 | 46 | 0,241 | 432 |  |
| Decreased Activation |  |  |  |  |  |  |
| vlPFC/aINS | -36 | 18 | -6 | 3,353 | 2528 |  |
| Thalamus/AMY | -4 | -10 | 4 | 3,353 | 6528 |  |
| dACC | 2 | 20 | 40 | 3,353 | 3216 |  |
| vSTR/AMY/aINS | 22 | 16 | -2 | 3,353 | 8352 |  |
| dStr | -18 | 8 | 10 | 3,036 | 1432 |  |
| SPL | 40 | -52 | 48 | 2,770 | 1872 |  |
| IFG/dlPFC | 52 | 22 | 14 | 2,409 | 1656 |  |
| Lateral PFC | 42 | 38 | 20 | 2,155 | 1616 |  |
| dlPFC | 44 | 16 | 36 | 0,908 | 840 |  |
| AG | 50 | -56 | 16 | 0,767 | 784 |  |
| IFG | -48 | 24 | 8 | 0,097 | 840 |  |
| Note. Cluster-level FWE-corrected results. dlPFC = dorsolateral Prefrontal Cortex; dACC = dorsal Anterior Cingulate Cortex; vSTR = ventral Striatum; AMY = Amygdala; aINS = anterior insula; vlPFC = ventrolateral PFC; dSTR = dorsal Striatum; SPL = Superior Parietal Lobule; pITG = posterior Inferior Temporal Gyrus; SFG = Superior Frontal Gyrus; IFG = Inferior Frontal Gyrus; AG = Angular Gyrus | | | | | |  |
|  |  |  |  |  |  |  |

| **Supplementary Table 7.** Meta-analysis of fMRI studies on Major Depressive Disorder | | | | | |  |
| --- | --- | --- | --- | --- | --- | --- |
| Analyses | MNI Coordinates | | | Cluster Mass (Z) | Cluster Size (mm3) |  |
|  | X | Y | Z |  |  |  |
| Across Direction | |  |  |  |  |  |
| pgACC/mPFC | -4 | 44 | 6 | 2,468 | 1872 |  |
| dSTR | -14 | 4 | 16 | 2,428 | 1024 |  |
| pre-SMA | 0 | 10 | 54 | 1,600 | 824 |  |
| AMY | 22 | -4 | -14 | 1,419 | 752 |  |
| PreCG | 40 | -16 | 46 | 0,686 | 616 |  |
| Thalamus | -12 | -8 | 10 | 0,363 | 680 |  |
| Increased Activation | |  |  |  |  |  |
| PreCG | 40 | -16 | 46 | 2,175 | 856 |  |
| pHIP | -30 | -32 | -10 | 1,080 | 640 |  |
| AMY | 22 | -2 | -14 | 1,077 | 656 |  |
| PoCG | -46 | -32 | 60 | 0,590 | 424 |  |
| Lingual Gyrus | 0 | -90 | -6 | 0,514 | 400 |  |
| Cuneus | -6 | -88 | 28 | 0,392 | 448 |  |
| dSTR | -14 | 2 | 16 | 0,243 | 408 |  |
| SMA | 0 | 8 | 54 | 0,144 | 528 |  |
| Decreased Activation | |  |  |  |  |  |
| mPFC/pgACC | -2 | 54 | 0 | 1,016 | 760 |  |
| Parietal Operculum | 46 | -36 | 20 | 0,443 | 640 |  |
| MFG | 34 | 18 | 42 | 0,137 | 672 |  |
| Note. Cluster-level FWE-corrected results. pgACC = perigenual Anterior Cingulate Cortex; mPFC = medial Prefrontal Cortex; dSTR = dorsal Striatum; pre-SMA = pre-supplementary motor area; AMY = Amygdala; PreCG = Precentral Gyrus; pHIP = posterior Hippocampus; PoCG = Postcentral Gyrus; SMA = Supplementary Motor Area; MFG = Middle Frontal Gyrus | | | | | |  |
|  |  |  |  |  |  |  |

| **Supplementary Table 8.** Meta-analysis of fMRI studies on Bipolar Disorder | | | | | |  |
| --- | --- | --- | --- | --- | --- | --- |
| Analyses | MNI Coordinates | | | Cluster Mass (Z) | Cluster Size (mm3) |  |
|  | X | Y | Z |  |  |  |
| Across Direction | |  |  |  |  |  |
| pgACC/vmPFC | -4 | 40 | -8 | 3,353 | 5304 |  |
| vSTR/AMY | 26 | 2 | -10 | 2,652 | 1936 |  |
| MFG | -34 | -2 | 54 | 1,279 | 776 |  |
| IFG | 48 | 34 | 6 | 1,231 | 976 |  |
| dSTR | -20 | 4 | 6 | 1,128 | 856 |  |
| Precuneus | -10 | -70 | 38 | 0,695 | 528 |  |
| PreCG | -44 | 0 | 32 | 0,281 | 672 |  |
| Increased Activation | |  |  |  |  |  |
| pgACC/vmPFC | -4 | 42 | -8 | 3,353 | 5792 |  |
| Frontal Pole | 32 | 58 | 2 | 0,273 | 576 |  |
| Decreased Activation | |  |  |  |  |  |
| IFG | 48 | 34 | 6 | 3,353 | 1784 |  |
| Putamen | 24 | 6 | -4 | 2,357 | 1184 |  |
| AMY | 26 | -2 | -16 | 1,282 | 624 |  |
| vlPFC | -32 | 42 | -8 | 1,257 | 728 |  |
| MFG | -32 | -2 | 54 | 1,175 | 560 |  |
| aINS | -32 | 24 | -2 | 0,663 | 680 |  |
| SPL | 10 | -64 | 64 | 0,265 | 336 |  |
| Putamen | -20 | 4 | 0 | 0,107 | 768 |  |
| aHIP | -32 | -12 | -16 | 0,080 | 536 |  |
| SMA | -2 | 12 | 54 | 0,080 | 424 |  |
| Note. Cluster-level FWE-corrected results. pgACC = perigenual Anterior Cingulate Cortex; vmPFC = ventromedial Prefrontal Cortex; MFG = Middle Frontal Gyrus; IFG = Inferior Frontal Gyrus; dSTR = dorsal Striatum; PreCG = Precentral Gyrus; AMY = Amygdala; vlPFC = ventrolateral PFC; aINS = anterior Insula; SPL = Superior Parietal Lobule; aHIP = anterior Hippocampus; SMA = Supplementary Motor Area | | | | | |  |
|  |  |  |  |  |  |  |

# **Supplementary Figure 6.** ALE meta-analysis on Severe Mental Disorders (Across Tasks, Irrespective of the Directionality)


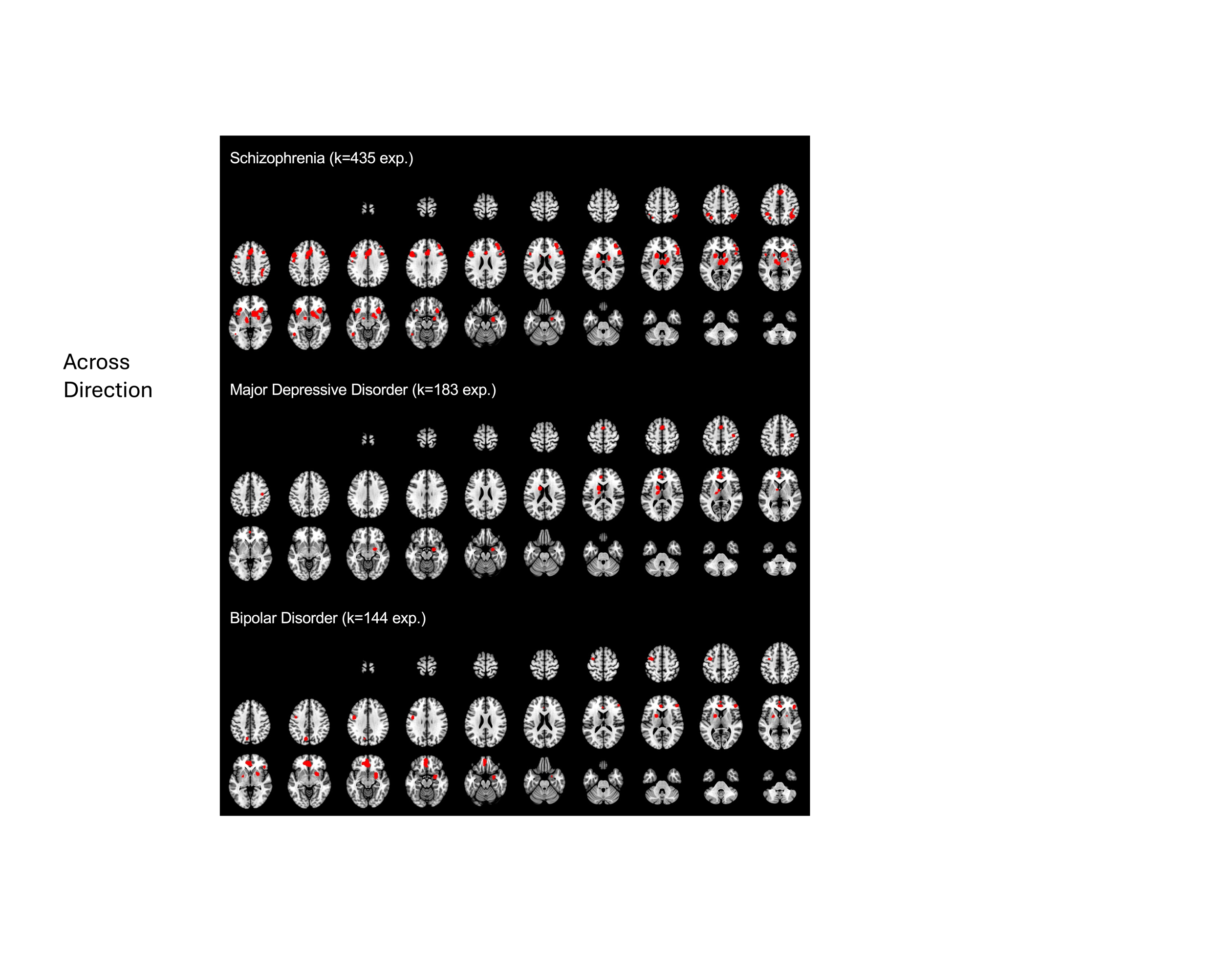


# **Supplementary Figure 7.** ALE meta-analysis on Severe Mental Disorders (Across Tasks, Cases > Controls)


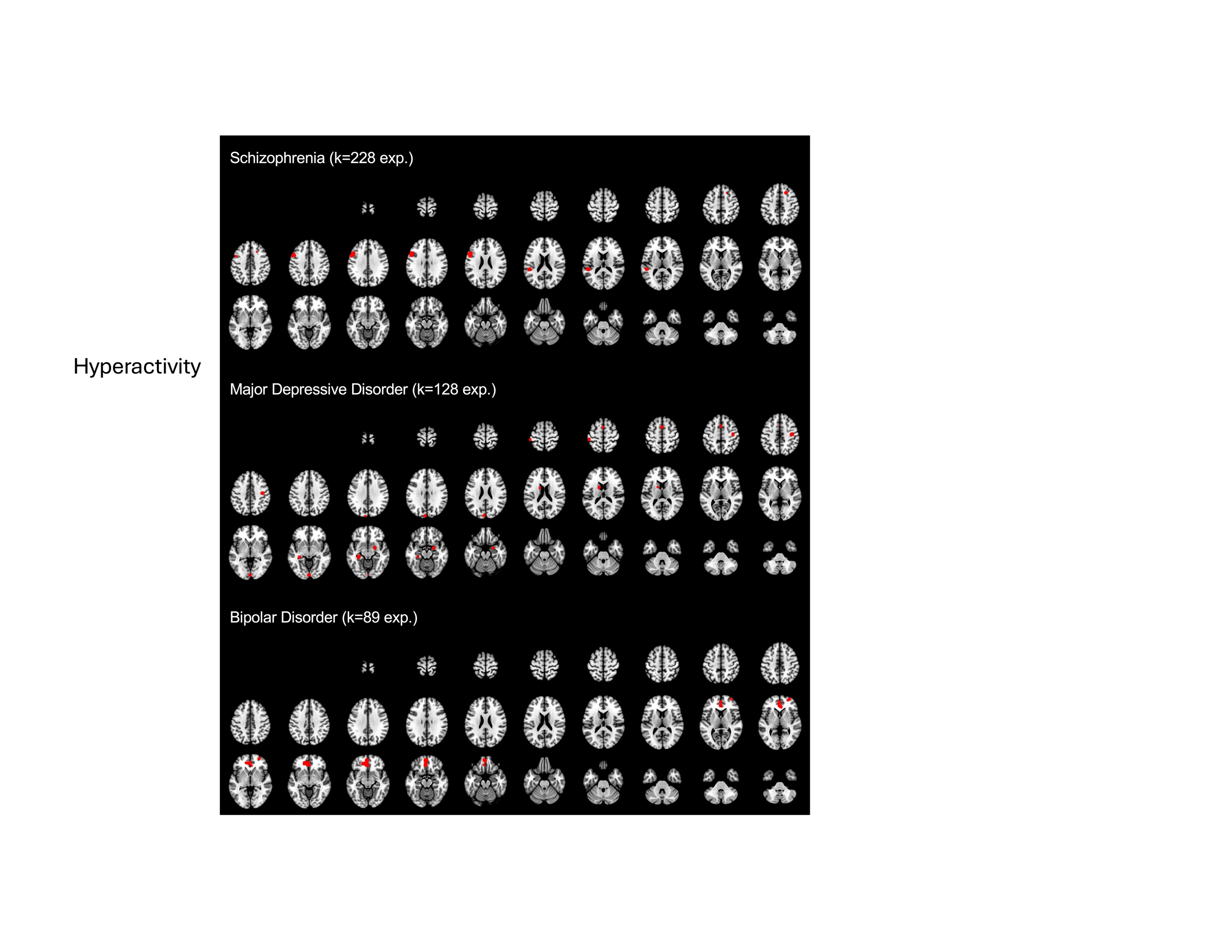


# **Supplementary Figure 8.** ALE meta-analysis on Severe Mental Disorders (Across Tasks, Controls > Cases)


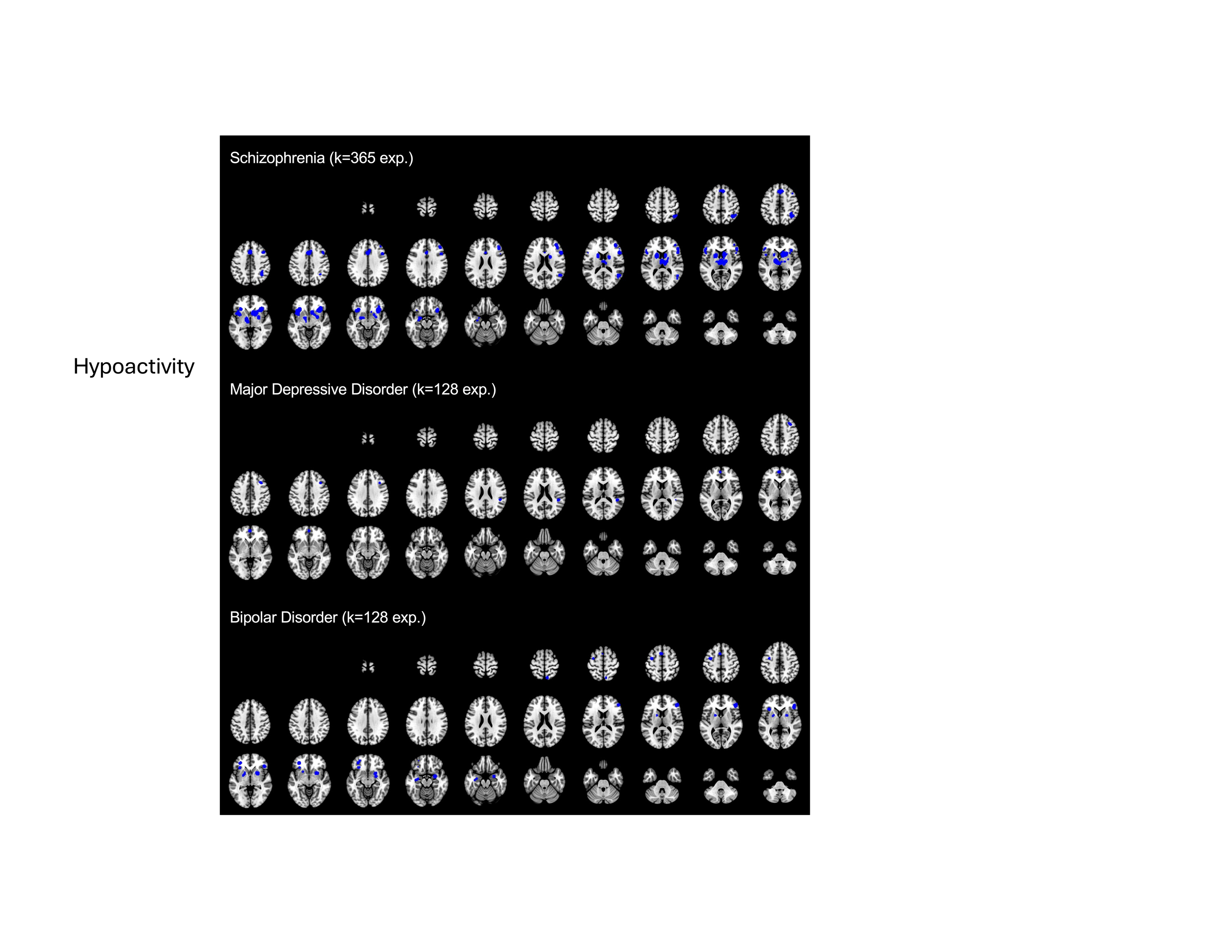


**References**

Achim, A. M., Bertrand, M.-C., Sutton, H., Montoya, A., Czechowska, Y., Malla, A. K., … Lepage, M. (2007). Selective Abnormal Modulation of Hippocampal Activity During Memory Formation in First-Episode Psychosis. *Archives of General Psychiatry*, *64*(9), 999. doi: 10.1001/archpsyc.64.9.999

Adamczyk, P., Jáni, M., Ligeza, T. S., Płonka, O., Błądziński, P., & Wyczesany, M. (2021). On the Role of Bilateral Brain Hypofunction and Abnormal Lateralization of Cortical Information Flow as Neural Underpinnings of Conventional Metaphor Processing Impairment in Schizophrenia: An fMRI and EEG Study. *Brain Topography*, *34*(4), 537–554. doi: 10.1007/s10548-021-00849-x

Adamczyk, P., Wyczesany, M., Domagalik, A., Daren, A., Cepuch, K., Błądziński, P., … Marek, T. (2017). Neural circuit of verbal humor comprehension in schizophrenia—An fMRI study. *NeuroImage: Clinical*, *15*, 525–540. doi: 10.1016/j.nicl.2017.06.005

Adler, C. M., Holland, S. K., Schmithorst, V., Tuchfarber, M. J., & M Strakowski, S. (2004). Changes in neuronal activation in patients with bipolar disorder during performance of a working memory task. *Bipolar Disorders*, *6*(6), 540–549. doi: 10.1111/j.1399-5618.2004.00117.x

Admon, R., Holsen, L. M., Aizley, H., Remington, A., Whitfield-Gabrieli, S., Goldstein, J. M., & Pizzagalli, D. A. (2015). Striatal Hypersensitivity During Stress in Remitted Individuals with Recurrent Depression. *Biological Psychiatry*, *78*(1), 67–76. doi: 10.1016/j.biopsych.2014.09.019

Ai, H., Van Tol, M.-J., Marsman, J.-B. C., Veltman, D. J., Ruhé, H. G., Van Der Wee, N. J. A., … Aleman, A. (2018). Differential relations of suicidality in depression to brain activation during emotional and executive processing. *Journal of Psychiatric Research*, *105*, 78–85. doi: 10.1016/j.jpsychires.2018.08.018

Alders, G. L., Davis, A. D., MacQueen, G., Strother, S. C., Hassel, S., Zamyadi, M., … Hall, G. B. (2019). Reduced accuracy accompanied by reduced neural activity during the performance of an emotional conflict task by unmedicated patients with major depression: A CAN-BIND fMRI study. *Journal of Affective Disorders*, *257*, 765–773. doi: 10.1016/j.jad.2019.07.037

Allin, M. P. G., Marshall, N., Schulze, K., Walshe, M., Hall, M.-H., Picchioni, M., … McDonald, C. (2010). A functional MRI study of verbal fluency in adults with bipolar disorder and their unaffected relatives. *Psychological Medicine*, *40*(12), 2025–2035. doi: 10.1017/S0033291710000127

Alonso‐Lana, S., Moro, N., McKenna, P. J., Sarró, S., Romaguera, A., Monté, G. C., … Pomarol‐Clotet, E. (2019). Longitudinal brain functional changes between mania and euthymia in bipolar disorder. *Bipolar Disorders*, *21*(5), 449–457. doi: 10.1111/bdi.12767

Alonso-Lana, S., Valentí, M., Romaguera, A., Sarri, C., Sarró, S., Rodríguez-Martínez, A., … Pomarol-Clotet, E. (2016). Brain functional changes in first-degree relatives of patients with bipolar disorder: Evidence for default mode network dysfunction. *Psychological Medicine*, *46*(12), 2513–2521. doi: 10.1017/S0033291716001148

Alonso-Lana, Silvia, Goikolea, J. M., Bonnin, C. M., Sarró, S., Segura, B., Amann, B. L., … McKenna, P. J. (2016). Structural and Functional Brain Correlates of Cognitive Impairment in Euthymic Patients with Bipolar Disorder. *PLOS ONE*, *11*(7), e0158867. doi: 10.1371/journal.pone.0158867

Altshuler, L., Bookheimer, S., Townsend, J., Proenza, M. A., Sabb, F., Mintz, J., & Cohen, M. S. (2008). Regional brain changes in bipolar I depression: A functional magnetic resonance imaging study. *Bipolar Disorders*, *10*(6), 708–717. doi: 10.1111/j.1399-5618.2008.00617.x

Altshuler, L. L., Bookheimer, S. Y., Townsend, J., Proenza, M. A., Eisenberger, N., Sabb, F., … Cohen, M. S. (2005). Blunted Activation in Orbitofrontal Cortex During Mania: A Functional Magnetic Resonance Imaging Study. *Biological Psychiatry*, *58*(10), 763–769. doi: 10.1016/j.biopsych.2005.09.012

Anticevic, A., Repovs, G., & Barch, D. M. (2013). Working Memory Encoding and Maintenance Deficits in Schizophrenia: Neural Evidence for Activation and Deactivation Abnormalities. *Schizophrenia Bulletin*, *39*(1), 168–178. doi: 10.1093/schbul/sbr107

Anticevic, A., Repovs, G., Corlett, P. R., & Barch, D. M. (2011). Negative and Nonemotional Interference with Visual Working Memory in Schizophrenia. *Biological Psychiatry*, *70*(12), 1159–1168. doi: 10.1016/j.biopsych.2011.07.010

Apazoglou, K., Küng, A.-L., Cordera, P., Aubry, J.-M., Dayer, A., Vuilleumier, P., & Piguet, C. (2019). Rumination related activity in brain networks mediating attentional switching in euthymic bipolar patients. *International Journal of Bipolar Disorders*, *7*(1), 3. doi: 10.1186/s40345-018-0137-5

Arce, E., Leland, D. S., Miller, D. A., Simmons, A. N., Winternheimer, K. C., & Paulus, M. P. (2006). Individuals with schizophrenia present hypo- and hyperactivation during implicit cueing in an inhibitory task. *NeuroImage*, *32*(2), 704–713. doi: 10.1016/j.neuroimage.2006.04.189

Arcuri, S. M., Broome, M. R., Giampietro, V., Amaro, E., Kircher, T. T. J., Williams, S. C. R., … McGuire, P. K. (2012). Faulty Suppression of Irrelevant Material in Patients with Thought Disorder Linked to Attenuated Frontotemporal Activation. *Schizophrenia Research and Treatment*, *2012*, 1–12. doi: 10.1155/2012/176290

Arnold, A. E. G. F., Iaria, G., & Goghari, V. M. (2016). Efficacy of identifying neural components in the face and emotion processing system in schizophrenia using a dynamic functional localizer. *Psychiatry Research: Neuroimaging*, *248*, 55–63. doi: 10.1016/j.pscychresns.2016.01.007

Arrondo, G., Segarra, N., Metastasio, A., Ziauddeen, H., Spencer, J., Reinders, N. R., … Murray, G. K. (2015). Reduction in ventral striatal activity when anticipating a reward in depression and schizophrenia: A replicated cross-diagnostic finding. *Frontiers in Psychology*, *6*. doi: 10.3389/fpsyg.2015.01280

Assaf, M., Rivkin, P. R., Kuzu, C. H., Calhoun, V. D., Kraut, M. A., Groth, K. M., … Pearlson, G. D. (2006). Abnormal Object Recall and Anterior Cingulate Overactivation Correlate with Formal Thought Disorder in Schizophrenia. *Biological Psychiatry*, *59*(5), 452–459. doi: 10.1016/j.biopsych.2005.07.039

Aust, S. (2013). Music in depression: Neural correlates of emotional experience in remitted depression. *World Journal of Psychiatry*, *3*(2), 8. doi: 10.5498/wjp.v3.i2.8

Avsar, K. B., Stoeckel, L. E., Bolding, M. S., White, D. M., Tagamets, M. A., Holcomb, H. H., & Lahti, A. C. (2011). Aberrant visual circuitry associated with normal spatial match-to-sample accuracy in schizophrenia. *Psychiatry Research: Neuroimaging*, *193*(3), 138–143. doi: 10.1016/j.pscychresns.2011.03.008

Avsar, K. B., Weller, R. E., Cox, J. E., Reid, M. A., White, D. M., & Lahti, A. C. (2013). An fMRI investigation of delay discounting in patients with schizophrenia. *Brain and Behavior*, *3*(4), 384–401. doi: 10.1002/brb3.135

Backes, H., Dietsche, B., Nagels, A., Stratmann, M., Konrad, C., Kircher, T., & Krug, A. (2014). Increased neural activity during overt and continuous semantic verbal fluency in major depression: Mainly a failure to deactivate. *European Archives of Psychiatry and Clinical Neuroscience*, *264*(7), 631–645. doi: 10.1007/s00406-014-0491-y

Backes, V., Kellermann, T., Voss, B., Krämer, J., Depner, C., Schneider, F., & Habel, U. (2011). Neural correlates of the attention network test in schizophrenia. *European Archives of Psychiatry and Clinical Neuroscience*, *261*(S2), 155–160. doi: 10.1007/s00406-011-0264-9

Bak, N., Rostrup, E., Larsson, H. B. W., Glenthøj, B. Y., & Oranje, B. (2014). Concurrent functional magnetic resonance imaging and electroencephalography assessment of sensory gating in schizophrenia. *Human Brain Mapping*, *35*(8), 3578–3587. doi: 10.1002/hbm.22422

Bär, K.-J., Wagner, G., Koschke, M., Boettger, S., Boettger, M. K., Schlösser, R., & Sauer, H. (2007). Increased Prefrontal Activation During Pain Perception in Major Depression. *Biological Psychiatry*, *62*(11), 1281–1287. doi: 10.1016/j.biopsych.2007.02.011

Barch, D. M., & Csernansky, J. G. (2007). Abnormal Parietal Cortex Activation During Working Memory in Schizophrenia: Verbal Phonological Coding Disturbances Versus Domain-General Executive Dysfunction. *American Journal of Psychiatry*, *164*(7), 1090–1098. doi: 10.1176/ajp.2007.164.7.1090

Barkataki, I., Kumari, V., Das, M., Sumich, A., Taylor, P., & Sharma, T. (2008). Neural correlates of deficient response inhibition in mentally disordered violent individuals. *Behavioral Sciences & the Law*, *26*(1), 51–64. doi: 10.1002/bsl.787

Bartholomeusz, C. F., Ganella, E. P., Whittle, S., Allott, K., Thompson, A., Abu-Akel, A., … Wood, S. J. (2018). An fMRI study of theory of mind in individuals with first episode psychosis. *Psychiatry Research: Neuroimaging*, *281*, 1–11. doi: 10.1016/j.pscychresns.2018.08.011

Becerril, K., & Barch, D. (2011). Influence of Emotional Processing on Working Memory in Schizophrenia. *Schizophrenia Bulletin*, *37*(5), 1027–1038. doi: 10.1093/schbul/sbq009

Bedford, N. J., Surguladze, S., Giampietro, V., Brammer, M. J., & David, A. S. (2012). Self-evaluation in schizophrenia: An fMRI study with implications for the understanding of insight. *BMC Psychiatry*, *12*(1), 106. doi: 10.1186/1471-244X-12-106

Bender, J., Reuter, B., Möllers, D., Kaufmann, C., Gallinat, J., & Kathmann, N. (2013). Neural correlates of impaired volitional action control in schizophrenia patients: Neural correlates of volition in schizophrenia. *Psychophysiology*, *50*(9), 872–884. doi: 10.1111/psyp.12060

Bergé, D., Carmona, S., Salgado, P., Rovira, M., Bulbena, A., & Vilarroya, O. (2014). Limbic activity in antipsychotic naïve first-episode psychotic subjects during facial emotion discrimination. *European Archives of Psychiatry and Clinical Neuroscience*, *264*(4), 271–283. doi: 10.1007/s00406-013-0465-5

Berger, P., Bitsch, F., Nagels, A., Straube, B., & Falkenberg, I. (2018). Frontal hypoactivation and alterations in the reward-system during humor processing in patients with schizophrenia spectrum disorders. *Schizophrenia Research*, *202*, 149–157. doi: 10.1016/j.schres.2018.06.053

Bermpohl, F., Walter, M., Sajonz, B., Lücke, C., Hägele, C., Sterzer, P., … Northoff, G. (2009). Attentional modulation of emotional stimulus processing in patients with major depression—Alterations in prefrontal cortical regions. *Neuroscience Letters*, *463*(2), 108–113. doi: 10.1016/j.neulet.2009.07.061

Bertolino, A., Fazio, L., Caforio, G., Blasi, G., Rampino, A., Romano, R., … Sadee, W. (2009). Functional variants of the dopamine receptor D2 gene modulate prefronto-striatal phenotypes in schizophrenia. *Brain*, *132*(2), 417–425. doi: 10.1093/brain/awn248

Bjorkquist, O. A., & Herbener, E. S. (2013). Social perception in schizophrenia: Evidence of temporo-occipital and prefrontal dysfunction. *Psychiatry Research: Neuroimaging*, *212*(3), 175–182. doi: 10.1016/j.pscychresns.2012.12.002

Blasi, G., Taurisano, P., Papazacharias, A., Caforio, G., Romano, R., Lobianco, L., … Bertolino, A. (2010). Nonlinear Response of the Anterior Cingulate and Prefrontal Cortex in Schizophrenia as a Function of Variable Attentional Control. *Cerebral Cortex*, *20*(4), 837–845. doi: 10.1093/cercor/bhp146

Bonner-Jackson, A., Haut, K., Csernansky, J. G., & Barch, D. M. (2005). The Influence of Encoding Strategy on Episodic Memory and Cortical Activity in Schizophrenia. *Biological Psychiatry*, *58*(1), 47–55. doi: 10.1016/j.biopsych.2005.05.011

Bor, J., Brunelin, J., Sappey-Marinier, D., Ibarrola, D., d’Amato, T., Suaud-Chagny, M.-F., & Saoud, M. (2011). Thalamus abnormalities during working memory in schizophrenia. An fMRI study. *Schizophrenia Research*, *125*(1), 49–53. doi: 10.1016/j.schres.2010.10.018

Brahmbhatt, S. B., Haut, K., Csernansky, J. G., & Barch, D. M. (2006). Neural correlates of verbal and nonverbal working memory deficits in individuals with schizophrenia and their high-risk siblings. *Schizophrenia Research*, *87*(1–3), 191–204. doi: 10.1016/j.schres.2006.05.019

Brandt, C. L., Eichele, T., Melle, I., Sundet, K., Server, A., Agartz, I., … Andreassen, O. A. (2014). Working memory networks and activation patterns in schizophrenia and bipolar disorder: Comparison with healthy controls. *British Journal of Psychiatry*, *204*(4), 290–298. doi: 10.1192/bjp.bp.113.129254

Breukelaar, I. A., Erlinger, M., Harris, A., Boyce, P., Hazell, P., Grieve, S. M., … Korgaonkar, M. S. (2020). Investigating the neural basis of cognitive control dysfunction in mood disorders. *Bipolar Disorders*, *22*(3), 286–295. doi: 10.1111/bdi.12844

Briceño, E. M., Weisenbach, S. L., Rapport, L. J., Hazlett, K. E., Bieliauskas, L. A., Haase, B. D., … Langenecker, S. A. (2013). Shifted inferior frontal laterality in women with major depressive disorder is related to emotion-processing deficits. *Psychological Medicine*, *43*(7), 1433–1445. doi: 10.1017/S0033291712002176

Briend, F., Marzloff, V., Brazo, P., Lecardeur, L., Leroux, E., Razafimandimby, A., & Dollfus, S. (2019). Social cognition in schizophrenia: Validation of an ecological fMRI task. *Psychiatry Research: Neuroimaging*, *286*, 60–68. doi: 10.1016/j.pscychresns.2019.03.004

Brooks, J. O., Vizueta, N., Penfold, C., Townsend, J. D., Bookheimer, S. Y., & Altshuler, L. L. (2015). Prefrontal hypoactivation during working memory in bipolar II depression. *Psychological Medicine*, *45*(8), 1731–1740. doi: 10.1017/S0033291714002852

Broome, M. R., Matthiasson, P., Fusar-Poli, P., Woolley, J. B., Johns, L. C., Tabraham, P., … McGuire, P. K. (2009). Neural correlates of executive function and working memory in the ‘at-risk mental state.’ *British Journal of Psychiatry*, *194*(1), 25–33. doi: 10.1192/bjp.bp.107.046789

Brüne, M., Lissek, S., Fuchs, N., Witthaus, H., Peters, S., Nicolas, V., … Tegenthoff, M. (2008). An fMRI study of theory of mind in schizophrenic patients with “passivity” symptoms. *Neuropsychologia*, *46*(7), 1992–2001. doi: 10.1016/j.neuropsychologia.2008.01.023

Brüne, M., Özgürdal, S., Ansorge, N., von Reventlow, H. G., Peters, S., Nicolas, V., … Lissek, S. (2011). An fMRI study of “theory of mind” in at-risk states of psychosis: Comparison with manifest schizophrenia and healthy controls. *NeuroImage*, *55*(1), 329–337. doi: 10.1016/j.neuroimage.2010.12.018

Burrows, K., Stewart, J. L., Kuplicki, R., Figueroa-Hall, L., Spechler, P. A., Zheng, H., … Paulus, M. P. (2021). Elevated peripheral inflammation is associated with attenuated striatal reward anticipation in major depressive disorder. *Brain, Behavior, and Immunity*, *93*, 214–225. doi: 10.1016/j.bbi.2021.01.016

Cadena, E. J., White, D. M., Kraguljac, N. V., Reid, M. A., & Lahti, A. C. (2018). Evaluation of fronto-striatal networks during cognitive control in unmedicated patients with schizophrenia and the effect of antipsychotic medication. *Npj Schizophrenia*, *4*(1), 8. doi: 10.1038/s41537-018-0051-y

Callicott, J. H. (2000). Physiological Dysfunction of the Dorsolateral Prefrontal Cortex in Schizophrenia Revisited. *Cerebral Cortex*, *10*(11), 1078–1092. doi: 10.1093/cercor/10.11.1078

Callicott, Joseph H., Mattay, V. S., Verchinski, B. A., Marenco, S., Egan, M. F., & Weinberger, D. R. (2003). Complexity of Prefrontal Cortical Dysfunction in Schizophrenia: More Than Up or Down. *American Journal of Psychiatry*, *160*(12), 2209–2215. doi: 10.1176/appi.ajp.160.12.2209

Camchong, J., Dyckman, K. A., Austin, B. P., Clementz, B. A., & McDowell, J. E. (2008). Common Neural Circuitry Supporting Volitional Saccades and Its Disruption in Schizophrenia Patients and Relatives. *Biological Psychiatry*, *64*(12), 1042–1050. doi: 10.1016/j.biopsych.2008.06.015

Canli, T., Sivers, H., Thomason, M. E., Whitfield-Gabrieli, S., Gabrieli, J. D. E., & Gotlib, I. H. (2004). Brain activation to emotional words in depressed vs healthy subjects: *NeuroReport*, *15*(17), 2585–2588. doi: 10.1097/00001756-200412030-00005

Caseras, X., Lawrence, N. S., Murphy, K., Wise, R. G., & Phillips, M. L. (2013). Ventral Striatum Activity in Response to Reward: Differences Between Bipolar I and II Disorders. *American Journal of Psychiatry*, *170*(5), 533–541. doi: 10.1176/appi.ajp.2012.12020169

Cerullo, M. A., Eliassen, J. C., Smith, C. T., Fleck, D. E., Nelson, E. B., Strawn, J. R., … Strakowski, S. M. (2014). Bipolar I disorder and major depressive disorder show similar brain activation during depression. *Bipolar Disorders*, *16*(7), 703–712. doi: 10.1111/bdi.12225

Chandrasekhar Pammi, V. S., Pillai Geethabhavan Rajesh, P., Kesavadas, C., Rappai Mary, P., Seema, S., Radhakrishnan, A., & Sitaram, R. (2015). Neural loss aversion differences between depression patients and healthy individuals: A functional MRI investigation. *The Neuroradiology Journal*, *28*(2), 97–105. doi: 10.1177/1971400915576670

Chang, K., Adleman, N. E., Dienes, K., Simeonova, D. I., Menon, V., & Reiss, A. (2004). Anomalous Prefrontal-Subcortical Activation in Familial Pediatric BipolarDisorder: A Functional Magnetic Resonance Imaging Investigation. *Archives of General Psychiatry*, *61*(8), 781. doi: 10.1001/archpsyc.61.8.781

Chase, H. W., Nusslock, R., Almeida, J. R., Forbes, E. E., LaBarbara, E. J., & Phillips, M. L. (2013). Dissociable patterns of abnormal frontal cortical activation during anticipation of an uncertain reward or loss in bipolar versus major depression. *Bipolar Disorders*, *15*(8), 839–854. doi: 10.1111/bdi.12132

Chechko, N., Augustin, M., Zvyagintsev, M., Schneider, F., Habel, U., & Kellermann, T. (2013). Brain circuitries involved in emotional interference task in major depression disorder. *Journal of Affective Disorders*, *149*(1–3), 136–145. doi: 10.1016/j.jad.2013.01.013

Chen, C.-H., Lennox, B., Jacob, R., Calder, A., Lupson, V., Bisbrown-Chippendale, R., … Bullmore, E. (2006). Explicit and Implicit Facial Affect Recognition in Manic and Depressed States of Bipolar Disorder: A Functional Magnetic Resonance Imaging Study. *Biological Psychiatry*, *59*(1), 31–39. doi: 10.1016/j.biopsych.2005.06.008

Chen, P.-J., Fan, L.-Y., Hwang, T.-J., Hwu, H.-G., Liu, C.-M., & Chou, T.-L. (2013). The deficits on a cortical–subcortical loop of meaning processing in schizophrenia. *NeuroReport*, *24*(3), 147–151. doi: 10.1097/WNR.0b013e32835df562

Chen, X., Wang, Y., Wang, Y., Yang, T., Zou, L., Huang, J., … Chan, R. C. K. (2016). Neural correlates of prospective memory impairments in schizophrenia. *Neuropsychology*, *30*(2), 169–180. doi: 10.1037/neu0000225

Choi, J. W., Jeong, B. S., & Kim, J.-W. (2008). Dysfunction of the Left Dorsolateral Prefrontal Cortex is Primarily Responsible for Impaired Attentional Processing in Schizophrenia. *Psychiatry Investigation*, *5*(1), 52. doi: 10.4306/pi.2008.5.1.52

Choi, J.-S., Park, J.-Y., Jung, M. H., Jang, J. H., Kang, D.-H., Jung, W. H., … Kwon, J. S. (2012). Phase-Specific Brain Change of Spatial Working Memory Processing in Genetic and Ultra-High Risk Groups of Schizophrenia. *Schizophrenia Bulletin*, *38*(6), 1189–1199. doi: 10.1093/schbul/sbr038

Choi, S.-H., Lee, S.-H., Park, H.-J., Chun, J. W., Kang, J. I., & Kim, J.-J. (2013). Perceived patient–parent relationships and neural representation of parents in schizophrenia. *European Archives of Psychiatry and Clinical Neuroscience*, *263*(3), 259–269. doi: 10.1007/s00406-012-0334-7

Choudhury, M., Steines, M., Nagels, A., Riedl, L., Kircher, T., & Straube, B. (2021). Neural Basis of Speech-Gesture Mismatch Detection in Schizophrenia Spectrum Disorders. *Schizophrenia Bulletin*, *47*(6), 1761–1771. doi: 10.1093/schbul/sbab059

Chung, Y. S., & Barch, D. M. (2016). Frontal-striatum dysfunction during reward processing: Relationships to amotivation in schizophrenia. *Journal of Abnormal Psychology*, *125*(3), 453–469. doi: 10.1037/abn0000137

Ciaramidaro, A., Bölte, S., Schlitt, S., Hainz, D., Poustka, F., Weber, B., … Walter, H. (2018). Transdiagnostic deviant facial recognition for implicit negative emotion in autism and schizophrenia. *European Neuropsychopharmacology*, *28*(2), 264–275. doi: 10.1016/j.euroneuro.2017.12.005

Cieslik, E. C., Müller, V. I., Kellermann, T. S., Grefkes, C., Halfter, S., & Eickhoff, S. B. (2015). Shifted neuronal balance during stimulus–response integration in schizophrenia: An fMRI study. *Brain Structure and Function*, *220*(1), 249–261. doi: 10.1007/s00429-013-0652-1

Collier, A. K., Wolf, D. H., Valdez, J. N., Turetsky, B. I., Elliott, M. A., Gur, R. E., & Gur, R. C. (2014). Comparison of auditory and visual oddball fMRI in schizophrenia. *Schizophrenia Research*, *158*(1–3), 183–188. doi: 10.1016/j.schres.2014.06.019

Cooney, R. E., Joormann, J., Eugène, F., Dennis, E. L., & Gotlib, I. H. (2010). Neural correlates of rumination in depression. *Cognitive, Affective, & Behavioral Neuroscience*, *10*(4), 470–478. doi: 10.3758/CABN.10.4.470

Costafreda, S. G., Fu, C. H., Picchioni, M., Toulopoulou, T., McDonald, C., Kravariti, E., … McGuire, P. K. (2011). Pattern of neural responses to verbal fluency shows diagnostic specificity for schizophrenia and bipolar disorder. *BMC Psychiatry*, *11*(1), 18. doi: 10.1186/1471-244X-11-18

Creyaufmüller, M., Heim, S., Habel, U., & Mühlhaus, J. (2020). The influence of semantic associations on sentence production in schizophrenia: An fMRI study. *European Archives of Psychiatry and Clinical Neuroscience*, *270*(3), 359–372. doi: 10.1007/s00406-018-0936-9

Cuervo-Lombard, C., Lemogne, C., Gierski, F., Béra-Potelle, C., Tran, E., Portefaix, C., … Limosin, F. (2012). Neural basis of autobiographical memory retrieval in schizophrenia. *British Journal of Psychiatry*, *201*(6), 473–480. doi: 10.1192/bjp.bp.111.099820

Culbreth, A. J., Gold, J. M., Cools, R., & Barch, D. M. (2016). Impaired Activation in Cognitive Control Regions Predicts Reversal Learning in Schizophrenia. *Schizophrenia Bulletin*, *42*(2), 484–493. doi: 10.1093/schbul/sbv075

Curtis, V. A., Dixon, T. A., Morris, R. G., Bullmore, E. T., Brammer, M. J., Williams, S. C. R., … McGuire, P. K. (2001). Differential frontal activation in schizophrenia and bipolar illness during verbal fluency. *Journal of Affective Disorders*, *66*(2–3), 111–121. doi: 10.1016/S0165-0327(00)00240-8

Dai, Q., Yin, X., Li, H., & Feng, Z. (2018). Orbito-frontal cortex mechanism of inhibition of return in current and remitted depression. *Human Brain Mapping*, *39*(7), 2941–2954. doi: 10.1002/hbm.24051

Dar, S., Liebenthal, E., Pan, H., Smith, T., Savitz, A., Landa, Y., … Stern, E. (2021). Abnormal semantic processing of threat words associated with excitement and hostility symptoms in schizophrenia. *Schizophrenia Research*, *228*, 394–402. doi: 10.1016/j.schres.2020.12.022

Das, P., Lagopoulos, J., Coulston, C. M., Henderson, A. F., & Malhi, G. S. (2012). Mentalizing impairment in schizophrenia: A functional MRI study. *Schizophrenia Research*, *134*(2–3), 158–164. doi: 10.1016/j.schres.2011.08.019

Davalos, D. B., Rojas, D. C., & Tregellas, J. R. (2011). Temporal processing in schizophrenia: Effects of task-difficulty on behavioral discrimination and neuronal responses. *Schizophrenia Research*, *127*(1–3), 123–130. doi: 10.1016/j.schres.2010.06.020

Davis, E. G., Foland-Ross, L. C., & Gotlib, I. H. (2018). Neural correlates of top-down regulation and generation of negative affect in major depressive disorder. *Psychiatry Research: Neuroimaging*, *276*, 1–8. doi: 10.1016/j.pscychresns.2018.04.001

De Coster, L., Lin, L., Mathalon, D. H., & Woolley, J. D. (2019). Neural and behavioral effects of oxytocin administration during theory of mind in schizophrenia and controls: A randomized control trial. *Neuropsychopharmacology*, *44*(11), 1925–1931. doi: 10.1038/s41386-019-0417-5

de la Fuente-Sandoval, C., Favila, R., Gómez-Martin, D., Pellicer, F., & Graff-Guerrero, A. (2010). Functional magnetic resonance imaging response to experimental pain in drug-free patients with schizophrenia. *Psychiatry Research: Neuroimaging*, *183*(2), 99–104. doi: 10.1016/j.pscychresns.2010.05.003

De La Peña-Arteaga, V., Berruga-Sánchez, M., Steward, T., Martínez-Zalacaín, I., Goldberg, X., Wainsztein, A., … Soriano-Mas, C. (2021). An fMRI study of cognitive reappraisal in major depressive disorder and borderline personality disorder. *European Psychiatry*, *64*(1), e56. doi: 10.1192/j.eurpsy.2021.2231

Deckersbach, T., Rauch, S. L., Buhlmann, U., Ostacher, M. J., Beucke, J.-C., Nierenberg, A. A., … Dougherty, D. D. (2008). An fMRI investigation of working memory and sadness in females with bipolar disorder: A brief report. *Bipolar Disorders*, *10*(8), 928–942. doi: 10.1111/j.1399-5618.2008.00633.x

Demenescu, L. R., Renken, R., Kortekaas, R., Van Tol, M.-J., Marsman, J. B. C., Van Buchem, M. A., … Aleman, A. (2011). Neural correlates of perception of emotional facial expressions in out-patients with mild-to-moderate depression and anxiety. A multicenter fMRI study. *Psychological Medicine*, *41*(11), 2253–2264. doi: 10.1017/S0033291711000596

Derntl, B., Finkelmeyer, A., Voss, B., Eickhoff, S. B., Kellermann, T., Schneider, F., & Habel, U. (2012). Neural correlates of the core facets of empathy in schizophrenia. *Schizophrenia Research*, *136*(1–3), 70–81. doi: 10.1016/j.schres.2011.12.018

Derntl, B., Seidel, E.-M., Eickhoff, S. B., Kellermann, T., Gur, R. C., Schneider, F., & Habel, U. (2011). Neural correlates of social approach and withdrawal in patients with major depression. *Social Neuroscience*, *6*(5–6), 482–501. doi: 10.1080/17470919.2011.579800

Deserno, L., Boehme, R., Mathys, C., Katthagen, T., Kaminski, J., Stephan, K. E., … Schlagenhauf, F. (2020). Volatility Estimates Increase Choice Switching and Relate to Prefrontal Activity in Schizophrenia. *Biological Psychiatry: Cognitive Neuroscience and Neuroimaging*, *5*(2), 173–183. doi: 10.1016/j.bpsc.2019.10.007

DeVille, D. C., Kerr, K. L., Avery, J. A., Burrows, K., Bodurka, J., Feinstein, J. S., … Simmons, W. K. (2018). The Neural Bases of Interoceptive Encoding and Recall in Healthy Adults and Adults With Depression. *Biological Psychiatry: Cognitive Neuroscience and Neuroimaging*, *3*(6), 546–554. doi: 10.1016/j.bpsc.2018.03.010

Dichter, G. S., Felder, J. N., & Smoski, M. J. (2009). Affective context interferes with cognitive control in unipolar depression: An fMRI investigation. *Journal of Affective Disorders*, *114*(1–3), 131–142. doi: 10.1016/j.jad.2008.06.027

Dichter, G. S., Kozink, R. V., McClernon, F. J., & Smoski, M. J. (2012). Remitted major depression is characterized by reward network hyperactivation during reward anticipation and hypoactivation during reward outcomes. *Journal of Affective Disorders*, *136*(3), 1126–1134. doi: 10.1016/j.jad.2011.09.048

Dickstein, D. P., Rich, B. A., Roberson-Nay, R., Berghorst, L., Vinton, D., Pine, D. S., & Leibenluft, E. (2007). Neural activation during encoding of emotional faces in pediatric bipolar disorder. *Bipolar Disorders*, *9*(7), 679–692. doi: 10.1111/j.1399-5618.2007.00418.x

Dietsche, B., Backes, H., Stratmann, M., Konrad, C., Kircher, T., & Krug, A. (2014). Altered neural function during episodic memory encoding and retrieval in major depression: Neural Function During Episodic Memory in MDD. *Human Brain Mapping*, *35*(9), 4293–4302. doi: 10.1002/hbm.22475

Dima, D., De Jong, S., Breen, G., & Frangou, S. (2016). The polygenic risk for bipolar disorder influences brain regional function relating to visual and default state processing of emotional information. *NeuroImage: Clinical*, *12*, 838–844. doi: 10.1016/j.nicl.2016.10.022

Dodell-Feder, D., Tully, L. M., Lincoln, S. H., & Hooker, C. I. (2014). The neural basis of theory of mind and its relationship to social functioning and social anhedonia in individuals with schizophrenia. *NeuroImage: Clinical*, *4*, 154–163. doi: 10.1016/j.nicl.2013.11.006

Dong, D., Belleau, E. L., Ironside, M., Zhong, X., Sun, X., Xiong, G., … Pizzagalli, D. A. (2022). Distinct stress‐related medial prefrontal cortex activation in women with depression with and without childhood maltreatment. *Depression and Anxiety*, *39*(4), 296–306. doi: 10.1002/da.23243

Dowd, E. C., & Barch, D. M. (2012). Pavlovian Reward Prediction and Receipt in Schizophrenia: Relationship to Anhedonia. *PLoS ONE*, *7*(5), e35622. doi: 10.1371/journal.pone.0035622

Dowd, E. C., Frank, M. J., Collins, A., Gold, J. M., & Barch, D. M. (2016). Probabilistic Reinforcement Learning in Patients With Schizophrenia: Relationships to Anhedonia and Avolition. *Biological Psychiatry. Cognitive Neuroscience and Neuroimaging*, *1*(5), 460–473. doi: 10.1016/j.bpsc.2016.05.005

Drapier, D., Surguladze, S., Marshall, N., Schulze, K., Fern, A., Hall, M.-H., … McDonald, C. (2008). Genetic Liability for Bipolar Disorder Is Characterized by Excess Frontal Activation in Response to a Working Memory Task. *Biological Psychiatry*, *64*(6), 513–520. doi: 10.1016/j.biopsych.2008.04.038

Dukart, J., Holiga, S., Rullmann, M., Lanzenberger, R., Hawkins, P. C. T., Mehta, M. A., … Eickhoff, S. B. (2021). JuSpace: A tool for spatial correlation analyses of magnetic resonance imaging data with nuclear imaging derived neurotransmitter maps. *Human Brain Mapping*, *42*(3), 555–566. doi: 10.1002/hbm.25244

Dutra, S. J., Cunningham, W. A., Kober, H., & Gruber, J. (2015). Elevated striatal reactivity across monetary and social rewards in bipolar I disorder. *Journal of Abnormal Psychology*, *124*(4), 890–904. doi: 10.1037/abn0000092

Dyck, M., Loughead, J., Gur, R. C., Schneider, F., & Mathiak, K. (2014). Hyperactivation balances sensory processing deficits during mood induction in schizophrenia. *Social Cognitive and Affective Neuroscience*, *9*(2), 167–175. doi: 10.1093/scan/nss120

Dyckman, K. A., Lee, A. K. C., Agam, Y., Vangel, M., Goff, D. C., Barton, J. J. S., & Manoach, D. S. (2011). Abnormally persistent fMRI activation during antisaccades in schizophrenia: A neural correlate of perseveration? *Schizophrenia Research*, *132*(1), 62–68. doi: 10.1016/j.schres.2011.07.026

Dzafic, I., Burianová, H., Martin, A. K., & Mowry, B. (2018). Neural correlates of dynamic emotion perception in schizophrenia and the influence of prior expectations. *Schizophrenia Research*, *202*, 129–137. doi: 10.1016/j.schres.2018.06.015

Eich, T. S., Nee, D. E., Insel, C., Malapani, C., & Smith, E. E. (2014). Neural Correlates of Impaired Cognitive Control over Working Memory in Schizophrenia. *Biological Psychiatry*, *76*(2), 146–153. doi: 10.1016/j.biopsych.2013.09.032

Elliott, R., Ogilvie, A., Rubinsztein, J. S., Calderon, G., Dolan, R. J., & Sahakian, B. J. (2004). Abnormal ventral frontal response during performance of an affective go/no go task in patients with mania. *Biological Psychiatry*, *55*(12), 1163–1170. doi: 10.1016/j.biopsych.2004.03.007

Elliott, R., Rubinsztein, J. S., Sahakian, B. J., & Dolan, R. J. (2002). The Neural Basis of Mood-Congruent Processing Biases in Depression. *Archives of General Psychiatry*, *59*(7), 597. doi: 10.1001/archpsyc.59.7.597

Epstein, J., Pan, H., Kocsis, J. H., Yang, Y., Butler, T., Chusid, J., … Silbersweig, D. A. (2006). Lack of Ventral Striatal Response to Positive Stimuli in Depressed Versus Normal Subjects. *American Journal of Psychiatry*, *163*(10), 1784–1790. doi: 10.1176/ajp.2006.163.10.1784

Eryilmaz, H., Tanner, A. S., Ho, N. F., Nitenson, A. Z., Silverstein, N. J., Petruzzi, L. J., … Roffman, J. L. (2016). Disrupted Working Memory Circuitry in Schizophrenia: Disentangling fMRI Markers of Core Pathology vs Other Aspects of Impaired Performance. *Neuropsychopharmacology*, *41*(9), 2411–2420. doi: 10.1038/npp.2016.55

Ettinger, U., Williams, S. C. R., Fannon, D., Premkumar, P., Kuipers, E., Möller, H.-J., & Kumari, V. (2011). Functional magnetic resonance imaging of a parametric working memory task in schizophrenia: Relationship with performance and effects of antipsychotic treatment. *Psychopharmacology*, *216*(1), 17–27. doi: 10.1007/s00213-011-2214-7

Eyler, L. T., Jeste, D. V., & Brown, G. G. (2008). Brain response abnormalities during verbal learning among patients with schizophrenia. *Psychiatry Research: Neuroimaging*, *162*(1), 11–25. doi: 10.1016/j.pscychresns.2007.03.009

Eyler, L. T., Olsen, R. K., Jeste, D. V., & Brown, G. G. (2004). Abnormal brain response of chronic schizophrenia patients despite normal performance during a visual vigilance task. *Psychiatry Research: Neuroimaging*, *130*(3), 245–257. doi: 10.1016/j.pscychresns.2004.01.003

Fakra, E., Salgado-Pineda, P., Delaveau, P., Hariri, A. R., & Blin, O. (2008). Neural bases of different cognitive strategies for facial affect processing in schizophrenia. *Schizophrenia Research*, *100*(1–3), 191–205. doi: 10.1016/j.schres.2007.11.040

Fassbender, C., Scangos, K., Lesh, T. A., & Carter, C. S. (2014). RT distributional analysis of cognitive-control-related brain activity in first-episode schizophrenia. *Cognitive, Affective & Behavioral Neuroscience*, *14*(1), 175–188. doi: 10.3758/s13415-014-0252-4

Fatjó-Vilas, M., Pomarol-Clotet, E., Salvador, R., Monté, G. C., Gomar, J. J., Sarró, S., … Fañanás, L. (2012). Effect of the Interleukin-1β Gene on Dorsolateral Prefrontal Cortex Function in Schizophrenia: A Genetic Neuroimaging Study. *Biological Psychiatry*, *72*(9), 758–765. doi: 10.1016/j.biopsych.2012.04.035

Favre, P., Baciu, M., Pichat, C., De Pourtalès, M.-A., Fredembach, B., Garçon, S., … Polosan, M. (2013). Modulation of fronto-limbic activity by the psychoeducation in euthymic bipolar patients. A functional MRI study. *Psychiatry Research: Neuroimaging*, *214*(3), 285–295. doi: 10.1016/j.pscychresns.2013.07.007

Favre, P., Polosan, M., Pichat, C., Bougerol, T., & Baciu, M. (2015). Cerebral Correlates of Abnormal Emotion Conflict Processing in Euthymic Bipolar Patients: A Functional MRI Study. *PLOS ONE*, *10*(8), e0134961. doi: 10.1371/journal.pone.0134961

Fernández-Corcuera, P., Salvador, R., Monté, G. C., Salvador Sarró, S., Goikolea, J. M., Amann, B., … Pomarol-Clotet, E. (2013). Bipolar depressed patients show both failure to activate and failure to de-activate during performance of a working memory task. *Journal of Affective Disorders*, *148*(2–3), 170–178. doi: 10.1016/j.jad.2012.04.009

Ferri, F., Costantini, M., Salone, A., Ebisch, S., De Berardis, D., Mazzola, V., … Gallese, V. (2014). Binding Action and Emotion in First-Episode Schizophrenia. *Psychopathology*, *47*(6), 394–407. doi: 10.1159/000366133

Finlayson-Short, L., Harrison, B. J., & Davey, C. (2021). Self-other referential neural processing in social anxiety disorder and major depressive disorder. *NeuroImage: Clinical*, *30*, 102669. doi: 10.1016/j.nicl.2021.102669

Fitzgerald, P. B., Srithiran, A., Benitez, J., Daskalakis, Z. Z., Oxley, T. J., Kulkarni, J., & Egan, G. F. (2008). An fMRI study of prefrontal brain activation during multiple tasks in patients with major depressive disorder. *Human Brain Mapping*, *29*(4), 490–501. doi: 10.1002/hbm.20414

Fleck, D. E., Kotwal, R., Eliassen, J. C., Lamy, M., Delbello, M. P., Adler, C. M., … Strakowski, S. M. (2011). Preliminary evidence for increased frontosubcortical activation on a motor impulsivity task in mixed episode bipolar disorder. *Journal of Affective Disorders*, *133*(1–2), 333–339. doi: 10.1016/j.jad.2011.03.053

Foell, J., Klawohn, J., Bruchnak, A., Brush, C. J., Patrick, C. J., & Hajcak, G. (2021). Ventral striatal activation during reward differs between major depression with and without impaired mood reactivity. *Psychiatry Research: Neuroimaging*, *313*, 111298. doi: 10.1016/j.pscychresns.2021.111298

Foland, L. C., Altshuler, L. L., Bookheimer, S. Y., Eisenberger, N., Townsend, J., & Thompson, P. M. (2008). Evidence for deficient modulation of amygdala response by prefrontal cortex in bipolar mania. *Psychiatry Research: Neuroimaging*, *162*(1), 27–37. doi: 10.1016/j.pscychresns.2007.04.007

Foland-Ross, L. C., Bookheimer, S. Y., Lieberman, M. D., Sugar, C. A., Townsend, J. D., Fischer, J., … Altshuler, L. L. (2012). Normal amygdala activation but deficient ventrolateral prefrontal activation in adults with bipolar disorder during euthymia. *NeuroImage*, *59*(1), 738–744. doi: 10.1016/j.neuroimage.2011.07.054

Foucher, J. R., Luck, D., Marrer, C., Pham, B.-T., Gounot, D., Vidailhet, P., & Otzenberger, H. (2011). fMRI working memory hypo-activations in schizophrenia come with a coupling deficit between arousal and cognition. *Psychiatry Research: Neuroimaging*, *194*(1), 21–29. doi: 10.1016/j.pscychresns.2011.06.004

Fournier, J. C., Keener, M. T., Mullin, B. C., Hafeman, D. M., LaBarbara, E. J., Stiffler, R. S., … Phillips, M. L. (2013). Heterogeneity of amygdala response in major depressive disorder: The impact of lifetime subthreshold mania. *Psychological Medicine*, *43*(2), 293–302. doi: 10.1017/S0033291712000918

Francis, M. M., Hummer, T. A., Vohs, J. L., Yung, M. G., Liffick, E., Mehdiyoun, N. F., … Breier, A. (2016). Functional neuroanatomical correlates of episodic memory impairment in early phase psychosis. *Brain Imaging and Behavior*, *10*(1), 1–11. doi: 10.1007/s11682-015-9357-9

Frodl, T., Scheuerecker, J., Albrecht, J., Kleemann, A. M., Müller-Schunk, S., Koutsouleris, N., … Meisenzahl, E. (2009). Neuronal correlates of emotional processing in patients with major depression. *The World Journal of Biological Psychiatry*, *10*(3), 202–208. doi: 10.1080/15622970701624603

Fryer, S. L., Roach, B. J., Ford, J. M., Donaldson, K. R., Calhoun, V. D., Pearlson, G. D., … Mathalon, D. H. (2019). Should I Stay or Should I Go? FMRI Study of Response Inhibition in Early Illness Schizophrenia and Risk for Psychosis. *Schizophrenia Bulletin*, *45*(1), 158–168. doi: 10.1093/schbul/sbx198

Fu, C. H. Y., Suckling, J., Williams, S. C. R., Andrew, C. M., Vythelingum, G. N., & McGuire, P. K. (2005). Effects of Psychotic State and Task Demand on Prefrontal Function in Schizophrenia: An fMRI Study of Overt Verbal Fluency. *American Journal of Psychiatry*, *162*(3), 485–494. doi: 10.1176/appi.ajp.162.3.485

Fu, C. H. Y., Williams, S. C. R., Brammer, M. J., Suckling, J., Kim, J., Cleare, A. J., … Bullmore, E. T. (2007). Neural Responses to Happy Facial Expressions in Major Depression Following Antidepressant Treatment. *American Journal of Psychiatry*, *164*(4), 599–607. doi: 10.1176/ajp.2007.164.4.599

Fu, C. H. Y., Williams, S. C. R., Cleare, A. J., Brammer, M. J., Walsh, N. D., Kim, J., … Bullmore, E. T. (2004). Attenuation of the Neural Response to Sad Faces in Major Depressionby Antidepressant Treatment: A Prospective, Event-Related Functional Magnetic Resonance ImagingStudy. *Archives of General Psychiatry*, *61*(9), 877. doi: 10.1001/archpsyc.61.9.877

Fuentes-Claramonte, P., López-Araquistain, L., Sarró, S., Sans-Sansa, B., Ortiz-Gil, J., Maristany, T., … Pomarol-Clotet, E. (2021). Brain functional correlates of formal thought disorder in schizophrenia: Examining the frontal/dysexecutive hypothesis. *Psychological Medicine*, *51*(14), 2446–2453. doi: 10.1017/S0033291720001063

Fuentes-Claramonte, Paola, Martin-Subero, M., Salgado-Pineda, P., Santo-Angles, A., Argila-Plaza, I., Salavert, J., … Salvador, R. (2020). Brain imaging correlates of self- and other-reflection in schizophrenia. *NeuroImage: Clinical*, *25*, 102134. doi: 10.1016/j.nicl.2019.102134

Furuichi, A., Kawasaki, Y., Takahashi, T., Nakamura, K., Tanino, R., Noguchi, K., … Suzuki, M. (2019). Altered neural basis of self-reflective processing in schizophrenia: An fMRI study. *Asian Journal of Psychiatry*, *45*, 53–60. doi: 10.1016/j.ajp.2019.08.007

Gaebler, A. J., Mathiak, K., Koten, J. W., König, A. A., Koush, Y., Weyer, D., … Zvyagintsev, M. (2015). Auditory mismatch impairments are characterized by core neural dysfunctions in schizophrenia. *Brain*, *138*(5), 1410–1423. doi: 10.1093/brain/awv049

Gao, Z., Zhao, W., Liu, S., Liu, Z., Yang, C., & Xu, Y. (2021). Facial Emotion Recognition in Schizophrenia. *Frontiers in Psychiatry*, *12*, 633717. doi: 10.3389/fpsyt.2021.633717

Garcia-Leon, M. A., Fuentes-Claramonte, P., Valiente-Gómez, A., Natividad, C., Salgado-Pineda, P., Gomar, J. J., … Pomarol-Clotet, E. (2021). Altered brain responses to specific negative emotions in schizophrenia. *NeuroImage: Clinical*, *32*, 102894. doi: 10.1016/j.nicl.2021.102894

García-Martí, G. (2012). Multimodal morphometry and functional magnetic resonance imaging in schizophrenia and auditory hallucinations. *World Journal of Radiology*, *4*(4), 159. doi: 10.4329/wjr.v4.i4.159

Garrett, A., Kelly, R., Gomez, R., Keller, J., Schatzberg, A. F., & Reiss, A. L. (2011). Aberrant Brain Activation During a Working Memory Task in Psychotic Major Depression. *American Journal of Psychiatry*, *168*(2), 173–182. doi: 10.1176/appi.ajp.2010.09121718

Garrison, J. R., Fernandez-Egea, E., Zaman, R., Agius, M., & Simons, J. S. (2017). Reality monitoring impairment in schizophrenia reflects specific prefrontal cortex dysfunction. *NeuroImage: Clinical*, *14*, 260–268. doi: 10.1016/j.nicl.2017.01.028

Gärtner, M., Ghisu, M. E., Scheidegger, M., Bönke, L., Fan, Y., Stippl, A., … Grimm, S. (2018). Aberrant working memory processing in major depression: Evidence from multivoxel pattern classification. *Neuropsychopharmacology*, *43*(9), 1972–1979. doi: 10.1038/s41386-018-0081-1

Gawne, T. J., Overbeek, G. J., Killen, J. F., Reid, M. A., Kraguljac, N. V., Denney, T. S., … Lahti, A. C. (2020). A multimodal magnetoencephalography 7 T fMRI and 7 T proton MR spectroscopy study in first episode psychosis. *Npj Schizophrenia*, *6*(1), 23. doi: 10.1038/s41537-020-00113-4

Genzel, L., Dresler, M., Cornu, M., Jäger, E., Konrad, B., Adamczyk, M., … Goya-Maldonado, R. (2015). Medial Prefrontal-Hippocampal Connectivity and Motor Memory Consolidation in Depression and Schizophrenia. *Biological Psychiatry*, *77*(2), 177–186. doi: 10.1016/j.biopsych.2014.06.004

Gizewski, E. R., Müller, B. W., Scherbaum, N., Lieb, B., Forsting, M., Wiltfang, J., … Schiffer, B. (2013). The impact of alcohol dependence on social brain function: Addiction and the social brain. *Addiction Biology*, *18*(1), 109–120. doi: 10.1111/j.1369-1600.2012.00437.x

Glahn, D. C., Robinson, J. L., Tordesillas-Gutierrez, D., Monkul, E. S., Holmes, M. K., Green, M. J., & Bearden, C. E. (2010). Fronto-temporal dysregulation in asymptomatic bipolar I patients: A paired associate functional MRI study. *Human Brain Mapping*, *31*(7), 1041–1051. doi: 10.1002/hbm.20918

Goikolea, J. M., Dima, D., Landín-Romero, R., Torres, I., DelVecchio, G., Valentí, M., … Vieta, E. (2019). Multimodal Brain Changes in First-Episode Mania: A Voxel-Based Morphometry, Functional Magnetic Resonance Imaging, and Connectivity Study. *Schizophrenia Bulletin*, *45*(2), 464–473. doi: 10.1093/schbul/sby047

González-Vivas, C., García-Martí, G., Soldevila-Matías, P., Sanz-Requena, R., Aguilar, E. J., Castro-Bleda, M. J., … Sanjuan, J. (2020). First-Episode Psychotic Patients Showed Longitudinal Brain Changes Using fMRI With an Emotional Auditory Paradigm. *Frontiers in Psychiatry*, *11*, 593042. doi: 10.3389/fpsyt.2020.593042

Goodin, P., Lamp, G., Hughes, M. E., Rossell, S. L., & Ciorciari, J. (2019). Decreased Response to Positive Facial Affect in a Depressed Cohort in the Dorsal Striatum During a Working Memory Task—A Preliminary fMRI Study. *Frontiers in Psychiatry*, *10*, 60. doi: 10.3389/fpsyt.2019.00060

Gotlib, I. H., Sivers, H., Gabrieli, J. D. E., Whitfield-Gabrieli, S., Goldin, P., Minor, K. L., & Canli, T. (2005). Subgenual anterior cingulate activation to valenced emotional stimuli in major depression. *NeuroReport*, *16*(16), 1731–1734. doi: 10.1097/01.wnr.0000183901.70030.82

Gradin, V. B., Waiter, G., Kumar, P., Stickle, C., Milders, M., Matthews, K., … Steele, J. D. (2012). Abnormal Neural Responses to Social Exclusion in Schizophrenia. *PLoS ONE*, *7*(8), e42608. doi: 10.1371/journal.pone.0042608

Gradin, V. B., Waiter, G., O’Connor, A., Romaniuk, L., Stickle, C., Matthews, K., … Douglas Steele, J. (2013). Salience network-midbrain dysconnectivity and blunted reward signals in schizophrenia. *Psychiatry Research: Neuroimaging*, *211*(2), 104–111. doi: 10.1016/j.pscychresns.2012.06.003

Grant, K., Hassel, S., Bobyn, J. A., Hall, G. B. C., & MacQueen, G. M. (2018). A novel task for examining the neural basis of Theory of Mind deficits in bipolar disorder. *Psychiatry Research: Neuroimaging*, *282*, 143–150. doi: 10.1016/j.pscychresns.2018.06.001

Greening, S. G., Osuch, E. A., Williamson, P. C., & Mitchell, D. G. V. (2013). Emotion-related brain activity to conflicting socio-emotional cues in unmedicated depression. *Journal of Affective Disorders*, *150*(3), 1136–1141. doi: 10.1016/j.jad.2013.05.053

Greening, S. G., Osuch, E. A., Williamson, P. C., & Mitchell, D. G. V. (2014). The neural correlates of regulating positive and negative emotions in medication-free major depression. *Social Cognitive and Affective Neuroscience*, *9*(5), 628–637. doi: 10.1093/scan/nst027

Griego, J. A., Cortes, C. R., Nune, S., Fisher, J. E., & Tagamets, M.-A. (2008). Word and letter string processing networks in schizophrenia: Evidence for anomalies and compensation. *Brain and Language*, *107*(2), 158–166. doi: 10.1016/j.bandl.2008.04.001

Grimm, S., Boesiger, P., Beck, J., Schuepbach, D., Bermpohl, F., Walter, M., … Northoff, G. (2009). Altered Negative BOLD Responses in the Default-Mode Network during Emotion Processing in Depressed Subjects. *Neuropsychopharmacology*, *34*(4), 932–943. doi: 10.1038/npp.2008.81

Grot, S., Légaré, V. P., Lipp, O., Soulières, I., Dolcos, F., & Luck, D. (2017). Abnormal prefrontal and parietal activity linked to deficient active binding in working memory in schizophrenia. *Schizophrenia Research*, *188*, 68–74. doi: 10.1016/j.schres.2017.01.021

Groves, S. J., Pitcher, T. L., Melzer, T. R., Jordan, J., Carter, J. D., Malhi, G. S., … Porter, R. J. (2018). Brain activation during processing of genuine facial emotion in depression: Preliminary findings. *Journal of Affective Disorders*, *225*, 91–96. doi: 10.1016/j.jad.2017.07.049

Gruber, O., Tost, H., Henseler, I., Schmael, C., Scherk, H., Ende, G., … Rietschel, M. (2009). Pathological amygdala activation during working memory performance: Evidence for a pathophysiological trait marker in bipolar affective disorder. *Human Brain Mapping*, NA-NA. doi: 10.1002/hbm.20849

Guimond, S., Hawco, C., & Lepage, M. (2017). Prefrontal activity and impaired memory encoding strategies in schizophrenia. *Journal of Psychiatric Research*, *91*, 64–73. doi: 10.1016/j.jpsychires.2017.02.024

Guimond, S., Lepage, M., Benoit, A., Charbonneau, G., Hawco, C., Malla, A. K., … Brodeur, M. B. (2016). Recollection rejection of new items in individuals with first-episode psychosis. *Journal of Abnormal Psychology*, *125*(1), 104–113. doi: 10.1037/abn0000102

Guimond, S., Padani, S., Lutz, O., Eack, S., Thermenos, H., & Keshavan, M. (2018). Impaired regulation of emotional distractors during working memory load in schizophrenia. *Journal of Psychiatric Research*, *101*, 14–20. doi: 10.1016/j.jpsychires.2018.02.028

Gur, R. E., Turetsky, B. I., Loughead, J., Snyder, W., Kohler, C., Elliott, M., … Gur, R. C. (2007). Visual Attention Circuitry in Schizophrenia Investigated With Oddball Event-Related Functional Magnetic Resonance Imaging. *American Journal of Psychiatry*, *164*(3), 442–449. doi: 10.1176/ajp.2007.164.3.442

Gurler, D., White, D. M., Kraguljac, N. V., Ver Hoef, L., Martin, C., Tennant, B., & Lahti, A. C. (2021). Neural Signatures of Memory Encoding in Schizophrenia Are Modulated by Antipsychotic Treatment. *Neuropsychobiology*, *80*(1), 12–24. doi: 10.1159/000506402

Habel, U., Chechko, N., Pauly, K., Koch, K., Backes, V., Seiferth, N., … Kellermann, T. (2010). Neural correlates of emotion recognition in schizophrenia. *Schizophrenia Research*, *122*(1–3), 113–123. doi: 10.1016/j.schres.2010.06.009

Habel, U., Pauly, K., Koch, K., Kellermann, T., Reske, M., Backes, V., … Schneider, F. (2010). Emotion–cognition interactions in schizophrenia. *The World Journal of Biological Psychiatry*, *11*(8), 934–944. doi: 10.3109/15622975.2010.501820

Hahn, B., Robinson, B. M., Leonard, C. J., Luck, S. J., & Gold, J. M. (2018). Posterior Parietal Cortex Dysfunction Is Central to Working Memory Storage and Broad Cognitive Deficits in Schizophrenia. *The Journal of Neuroscience*, *38*(39), 8378–8387. doi: 10.1523/JNEUROSCI.0913-18.2018

Hahn, W., Domahs, F., Straube, B., Kircher, T., & Nagels, A. (2021). Neural processing of nouns and verbs in spontaneous speech of patients with schizophrenia. *Psychiatry Research: Neuroimaging*, *318*, 111395. doi: 10.1016/j.pscychresns.2021.111395

Hall, J., Whalley, H. C., Marwick, K., McKirdy, J., Sussmann, J., Romaniuk, L., … Lawrie, S. M. (2010). Hippocampal function in schizophrenia and bipolar disorder. *Psychological Medicine*, *40*(5), 761–770. doi: 10.1017/S0033291709991000

Hall, Jeremy, Whalley, H. C., McKirdy, J. W., Romaniuk, L., McGonigle, D., McIntosh, A. M., … Lawrie, S. M. (2008). Overactivation of Fear Systems to Neutral Faces in Schizophrenia. *Biological Psychiatry*, *64*(1), 70–73. doi: 10.1016/j.biopsych.2007.12.014

Hamilton, L. S., Altshuler, L. L., Townsend, J., Bookheimer, S. Y., Phillips, O. R., Fischer, J., … Narr, K. L. (2009). Alterations in functional activation in euthymic bipolar disorder and schizophrenia during a working memory task. *Human Brain Mapping*, *30*(12), 3958–3969. doi: 10.1002/hbm.20820

Han, X., Liu, X., Li, L., Xie, B., Fan, B., Qiu, Y., … Li, L. (2018). Neural Activation During Tonic Pain and Interaction Between Pain and Emotion in Bipolar Disorder: An fMRI Study. *Frontiers in Psychiatry*, *9*, 555. doi: 10.3389/fpsyt.2018.00555

Hansen, J. Y., Shafiei, G., Markello, R. D., Smart, K., Cox, S. M. L., Nørgaard, M., … Misic, B. (2022). Mapping neurotransmitter systems to the structural and functional organization of the human neocortex. *Nature Neuroscience*, *25*(11), 1569–1581. doi: 10.1038/s41593-022-01186-3

Hao, L., Yang, J., Wang, Y., Zhang, S., Xie, P., Luo, Q., … Qiu, J. (2015). Neural correlates of causal attribution in negative events of depressed patients: Evidence from an fMRI study. *Clinical Neurophysiology*, *126*(7), 1331–1337. doi: 10.1016/j.clinph.2014.10.146

Harrison, B. J., Yücel, M., Fornito, A., Wood, S. J., Seal, M. L., Clarke, K., & Pantelis, C. (2007). Characterizing anterior cingulate activation in chronic schizophrenia: A group and single-subject fMRI study. *Acta Psychiatrica Scandinavica*, *116*(4), 271–279. doi: 10.1111/j.1600-0447.2007.01002.x

Harvey, P.-O., Fossati, P., Pochon, J.-B., Levy, R., LeBastard, G., Lehéricy, S., … Dubois, B. (2005). Cognitive control and brain resources in major depression: An fMRI study using the n-back task. *NeuroImage*, *26*(3), 860–869. doi: 10.1016/j.neuroimage.2005.02.048

Harvey, P.-O., & Lepage, M. (2014). Neural correlates of recognition memory of social information in people with schizophrenia. *Journal of Psychiatry & Neuroscience: JPN*, *39*(2), 97–109. doi: 10.1503/jpn.130007

Hasenkamp, W., James, G. A., Boshoven, W., & Duncan, E. (2011). Altered engagement of attention and default networks during target detection in schizophrenia. *Schizophrenia Research*, *125*(2–3), 169–173. doi: 10.1016/j.schres.2010.08.041

Hassel, S., Almeida, J. R., Kerr, N., Nau, S., Ladouceur, C. D., Fissell, K., … Phillips, M. L. (2008). Elevated striatal and decreased dorsolateral prefrontal cortical activity in response to emotional stimuli in euthymic bipolar disorder: No associations with psychotropic medication load. *Bipolar Disorders*, *10*(8), 916–927. doi: 10.1111/j.1399-5618.2008.00641.x

Hawco, C., Buchy, L., Bodnar, M., Izadi, S., Dell’Elce, J., Messina, K., … Lepage, M. (2015). Source retrieval is not properly differentiated from object retrieval in early schizophrenia: An fMRI study using virtual reality. *NeuroImage: Clinical*, *7*, 336–346. doi: 10.1016/j.nicl.2014.08.006

Hazlett, E. A., Buchsbaum, M. S., Zhang, J., Newmark, R. E., Glanton, C. F., Zelmanova, Y., … Siever, L. J. (2008). Frontal–striatal–thalamic mediodorsal nucleus dysfunction in schizophrenia-spectrum patients during sensorimotor gating. *NeuroImage*, *42*(3), 1164–1177. doi: 10.1016/j.neuroimage.2008.05.039

He, Y., Steines, M., Sammer, G., Nagels, A., Kircher, T., & Straube, B. (2021). Modality-specific dysfunctional neural processing of social-abstract and non-social-concrete information in schizophrenia. *NeuroImage: Clinical*, *29*, 102568. doi: 10.1016/j.nicl.2021.102568

Heinze, S., Sartory, G., Müller, B. W., De Greiff, A., Forsting, M., & Jüptner, M. (2006). Neural activation during successful and unsuccessful verbal learning in schizophrenia. *Schizophrenia Research*, *83*(2–3), 121–130. doi: 10.1016/j.schres.2005.12.852

Heller, A. S., Johnstone, T., Shackman, A. J., Light, S. N., Peterson, M. J., Kolden, G. G., … Davidson, R. J. (2009). Reduced capacity to sustain positive emotion in major depression reflects diminished maintenance of fronto-striatal brain activation. *Proceedings of the National Academy of Sciences*, *106*(52), 22445–22450. doi: 10.1073/pnas.0910651106

Henseler, I., Falkai, P., & Gruber, O. (2009). A systematic fMRI investigation of the brain systems subserving different working memory components in schizophrenia. *European Journal of Neuroscience*, *30*(4), 693–702. doi: 10.1111/j.1460-9568.2009.06850.x

Herold, R., Varga, E., Hajnal, A., Hamvas, E., Berecz, H., Tóth, B., & Tényi, T. (2018). Altered Neural Activity during Irony Comprehension in Unaffected First-Degree Relatives of Schizophrenia Patients—An fMRI Study. *Frontiers in Psychology*, *8*, 2309. doi: 10.3389/fpsyg.2017.02309

Hofer, A., Weiss, E. M., Golaszewski, S. M., Siedentopf, C. M., Brinkhoff, C., Kremser, C., … Fleischhacker, W. W. (2003). Neural Correlates of Episodic Encoding and Recognition of Words in Unmedicated Patients During an Acute Episode of Schizophrenia: A Functional MRI Study. *American Journal of Psychiatry*, *160*(10), 1802–1808. doi: 10.1176/appi.ajp.160.10.1802

Holmes, A. J., MacDonald, A., Carter, C. S., Barch, D. M., Andrew Stenger, V., & Cohen, J. D. (2005). Prefrontal functioning during context processing in schizophrenia and major depression: An event-related fMRI study. *Schizophrenia Research*, *76*(2–3), 199–206. doi: 10.1016/j.schres.2005.01.021

Holt, D. J., Cassidy, B. S., Andrews-Hanna, J. R., Lee, S. M., Coombs, G., Goff, D. C., … Moran, J. M. (2011). An Anterior-to-Posterior Shift in Midline Cortical Activity in Schizophrenia During Self-Reflection. *Biological Psychiatry*, *69*(5), 415–423. doi: 10.1016/j.biopsych.2010.10.003

Holt, D. J., Coombs, G., Zeidan, M. A., Goff, D. C., & Milad, M. R. (2012). Failure of Neural Responses to Safety Cues in Schizophrenia. *Archives of General Psychiatry*, *69*(9), 893. doi: 10.1001/archgenpsychiatry.2011.2310

Honey, G. D., Sharma, T., Suckling, J., Giampietro, V., Soni, W., Williams, S. C. R., & Bullmore, E. T. (2003). The functional neuroanatomy of schizophrenic subsyndromes. *Psychological Medicine*, *33*(6), 1007–1018. doi: 10.1017/S0033291703007864

Hong, L. E., Tagamets, M., Avila, M., Wonodi, I., Holcomb, H., & Thaker, G. K. (2005). Specific motion processing pathway deficit during eye tracking in schizophrenia: A performance-matched functional magnetic resonance imaging study. *Biological Psychiatry*, *57*(7), 726–732. doi: 10.1016/j.biopsych.2004.12.015

Horan, W. P., Jimenez, A. M., Lee, J., Wynn, J. K., Eisenberger, N. I., & Green, M. F. (2016). Pain empathy in schizophrenia: An fMRI study. *Social Cognitive and Affective Neuroscience*, *11*(5), 783–792. doi: 10.1093/scan/nsw002

Huang, A. S., Rogers, B. P., Anticevic, A., Blackford, J. U., Heckers, S., & Woodward, N. D. (2019). Brain function during stages of working memory in schizophrenia and psychotic bipolar disorder. *Neuropsychopharmacology*, *44*(12), 2136–2142. doi: 10.1038/s41386-019-0434-4

Huang, C.-M., Fan, Y.-T., Lee, S.-H., Liu, H.-L., Chen, Y.-L., Lin, C., & Lee, T. M. C. (2019). Cognitive reserve-mediated neural modulation of emotional control and regulation in people with late-life depression. *Social Cognitive and Affective Neuroscience*, *14*(8), 849–860. doi: 10.1093/scan/nsz054

Huang, J., Yang, X., Lan, Y., Zhu, C., Liu, X., Wang, Y., … Chan, R. C. K. (2016). Neural substrates of the impaired effort expenditure decision making in schizophrenia. *Neuropsychology*, *30*(6), 685–696. doi: 10.1037/neu0000284

Hugdahl, K., Rund, B. R., Lund, A., Asbjørnsen, A., Egeland, J., Ersland, L., … Thomsen, T. (2004). Brain Activation Measured With fMRI During a Mental Arithmetic Task in Schizophrenia and Major Depression. *American Journal of Psychiatry*, *161*(2), 286–293. doi: 10.1176/appi.ajp.161.2.286

Hugdahl, K., Specht, K., Biringer, E., Weis, S., Elliott, R., Hammar, Å., … Lund, A. (2007). Increased Parietal and Frontal Activation after Remission from Recurrent Major Depression: A Repeated fMRI Study. *Cognitive Therapy and Research*, *31*(2), 147–160. doi: 10.1007/s10608-006-9116-8

Hughes, M. E., Fulham, W. R., Johnston, P. J., & Michie, P. T. (2012). Stop-signal response inhibition in schizophrenia: Behavioural, event-related potential and functional neuroimaging data. *Biological Psychology*, *89*(1), 220–231. doi: 10.1016/j.biopsycho.2011.10.013

Hulvershorn, L. A., Karne, H., Gunn, A. D., Hartwick, S. L., Wang, Y., Hummer, T. A., & Anand, A. (2012). Neural Activation During Facial Emotion Processing in Unmedicated Bipolar Depression, Euthymia, and Mania. *Biological Psychiatry*, *71*(7), 603–610. doi: 10.1016/j.biopsych.2011.10.038

Hutcheson, N. L., Reid, M. A., White, D. M., Kraguljac, N. V., Avsar, K. B., Bolding, M. S., … Lahti, A. C. (2012). Multimodal analysis of the hippocampus in schizophrenia using proton magnetic resonance spectroscopy and functional magnetic resonance imaging. *Schizophrenia Research*, *140*(1–3), 136–142. doi: 10.1016/j.schres.2012.06.039

Insel, C., Glenn, C. R., Nock, M. K., & Somerville, L. H. (2019). Aberrant striatal tracking of reward magnitude in youth with current or past-year depression. *Journal of Abnormal Psychology*, *128*(1), 44–56. doi: 10.1037/abn0000389

Iwashiro, N., Takano, Y., Natsubori, T., Aoki, Y., Yahata, N., Gonoi, W., … Yamasue, H. (2019). Aberrant attentive and inattentive brain activity to auditory negative words, and its relation to persecutory delusion in patients with schizophrenia. *Neuropsychiatric Disease and Treatment*, *Volume 15*, 491–502. doi: 10.2147/NDT.S194353

Jamadar, S., Michie, P., & Karayanidis, F. (2010). Compensatory mechanisms underlie intact task-switching performance in schizophrenia. *Neuropsychologia*, *48*(5), 1305–1323. doi: 10.1016/j.neuropsychologia.2009.12.034

Jamadar, Sharna, O’Neil, K. M., Pearlson, G. D., Ansari, M., Gill, A., Jagannathan, K., & Assaf, M. (2013). Impairment in Semantic Retrieval is Associated with Symptoms in Schizophrenia but not Bipolar Disorder. *Biological Psychiatry*, *73*(6), 555–564. doi: 10.1016/j.biopsych.2012.07.027

Jia, W., Zhu, H., Ni, Y., Su, J., Xu, R., Jia, H., & Wan, X. (2020). Disruptions of frontoparietal control network and default mode network linking the metacognitive deficits with clinical symptoms in schizophrenia. *Human Brain Mapping*, *41*(6), 1445–1458. doi: 10.1002/hbm.24887

Jiang, S., Yan, H., Chen, Q., Tian, L., Lu, T., Tan, H.-Y., … Zhang, D. (2015). Cerebral Inefficient Activation in Schizophrenia Patients and Their Unaffected Parents during the N-Back Working Memory Task: A Family fMRI Study. *PLOS ONE*, *10*(8), e0135468. doi: 10.1371/journal.pone.0135468

Jimenez, A. M., Lee, J., Reavis, E. A., Wynn, J. K., & Green, M. F. (2018). Aberrant patterns of neural activity when perceiving emotion from biological motion in schizophrenia. *NeuroImage: Clinical*, *20*, 380–387. doi: 10.1016/j.nicl.2018.08.014

Jimenez, A. M., Lee, J., Wynn, J. K., & Green, M. F. (2018). The neural correlates of self-referential memory encoding and retrieval in schizophrenia. *Neuropsychologia*, *109*, 19–27. doi: 10.1016/j.neuropsychologia.2017.12.004

Jiménez, J. A., Mancini-Marïe, A., Lakis, N., Rinaldi, M., & Mendrek, A. (2010). Disturbed sexual dimorphism of brain activation during mental rotation in schizophrenia. *Schizophrenia Research*, *122*(1–3), 53–62. doi: 10.1016/j.schres.2010.03.011

Jogia, J., Dima, D., Kumari, V., & Frangou, S. (2012). Frontopolar cortical inefficiency may underpin reward and working memory dysfunction in bipolar disorder. *The World Journal of Biological Psychiatry*, *13*(8), 605–615. doi: 10.3109/15622975.2011.585662

Jogia, J., Haldane, M., Cobb, A., Kumari, V., & Frangou, S. (2008). Pilot investigation of the changes in cortical activation during facial affect recognition with lamotrigine monotherapy in bipolar disorder. *British Journal of Psychiatry*, *192*(3), 197–201. doi: 10.1192/bjp.bp.107.037960

John, J. P., Halahalli, H. N., Vasudev, M. K., Jayakumar, P. N., & Jain, S. (2011). Regional brain activation/deactivation during word generation in schizophrenia: fMRI study. *British Journal of Psychiatry*, *198*(3), 213–222. doi: 10.1192/bjp.bp.110.083501

Johnson, M. R., Morris, N. A., Astur, R. S., Calhoun, V. D., Mathalon, D. H., Kiehl, K. A., & Pearlson, G. D. (2006). A Functional Magnetic Resonance Imaging Study of Working Memory Abnormalities in Schizophrenia. *Biological Psychiatry*, *60*(1), 11–21. doi: 10.1016/j.biopsych.2005.11.012

Johnson, S. L., Mehta, H., Ketter, T. A., Gotlib, I. H., & Knutson, B. (2019). Neural responses to monetary incentives in bipolar disorder. *NeuroImage: Clinical*, *24*, 102018. doi: 10.1016/j.nicl.2019.102018

Johnston, B. A., Tolomeo, S., Gradin, V., Christmas, D., Matthews, K., & Douglas Steele, J. (2015). Failure of hippocampal deactivation during loss events in treatment-resistant depression. *Brain*, *138*(9), 2766–2776. doi: 10.1093/brain/awv177

Johnstone, T., Van Reekum, C. M., Urry, H. L., Kalin, N. H., & Davidson, R. J. (2007). Failure to Regulate: Counterproductive Recruitment of Top-Down Prefrontal-Subcortical Circuitry in Major Depression. *The Journal of Neuroscience*, *27*(33), 8877–8884. doi: 10.1523/JNEUROSCI.2063-07.2007

Joshi, S. H., Vizueta, N., Foland-Ross, L., Townsend, J. D., Bookheimer, S. Y., Thompson, P. M., … Altshuler, L. L. (2016). Relationships Between Altered Functional Magnetic Resonance Imaging Activation and Cortical Thickness in Patients With Euthymic Bipolar I Disorder. *Biological Psychiatry: Cognitive Neuroscience and Neuroimaging*, *1*(6), 507–517. doi: 10.1016/j.bpsc.2016.06.006

Joyal, C. C., Putkonen, A., Mancini-Marïe, A., Hodgins, S., Kononen, M., Boulay, L., … Aronen, H. J. (2007). Violent persons with schizophrenia and comorbid disorders: A functional magnetic resonance imaging study. *Schizophrenia Research*, *91*(1–3), 97–102. doi: 10.1016/j.schres.2006.12.014

Kaladjian, A., Jeanningros, R., Azorin, J.-M., Grimault, S., Anton, J.-L., & Mazzola-Pomietto, P. (2007). Blunted activation in right ventrolateral prefrontal cortex during motor response inhibition in schizophrenia. *Schizophrenia Research*, *97*(1–3), 184–193. doi: 10.1016/j.schres.2007.07.033

Kaladjian, A., Jeanningros, R., Azorin, J.-M., Nazarian, B., Roth, M., Anton, J.-L., & Mazzola-Pomietto, P. (2009). Remission from mania is associated with a decrease in amygdala activation during motor response inhibition. *Bipolar Disorders*, *11*(5), 530–538. doi: 10.1111/j.1399-5618.2009.00722.x

Kaladjian, A., Jeanningros, R., Azorin, J.-M., Nazarian, B., Roth, M., & Mazzola-Pomietto, P. (2009). Reduced brain activation in euthymic bipolar patients during response inhibition: An event-related fMRI study. *Psychiatry Research: Neuroimaging*, *173*(1), 45–51. doi: 10.1016/j.pscychresns.2008.08.003

Kang, J. I., Kim, J.-J., Seok, J.-H., Chun, J. W., Lee, S.-K., & Park, H.-J. (2009). Abnormal brain response during the auditory emotional processing in schizophrenic patients with chronic auditory hallucinations. *Schizophrenia Research*, *107*(1), 83–91. doi: 10.1016/j.schres.2008.08.019

Karpouzian, T. M., Schroeder, M. P., Abram, S. V., Wanar, H., Alden, E. C., Eack, S. M., … Smith, M. J. (2017). Neural correlates of preserved facial affect perception in high functioning schizophrenia. *Psychiatry Research: Neuroimaging*, *266*, 83–85. doi: 10.1016/j.pscychresns.2017.06.002

Kassel, M. T., Rao, J. A., Walker, S. J., Briceño, E. M., Gabriel, L. B., Weldon, A. L., … Langenecker, S. A. (2016). Decreased Fronto-Limbic Activation and Disrupted Semantic-Cued List Learning in Major Depressive Disorder. *Journal of the International Neuropsychological Society*, *22*(4), 412–425. doi: 10.1017/S1355617716000023

Katayama, N., Nakagawa, A., Umeda, S., Terasawa, Y., Kurata, C., Tabuchi, H., … Mimura, M. (2019). Frontopolar cortex activation associated with pessimistic future-thinking in adults with major depressive disorder. *NeuroImage: Clinical*, *23*, 101877. doi: 10.1016/j.nicl.2019.101877

Keedwell, P. A., Andrew, C., Williams, S. C. R., Brammer, M. J., & Phillips, M. L. (2005). A Double Dissociation of Ventromedial Prefrontal Cortical Responses to Sad and Happy Stimuli in Depressed and Healthy Individuals. *Biological Psychiatry*, *58*(6), 495–503. doi: 10.1016/j.biopsych.2005.04.035

Keedy, S. K., Reilly, J. L., Bishop, J. R., Weiden, P. J., & Sweeney, J. A. (2015). Impact of Antipsychotic Treatment on Attention and Motor Learning Systems in First-Episode Schizophrenia. *Schizophrenia Bulletin*, *41*(2), 355–365. doi: 10.1093/schbul/sbu071

Keedy, S. K., Rosen, C., Khine, T., Rajarethinam, R., Janicak, P. G., & Sweeney, J. A. (2009). An fMRI study of visual attention and sensorimotor function before and after antipsychotic treatment in first-episode schizophrenia. *Psychiatry Research: Neuroimaging*, *172*(1), 16–23. doi: 10.1016/j.pscychresns.2008.06.003

Keener, M. T., Fournier, J. C., Mullin, B. C., Kronhaus, D., Perlman, S. B., LaBarbara, E., … Phillips, M. L. (2012). Dissociable patterns of medial prefrontal and amygdala activity to face identity *versus* emotion in bipolar disorder. *Psychological Medicine*, *42*(9), 1913–1924. doi: 10.1017/S0033291711002935

Kerns, J. G., Cohen, J. D., MacDonald, A. W., Johnson, M. K., Stenger, V. A., Aizenstein, H., & Carter, C. S. (2005). Decreased Conflict- and Error-Related Activity in the Anterior Cingulate Cortex in Subjects With Schizophrenia. *American Journal of Psychiatry*, *162*(10), 1833–1839. doi: 10.1176/appi.ajp.162.10.1833

Killgore, W. D. S., Gruber, S. A., & Yurgelun-Todd, D. A. (2008). Abnormal corticostriatal activity during fear perception in bipolar disorder. *NeuroReport*, *19*(15), 1523–1527. doi: 10.1097/WNR.0b013e328310af58

Kim, E., Jung, Y.-C., Ku, J., Kim, J.-J., Lee, H., Kim, S. Y., … Cho, H.-S. (2009). Reduced activation in the mirror neuron system during a virtual social cognition task in euthymic bipolar disorder. *Progress in Neuro-Psychopharmacology and Biological Psychiatry*, *33*(8), 1409–1416. doi: 10.1016/j.pnpbp.2009.07.019

Kim, G.-W., Yang, J.-C., & Jeong, G.-W. (2015). Emotional effect on cognitive control in implicit memory tasks in patients with schizophrenia. *NeuroReport*, *26*(11), 647–655. doi: 10.1097/WNR.0000000000000405

Kim, J., Matthews, N. L., & Park, S. (2010). An Event-Related fMRI Study of Phonological Verbal Working Memory in Schizophrenia. *PLoS ONE*, *5*(8), e12068. doi: 10.1371/journal.pone.0012068

King, J. B., Anderson, J. S., Yurgelun-Todd, D. A., Subramaniam, P., Ehrler, M. R., & Lopez-Larson, M. P. (2018). Decreased anterior cingulate activation in a motor task in youths with bipolar disorder. *Journal of Child Psychology and Psychiatry*, *59*(8), 900–907. doi: 10.1111/jcpp.12875

Kircher, T. T. J., Bulimore, E. T., Brammer, M. J., Williams, S. C. R., Broome, M. R., Murray, R. M., & McGuire, P. K. (2001). Differential activation of temporal cortex during sentence completion in schizophrenic patients with and without formal thought disorder. *Schizophrenia Research*, *50*(1–2), 27–40. doi: 10.1016/S0920-9964(00)00042-6

Kircher, T. T. J., Leube, D. T., Erb, M., Grodd, W., & Rapp, A. M. (2007). Neural correlates of metaphor processing in schizophrenia. *NeuroImage*, *34*(1), 281–289. doi: 10.1016/j.neuroimage.2006.08.044

Kircher, T., Whitney, C., Krings, T., Huber, W., & Weis, S. (2008). Hippocampal dysfunction during free word association in male patients with schizophrenia. *Schizophrenia Research*, *101*(1–3), 242–255. doi: 10.1016/j.schres.2008.02.003

Kirschner, M., Hager, O. M., Bischof, M., Hartmann-Riemer, M. N., Kluge, A., Seifritz, E., … Kaiser, S. (2016). Deficits in context-dependent adaptive coding of reward in schizophrenia. *Npj Schizophrenia*, *2*(1), 16020. doi: 10.1038/npjschz.2016.20

Knolle, F., Ermakova, A. O., Justicia, A., Fletcher, P. C., Bunzeck, N., Düzel, E., & Murray, G. K. (2018). Brain responses to different types of salience in antipsychotic naïve first episode psychosis: An fMRI study. *Translational Psychiatry*, *8*(1), 196. doi: 10.1038/s41398-018-0250-3

Koch, K., Wagner, G., Nenadic, I., Schachtzabel, C., Schultz, C., Roebel, M., … Schlösser, R. G. M. (2008). Fronto-striatal hypoactivation during correct information retrieval in patients with schizophrenia: An fMRI study. *Neuroscience*, *153*(1), 54–62. doi: 10.1016/j.neuroscience.2008.01.063

Koch, Kathrin, Wagner, G., Schachtzabel, C., Schultz, C. C., Güllmar, D., Reichenbach, J. R., … Schlösser, R. G. M. (2011). Neural activation and radial diffusivity in schizophrenia: Combined fMRI and diffusion tensor imaging study. *British Journal of Psychiatry*, *198*(3), 223–229. doi: 10.1192/bjp.bp.110.081836

Koeda, M., Takahashi, H., Yahata, N., Matsuura, M., Asai, K., Okubo, Y., & Tanaka, H. (2006). Language Processing and Human Voice Perception in Schizophrenia: A Functional Magnetic Resonance Imaging Study. *Biological Psychiatry*, *59*(10), 948–957. doi: 10.1016/j.biopsych.2006.01.013

Kohler, C. G., Loughead, J., Ruparel, K., Indersmitten, T., Barrett, F. S., Gur, R. E., & Gur, R. C. (2008). Brain activation during eye gaze discrimination in stable schizophrenia. *Schizophrenia Research*, *99*(1–3), 286–293. doi: 10.1016/j.schres.2007.09.038

Köhler, S., Wagner, G., & Bär, K. (2019). Activation of brainstem and midbrain nuclei during cognitive control in medicated patients with schizophrenia. *Human Brain Mapping*, *40*(1), 202–213. doi: 10.1002/hbm.24365

Kosaka, H., Omori, M., Murata, T., Iidaka, T., Yamada, H., Okada, T., … Wada, Y. (2002). Differential amygdala response during facial recognition in patients with schizophrenia: An fMRI study. *Schizophrenia Research*, *57*(1), 87–95. doi: 10.1016/S0920-9964(01)00324-3

Krabbendam, L., O’Daly, O., Morley, L. A., van Os, J., Murray, R. M., & Shergill, S. S. (2009). Using the Stroop task to investigate the neural correlates of symptom change in schizophrenia. *British Journal of Psychiatry*, *194*(4), 373–374. doi: 10.1192/bjp.bp.108.055459

Kronbichler, L., Stelzig-Schöler, R., Pearce, B.-G., Tschernegg, M., Said-Yürekli, S., Crone, J. S., … Kronbichler, M. (2019). Reduced spontaneous perspective taking in schizophrenia. *Psychiatry Research: Neuroimaging*, *292*, 5–12. doi: 10.1016/j.pscychresns.2019.08.007

Kronhaus, D. M., Lawrence, N. S., Williams, A. M., Frangou, S., Brammer, M. J., Williams, S. C., … Phillips, M. L. (2006). Stroop performance in bipolar disorder: Further evidence for abnormalities in the ventral prefrontal cortex. *Bipolar Disorders*, *8*(1), 28–39. doi: 10.1111/j.1399-5618.2006.00282.x

Krug, A., Cabanis, M., Pyka, M., Pauly, K., Kellermann, T., Walter, H., … Kircher, T. (2014). Attenuated prefrontal activation during decision-making under uncertainty in schizophrenia: A multi-center fMRI study. *Schizophrenia Research*, *152*(1), 176–183. doi: 10.1016/j.schres.2013.11.007

Kubicki, M. (2003). An fMRI study of semantic processing in men with schizophrenia. *NeuroImage*, *20*(4), 1923–1933. doi: 10.1016/S1053-8119(03)00383-5

Kumari, V., Antonova, E., Geyer, M. A., ffytche, D., Williams, S. C. R., & Sharma, T. (2007). A fMRI investigation of startle gating deficits in schizophrenia patients treated with typical or atypical antipsychotics. *The International Journal of Neuropsychopharmacology*, *10*(04), 463. doi: 10.1017/S1461145706007139

Kumari, V., Fannon, D., ffytche, D. H., Raveendran, V., Antonova, E., Premkumar, P., … Kuipers, E. (2010). Functional MRI of Verbal Self-monitoring in Schizophrenia: Performance and Illness-Specific Effects. *Schizophrenia Bulletin*, *36*(4), 740–755. doi: 10.1093/schbul/sbn148

Kumari, V., Mitterschiffthaler, M. T., Teasdale, J. D., Malhi, G. S., Brown, R. G., Giampietro, V., … Sharma, T. (2003). Neural abnormalities during cognitive generation of affect in Treatment-Resistant depression. *Biological Psychiatry*, *54*(8), 777–791. doi: 10.1016/S0006-3223(02)01785-7

Lagopoulos, J., Ivanovski, B., & Malhi, G. S. (2007). An event-related functional MRI study of working memory in euthymic bipolar disorder. *Journal of Psychiatry & Neuroscience: JPN*, *32*(3), 174–184.

Lagopoulos, J., & Malhi, G. S. (2007). A functional magnetic resonance imaging study of emotional Stroop in euthymic bipolar disorder. *NeuroReport*, *18*(15), 1583–1587. doi: 10.1097/WNR.0b013e3282efa07a

Lakis, N., Jiménez, J. A., Mancini-Marïe, A., Stip, E., Lavoie, M. E., & Mendrek, A. (2011). Neural correlates of emotional recognition memory in schizophrenia: Effects of valence and arousal. *Psychiatry Research: Neuroimaging*, *194*(3), 245–256. doi: 10.1016/j.pscychresns.2011.05.010

Landin-Romero, R., McKenna, P. J., Salgado-Pineda, P., Sarró, S., Aguirre, C., Sarri, C., … Pomarol-Clotet, E. (2015). Failure of deactivation in the default mode network: A trait marker for schizophrenia? *Psychological Medicine*, *45*(6), 1315–1325. doi: 10.1017/S0033291714002426

Langenecker, S. A., Jenkins, L. M., Stange, J. P., Chang, Y.-S., DelDonno, S. R., Bessette, K. L., … Jacobs, R. H. (2018). Cognitive control neuroimaging measures differentiate between those with and without future recurrence of depression. *NeuroImage: Clinical*, *20*, 1001–1009. doi: 10.1016/j.nicl.2018.10.004

Langenecker, S. A., Kennedy, S. E., Guidotti, L. M., Briceno, E. M., Own, L. S., Hooven, T., … Zubieta, J.-K. (2007). Frontal and Limbic Activation During Inhibitory Control Predicts Treatment Response in Major Depressive Disorder. *Biological Psychiatry*, *62*(11), 1272–1280. doi: 10.1016/j.biopsych.2007.02.019

Laurens, K. R. (2003). Rostral anterior cingulate cortex dysfunction during error processing in schizophrenia. *Brain*, *126*(3), 610–622. doi: 10.1093/brain/awg056

Laurens, Kristin R., Kiehl, K. A., Ngan, E. T. C., & Liddle, P. F. (2005). Attention orienting dysfunction during salient novel stimulus processing in schizophrenia. *Schizophrenia Research*, *75*(2–3), 159–171. doi: 10.1016/j.schres.2004.12.010

Lee, H., Ku, J., Kim, J., Jang, D.-P., Yoon, K. J., Kim, S. I., & Kim, J.-J. (2014). Aberrant neural responses to social rejection in patients with schizophrenia. *Social Neuroscience*, *9*(4), 412–423. doi: 10.1080/17470919.2014.907202

Lee, J., Folley, B. S., Gore, J., & Park, S. (2008). Origins of Spatial Working Memory Deficits in Schizophrenia: An Event-Related fMRI and Near-Infrared Spectroscopy Study. *PLoS ONE*, *3*(3), e1760. doi: 10.1371/journal.pone.0001760

Lee, J., Quintana, J., Nori, P., & Green, M. F. (2011). Theory of mind in schizophrenia: Exploring neural mechanisms of belief attribution. *Social Neuroscience*, *6*(5–6), 569–581. doi: 10.1080/17470919.2011.620774

Lee, J., Reavis, E. A., Engel, S. A., Altshuler, L. L., Cohen, M. S., Glahn, D. C., … Green, M. F. (2019). fMRI evidence of aberrant neural adaptation for objects in schizophrenia and bipolar disorder. *Human Brain Mapping*, *40*(5), 1608–1617. doi: 10.1002/hbm.24472

Lee, J. S., Chun, J. W., Lee, S.-H., Kim, E., Lee, S.-K., & Kim, J.-J. (2015). Altered Neural Basis of the Reality Processing and Its Relation to Cognitive Insight in Schizophrenia. *PLOS ONE*, *10*(3), e0120478. doi: 10.1371/journal.pone.0120478

Lee, J. S., Chun, J. W., Yoon, S. Y., Park, H.-J., & Kim, J.-J. (2014). Involvement of the mirror neuron system in blunted affect in schizophrenia. *Schizophrenia Research*, *152*(1), 268–274. doi: 10.1016/j.schres.2013.10.043

Lee, K.-H., Brown, W. H., Egleston, P. N., Green, R. D. J., Farrow, T. F. D., Hunter, M. D., … Woodruff, P. W. R. (2006). A Functional Magnetic Resonance Imaging Study of Social Cognition in Schizophrenia During an Acute Episode and After Recovery. *American Journal of Psychiatry*, *163*(11), 1926–1933. doi: 10.1176/ajp.2006.163.11.1926

Lee, K.-H., Pluck, G., Lekka, N., Horton, A., Wilkinson, I. D., & Woodruff, P. W. R. (2015). Self-harm in schizophrenia is associated with dorsolateral prefrontal and posterior cingulate activity. *Progress in Neuro-Psychopharmacology and Biological Psychiatry*, *61*, 18–23. doi: 10.1016/j.pnpbp.2015.03.005

Lee, S. J., Kang, D. H., Kim, C.-W., Gu, B. M., Park, J.-Y., Choi, C.-H., … Kwon, J. S. (2010). Multi-level comparison of empathy in schizophrenia: An fMRI study of a cartoon task. *Psychiatry Research: Neuroimaging*, *181*(2), 121–129. doi: 10.1016/j.pscychresns.2009.08.003

Lee, S.-K., Chun, J. W., Lee, J. S., Park, H.-J., Jung, Y.-C., Seok, J.-H., & Kim, J.-J. (2014). Abnormal Neural Processing during Emotional Salience Attribution of Affective Asymmetry in Patients with Schizophrenia. *PLoS ONE*, *9*(3), e90792. doi: 10.1371/journal.pone.0090792

Lee, T.-W., Liu, H.-L., Wai, Y.-Y., Ko, H.-J., & Lee, S.-H. (2013). Abnormal neural activity in partially remitted late-onset depression: An fMRI study of one-back working memory task. *Psychiatry Research: Neuroimaging*, *213*(2), 133–141. doi: 10.1016/j.pscychresns.2012.04.010

Lefebvre, S., Very, E., Jardri, R., Horn, M., Yrondi, A., Delmaire, C., … Pins, D. (2021). The neural correlates of the visual consciousness in schizophrenia: An fMRI study. *European Archives of Psychiatry and Clinical Neuroscience*, *271*(4), 661–675. doi: 10.1007/s00406-020-01167-2

Leitman, D. I., Wolf, D. H., Laukka, P., Ragland, J. D., Valdez, J. N., Turetsky, B. I., … Gur, R. C. (2011). Not Pitch Perfect: Sensory Contributions to Affective Communication Impairment in Schizophrenia. *Biological Psychiatry*, *70*(7), 611–618. doi: 10.1016/j.biopsych.2011.05.032

Lemke, H., Probst, S., Warneke, A., Waltemate, L., Winter, A., Thiel, K., … Dannlowski, U. (2022). The Course of Disease in Major Depressive Disorder Is Associated With Altered Activity of the Limbic System During Negative Emotion Processing. *Biological Psychiatry: Cognitive Neuroscience and Neuroimaging*, *7*(3), 323–332. doi: 10.1016/j.bpsc.2021.05.008

Lemmers-Jansen, I. L. J., Fett, A.-K. J., Hanssen, E., Veltman, D. J., & Krabbendam, L. (2019). Learning to trust: Social feedback normalizes trust behavior in first-episode psychosis and clinical high risk. *Psychological Medicine*, *49*(5), 780–790. doi: 10.1017/S003329171800140X

Lencer, R., Nagel, M., Sprenger, A., Heide, W., & Binkofski, F. (2005). Reduced neuronal activity in the V5 complex underlies smooth-pursuit deficit in schizophrenia: Evidence from an fMRI study. *NeuroImage*, *24*(4), 1256–1259. doi: 10.1016/j.neuroimage.2004.11.013

Lennox, B. R., Jacob, R., Calder, A. J., Lupson, V., & Bullmore, E. T. (2004). Behavioural and neurocognitive responses to sad facial affect are attenuated in patients with mania. *Psychological Medicine*, *34*(5), 795–802. doi: 10.1017/S0033291704002557

Lepage, M., Sergerie, K., Benoit, A., Czechowska, Y., Dickie, E., & Armony, J. L. (2011). Emotional face processing and flat affect in schizophrenia: Functional and structural neural correlates. *Psychological Medicine*, *41*(9), 1833–1844. doi: 10.1017/S0033291711000031

Lepage, Martin, Montoya, A., Pelletier, M., Achim, A. M., Menear, M., & Lal, S. (2006). Associative Memory Encoding and Recognition in Schizophrenia: An Event-Related fMRI Study. *Biological Psychiatry*, *60*(11), 1215–1223. doi: 10.1016/j.biopsych.2006.03.043

Leube, D. T., Knoblich, G., Erb, M., Schlotterbeck, P., & Kircher, T. T. J. (2010). The neural basis of disturbed efference copy mechanism in patients with schizophrenia. *Cognitive Neuroscience*, *1*(2), 111–117. doi: 10.1080/17588921003646156

Leube, D. T., Rapp, A., Erb, M., Grodd, W., Buchkremer, G., Bartels, M., & Kircher, T. T. J. (2003). Hippocampal dysfunction during episodic memory encoding in patients with schizophrenia—An fMRI study. *Schizophrenia Research*, *64*(1), 83–85. doi: 10.1016/S0920-9964(02)00503-0

Li, H., Chan, R. C. K., Gong, Q., Liu, Y., Liu, S., Shum, D., & Ma, Z. (2012). Facial emotion processing in patients with schizophrenia and their non-psychotic siblings: A functional magnetic resonance imaging study. *Schizophrenia Research*, *134*(2–3), 143–150. doi: 10.1016/j.schres.2011.10.019

Li, Linling, Ji, E., Tang, F., Qiu, Y., Han, X., Zhang, S., … Yang, H. (2019). Abnormal brain activation during emotion processing of euthymic bipolar patients taking different mood stabilizers. *Brain Imaging and Behavior*, *13*(4), 905–913. doi: 10.1007/s11682-018-9915-z

Li, Liyuan, Li, R., Shen, F., Wang, X., Zou, T., Deng, C., … Chen, H. (2022). Negative bias effects during audiovisual emotional processing in major depression disorder. *Human Brain Mapping*, *43*(4), 1449–1462. doi: 10.1002/hbm.25735

Li, X., Yi, Z., Lv, Q., Chu, M., Hu, H., Wang, J., … Chan, R. C. K. (2019). Clinical utility of the dual n-back task in schizophrenia: A functional imaging approach. *Psychiatry Research: Neuroimaging*, *284*, 37–44. doi: 10.1016/j.pscychresns.2019.01.002

Li, Z., Huang, J., Hung, K. S. Y., Deng, Y., Wang, Y., Wang, Y., … Chan, R. C. K. (2021). Cerebellar hypoactivation is associated with impaired sensory integration in schizophrenia. *Journal of Abnormal Psychology*, *130*(1), 102–111. doi: 10.1037/abn0000636

Li, Z., Yan, C., Lv, Q., Yi, Z., Zhang, J., Wang, J., … Chan, R. C. K. (2018). Striatal dysfunction in patients with schizophrenia and their unaffected first-degree relatives. *Schizophrenia Research*, *195*, 215–221. doi: 10.1016/j.schres.2017.08.043

Liddle, P. F., Laurens, K. R., Kiehl, K. A., & Ngan, E. T. C. (2006). Abnormal function of the brain system supporting motivated attention in medicated patients with schizophrenia: An fMRI study. *Psychological Medicine*, *36*(8), 1097–1108. doi: 10.1017/S0033291706007677

Lindner, C., Dannlowski, U., Walhöfer, K., Rödiger, M., Maisch, B., Bauer, J., … Suslow, T. (2014). Social Alienation in Schizophrenia Patients: Association with Insula Responsiveness to Facial Expressions of Disgust. *PLoS ONE*, *9*(1), e85014. doi: 10.1371/journal.pone.0085014

Linnman, C., Coombs, G., Goff, D. C., & Holt, D. J. (2013). Lack of insula reactivity to aversive stimuli in schizophrenia. *Schizophrenia Research*, *143*(1), 150–157. doi: 10.1016/j.schres.2012.10.038

Lisiecka, D., Carballedo, A., Fagan, A., Ferguson, Y., Meaney, J., & Frodl, T. (2013). Recruitment of the left hemispheric emotional attention neural network in risk for and protection from depression. *Journal of Psychiatry & Neuroscience*, *38*(2), 117–128. doi: 10.1503/jpn.110188

Liu, Y., Bi, T., Kuang, Q., Zhang, B., Wu, H., Li, H., … Zheng, Y. (2021). Cortical Pathways or Mechanism in the Face Inversion Effect in Patients with First-Episode Schizophrenia. *Neuropsychiatric Disease and Treatment*, *Volume 17*, 1893–1906. doi: 10.2147/NDT.S302584

Loeb, F. F., Zhou, X., Craddock, K. E. S., Shora, L., Broadnax, D. D., Gochman, P., … Liu, S. (2018). Reduced Functional Brain Activation and Connectivity During a Working Memory Task in Childhood-Onset Schizophrenia. *Journal of the American Academy of Child & Adolescent Psychiatry*, *57*(3), 166–174. doi: 10.1016/j.jaac.2017.12.009

Loeffler, L. A. K., Radke, S., Habel, U., Ciric, R., Satterthwaite, T. D., Schneider, F., & Derntl, B. (2018). The regulation of positive and negative emotions through instructed causal attributions in lifetime depression – A functional magnetic resonance imaging study. *NeuroImage: Clinical*, *20*, 1233–1245. doi: 10.1016/j.nicl.2018.10.025

Lois, G., Schneider, E. E., Kaurin, A., & Wessa, M. (2020). Altered neural responses to social fairness in bipolar disorder. *NeuroImage: Clinical*, *28*, 102487. doi: 10.1016/j.nicl.2020.102487

Lopez-Garcia, P., Cristobal-Huerta, A., Young Espinoza, L., Molero, P., Ortuño Sanchez-Pedreño, F., & Hernández-Tamames, J. A. (2016). The influence of the COMT genotype in the underlying functional brain activity of context processing in schizophrenia and in relatives. *Progress in Neuro-Psychopharmacology and Biological Psychiatry*, *71*, 176–182. doi: 10.1016/j.pnpbp.2016.07.005

López-Solà, M., Pujol, J., Hernández-Ribas, R., Harrison, B. J., Contreras-Rodríguez, O., Soriano-Mas, C., … Cardoner, N. (2010). Effects of Duloxetine Treatment on Brain Response to Painful Stimulation in Major Depressive Disorder. *Neuropsychopharmacology*, *35*(11), 2305–2317. doi: 10.1038/npp.2010.108

Lošák, J., Hüttlová, J., Lipová, P., Mareček, R., Bareš, M., Filip, P., … Kašpárek, T. (2016). Predictive Motor Timing and the Cerebellar Vermis in Schizophrenia: An fMRI Study. *Schizophrenia Bulletin*, *42*(6), 1517–1527. doi: 10.1093/schbul/sbw065

Luck, D., Danion, J.-M., Marrer, C., Pham, B.-T., Gounot, D., & Foucher, J. (2009). Abnormal medial temporal activity for bound information during working memory maintenance in patients with schizophrenia. *Hippocampus*, NA-NA. doi: 10.1002/hipo.20689

Luck, D., Joober, R., Malla, A., & Lepage, M. (2016). Altered emotional modulation of associative memory in first episode schizophrenia: An fMRI study. *Schizophrenia Research: Cognition*, *3*, 26–32. doi: 10.1016/j.scog.2015.11.004

Lundin, N. B., Kim, D.-J., Tullar, R. L., Moussa-Tooks, A. B., Kent, J. S., Newman, S. D., … Hetrick, W. P. (2021). Cerebellar Activation Deficits in Schizophrenia During an Eyeblink Conditioning Task. *Schizophrenia Bulletin Open*, *2*(1), sgab040. doi: 10.1093/schizbullopen/sgab040

Madre, M., Radua, J., Landin-Romero, R., Alonso-Lana, S., Salvador, R., Panicali, F., … Amann, B. L. (2014). Trait or state? A longitudinal neuropsychological evaluation and fMRI study in schizoaffective disorder. *Schizophrenia Research*, *159*(2–3), 458–464. doi: 10.1016/j.schres.2014.08.017

Maïza, O. (2010). Impact of cognitive performance on the reproducibility of fMRI activation in schizophrenia. *Journal of Psychiatry & Neuroscience*, *35*(6), 378–389. doi: 10.1503/jpn.090103

Makowski, C. S., Lepage, M., & Harvey, P.-O. (2016). Functional neural correlates of social approval in schizophrenia. *Social Cognitive and Affective Neuroscience*, *11*(3), 445–457. doi: 10.1093/scan/nsv125

Malejko, K., Hafner, S., Plener, P. L., Bonenberger, M., Groen, G., Abler, B., & Graf, H. (2021). Neural signature of error processing in major depression. *European Archives of Psychiatry and Clinical Neuroscience*, *271*(7), 1359–1368. doi: 10.1007/s00406-021-01238-y

Malhi, G. S., Lagopoulos, J., Das, P., Moss, K., Berk, M., & Coulston, C. M. (2008). A functional MRI study of Theory of Mind in euthymic bipolar disorder patients. *Bipolar Disorders*, *10*(8), 943–956. doi: 10.1111/j.1399-5618.2008.00643.x

Malhi, G. S., Lagopoulos, J., Owen, A. M., Ivanovski, B., Shnier, R., & Sachdev, P. (2007). Reduced activation to implicit affect induction in euthymic bipolar patients: An fMRI study. *Journal of Affective Disorders*, *97*(1–3), 109–122. doi: 10.1016/j.jad.2006.06.005

Malhi, G. S., Lagopoulos, J., Sachdev, P. S., Ivanovski, B., & Shnier, R. (2005). An emotional Stroop functional MRI study of euthymic bipolar disorder. *Bipolar Disorders*, *7*(s5), 58–69. doi: 10.1111/j.1399-5618.2005.00255.x

Malhi, G. S., Lagopoulos, J., Sachdev, P. S., Ivanovski, B., Shnier, R., & Ketter, T. (2007). Is a lack of disgust something to fear? A functional magnetic resonance imaging facial emotion recognition study in euthymic bipolar disorder patients. *Bipolar Disorders*, *9*(4), 345–357. doi: 10.1111/j.1399-5618.2007.00485.x

Manoach, D. S., White, N., Lindgren, K. A., Heckers, S., Coleman, M. J., Dubal, S., … Holzman, P. S. (2005). Intact hemispheric specialization for spatial and shape working memory in schizophrenia. *Schizophrenia Research*, *78*(1), 1–12. doi: 10.1016/j.schres.2005.06.017

Marchand, W. R., Lee, J. N., Garn, C., Thatcher, J., Gale, P., Kreitschitz, S., … Wood, N. (2011). Aberrant emotional processing in posterior cortical midline structures in bipolar II depression. *Progress in Neuro-Psychopharmacology and Biological Psychiatry*, *35*(7), 1729–1737. doi: 10.1016/j.pnpbp.2011.05.017

Martinez, A., Hillyard, S. A., Bickel, S., Dias, E. C., Butler, P. D., & Javitt, D. C. (2012). Consequences of Magnocellular Dysfunction on Processing Attended Information in Schizophrenia. *Cerebral Cortex*, *22*(6), 1282–1293. doi: 10.1093/cercor/bhr195

Martin-Subero, M., Fuentes-Claramonte, P., Salgado-Pineda, P., Salavert, J., Arevalo, A., Bosque, C., … Pomarol-Clotet, E. (2021). Autobiographical memory and default mode network function in schizophrenia: An fMRI study—CORRIGENDUM. *Psychological Medicine*, *51*(1), 129–129. doi: 10.1017/S0033291720001361

Matsuo, K, Glahn, D. C., Peluso, M. A. M., Hatch, J. P., Monkul, E. S., Najt, P., … Soares, J. C. (2007). Prefrontal hyperactivation during working memory task in untreated individuals with major depressive disorder. *Molecular Psychiatry*, *12*(2), 158–166. doi: 10.1038/sj.mp.4001894

Matsuo, Kayako, Chen, S.-H. A., Liu, C.-M., Liu, C.-C., Hwang, T.-J., Hsieh, M. H., … Tseng, W.-Y. I. (2013). Stable signatures of schizophrenia in the cortical–subcortical–cerebellar network using fMRI of verbal working memory. *Schizophrenia Research*, *151*(1–3), 133–140. doi: 10.1016/j.schres.2013.10.028

Matthews, S., Simmons, A., Strigo, I., Gianaros, P., Yang, T., & Paulus, M. (2009). Inhibition-related activity in subgenual cingulate is associated with symptom severity in major depression. *Psychiatry Research: Neuroimaging*, *172*(1), 1–6. doi: 10.1016/j.pscychresns.2008.08.006

Mazzola-Pomietto, P., Kaladjian, A., Azorin, J.-M., Anton, J.-L., & Jeanningros, R. (2009). Bilateral decrease in ventrolateral prefrontal cortex activation during motor response inhibition in mania. *Journal of Psychiatric Research*, *43*(4), 432–441. doi: 10.1016/j.jpsychires.2008.05.004

McAllindon, D. P., Wilman, A. H., Purdon, S. E., & Tibbo, P. G. (2010). Functional magnetic resonance imaging of choice reaction time in chronic schizophrenia and first-degree relatives. *Schizophrenia Research*, *120*(1–3), 232–233. doi: 10.1016/j.schres.2010.01.015

McGrath, H., Zaveri, H. P., Collins, E., Jafar, T., Chishti, O., Obaid, S., … Spencer, D. D. (2022). High-resolution cortical parcellation based on conserved brain landmarks for localization of multimodal data to the nearest centimeter. *Scientific Reports*, *12*(1), 18778. doi: 10.1038/s41598-022-21543-3

McIntosh, A. M., Whalley, H. C., McKirdy, J., Hall, J., Sussmann, J. E. D., Shankar, P., … Lawrie, S. M. (2008). Prefrontal Function and Activation in Bipolar Disorder and Schizophrenia. *American Journal of Psychiatry*, *165*(3), 378–384. doi: 10.1176/appi.ajp.2007.07020365

McKenna, B. S., Sutherland, A. N., Legenkaya, A. P., & Eyler, L. T. (2014). Abnormalities of brain response during encoding into verbal working memory among euthymic patients with bipolar disorder. *Bipolar Disorders*, *16*(3), 289–299. doi: 10.1111/bdi.12126

Meisenzahl, E. M., Scheuerecker, J., Zipse, M., Ufer, S., Wiesmann, M., Frodl, T., … Möller, H. J. (2006). Effects of treatment with the atypical neuroleptic quetiapine on working memory function: A functional MRI follow-up investigation. *European Archives of Psychiatry and Clinical Neuroscience*, *256*(8), 522–531. doi: 10.1007/s00406-006-0687-x

Mendrek, A., Bourque, J., Dubé, A., Lakis, N., & Champagne, J. (2012). Emotion Processing in Women with Schizophrenia Is Menstrual Cycle Phase and Affective Valence Dependent: An fMRI Study. *ISRN Psychiatry*, *2012*, 1–13. doi: 10.5402/2012/656274

Meusel, L.-A. C., Hall, G. B. C., Fougere, P., McKinnon, M. C., & MacQueen, G. M. (2013). Neural correlates of cognitive remediation in patients with mood disorders. *Psychiatry Research: Neuroimaging*, *214*(2), 142–152. doi: 10.1016/j.pscychresns.2013.06.007

Michalopoulou, P. G., Surguladze, S., Morley, L. A., Giampietro, V. P., Murray, R. M., & Shergill, S. S. (2008). Facial fear processing and psychotic symptoms in schizophrenia: Functional magnetic resonance imaging study. *British Journal of Psychiatry*, *192*(3), 191–196. doi: 10.1192/bjp.bp.106.032649

Mier, D., Sauer, C., Lis, S., Esslinger, C., Wilhelm, J., Gallhofer, B., & Kirsch, P. (2010). Neuronal correlates of affective theory of mind in schizophrenia out-patients: Evidence for a baseline deficit. *Psychological Medicine*, *40*(10), 1607–1617. doi: 10.1017/S0033291709992133

Mier, Daniela, Lis, S., Zygrodnik, K., Sauer, C., Ulferts, J., Gallhofer, B., & Kirsch, P. (2014). Evidence for altered amygdala activation in schizophrenia in an adaptive emotion recognition task. *Psychiatry Research: Neuroimaging*, *221*(3), 195–203. doi: 10.1016/j.pscychresns.2013.12.001

Mingtian, Z., Shuqiao, Y., Xiongzhao, Z., Jinyao, Y., Xueling, Z., Xiang, W., … Wei, W. (2012). Elevated amygdala activity to negative faces in young adults with early onset major depressive disorder. *Psychiatry Research: Neuroimaging*, *201*(2), 107–112. doi: 10.1016/j.pscychresns.2011.06.003

Mitterschiffthaler, Martina T., Kumari, V., Malhi, G. S., Brown, R. G., Giampietro, V. P., Brammer, M. J., … Sharma, T. (2003). Neural response to pleasant stimuli in anhedonia: An fMRI study: *NeuroReport*, *14*(2), 177–182. doi: 10.1097/00001756-200302100-00003

Mitterschiffthaler, M. T., Williams, S. C. R., Walsh, N. D., Cleare, A. J., Donaldson, C., Scott, J., & Fu, C. H. Y. (2008). Neural basis of the emotional Stroop interference effect in major depression. *Psychological Medicine*, *38*(2), 247–256. doi: 10.1017/S0033291707001523

Monks, P. J., Thompson, J. M., Bullmore, E. T., Suckling, J., Brammer, M. J., Williams, S. C., … Curtis, V. A. (2004). A functional MRI study of working memory task in euthymic bipolar disorder: Evidence for task-specific dysfunction. *Bipolar Disorders*, *6*(6), 550–564. doi: 10.1111/j.1399-5618.2004.00147.x

Moran, E. K., Culbreth, A. J., Kandala, S., & Barch, D. M. (2019). From neuroimaging to daily functioning: A multimethod analysis of reward anticipation in people with schizophrenia. *Journal of Abnormal Psychology*, *128*(7), 723–734. doi: 10.1037/abn0000461

Morris, R. W., Vercammen, A., Lenroot, R., Moore, L., Langton, J. M., Short, B., … Weickert, T. W. (2012). Disambiguating ventral striatum fMRI-related bold signal during reward prediction in schizophrenia. *Molecular Psychiatry*, *17*(3), 280–289. doi: 10.1038/mp.2011.75

Moser, D. A., Doucet, G. E., Lee, W. H., Rasgon, A., Krinsky, H., Leibu, E., … Frangou, S. (2018). Multivariate Associations Among Behavioral, Clinical, and Multimodal Imaging Phenotypes in Patients With Psychosis. *JAMA Psychiatry*, *75*(4), 386. doi: 10.1001/jamapsychiatry.2017.4741

Mothersill, O., Morris, D. W., Kelly, S., Rose, E. J., Bokde, A., Reilly, R., … Donohoe, G. (2014). Altered medial prefrontal activity during dynamic face processing in schizophrenia spectrum patients. *Schizophrenia Research*, *157*(1–3), 225–230. doi: 10.1016/j.schres.2014.05.023

Mukherjee, P., Whalley, H. C., McKirdy, J. W., Sprengelmeyer, R., Young, A. W., McIntosh, A. M., … Hall, J. (2014a). Altered Amygdala Connectivity Within the Social Brain in Schizophrenia. *Schizophrenia Bulletin*, *40*(1), 152–160. doi: 10.1093/schbul/sbt086

Mukherjee, P., Whalley, H. C., McKirdy, J. W., Sprengelmeyer, R., Young, A. W., McIntosh, A. M., … Hall, J. (2014b). Altered Amygdala Connectivity Within the Social Brain in Schizophrenia. *Schizophrenia Bulletin*, *40*(1), 152–160. doi: 10.1093/schbul/sbt086

Murphy, E. R., Brent, B. K., Benton, M., Pruitt, P., Diwadkar, V., Rajarethinam, R. P., & Keshavan, M. S. (2010). Differential processing of metacognitive evaluation and the neural circuitry of the self and others in schizophrenia: A pilot study. *Schizophrenia Research*, *116*(2–3), 252–258. doi: 10.1016/j.schres.2009.11.009

Murrough, J. W., Collins, K. A., Fields, J., DeWilde, K. E., Phillips, M. L., Mathew, S. J., … Iosifescu, D. V. (2015). Regulation of neural responses to emotion perception by ketamine in individuals with treatment-resistant major depressive disorder. *Translational Psychiatry*, *5*(2), e509–e509. doi: 10.1038/tp.2015.10

Nagel, M., Sprenger, A., Steinlechner, S., Binkofski, F., & Lencer, R. (2012). Altered Velocity Processing in Schizophrenia during Pursuit Eye Tracking. *PLoS ONE*, *7*(6), e38494. doi: 10.1371/journal.pone.0038494

Nagy, S. A., Kürtös, Z., Németh, N., Perlaki, G., Csernela, E., Lakner, F. E., … Simon, M. (2021). Childhood maltreatment results in altered deactivation of reward processing circuits in depressed patients: A functional magnetic resonance imaging study of a facial emotion recognition task. *Neurobiology of Stress*, *15*, 100399. doi: 10.1016/j.ynstr.2021.100399

Naismith, S. L., Lagopoulos, J., Ward, P. B., Davey, C. G., Little, C., & Hickie, I. B. (2010). Fronto-striatal correlates of impaired implicit sequence learning in major depression: An fMRI study. *Journal of Affective Disorders*, *125*(1–3), 256–261. doi: 10.1016/j.jad.2010.02.114

Negoias, S., Chen, B., Iannilli, E., Ning, Y., Kitzler, H. H., Hummel, T., & Krüger, S. (2019). Odor-related brain hyper-reactivity in euthymic bipolar disorder: An fMRI and ERP study. *Psychiatry Research*, *278*, 218–227. doi: 10.1016/j.psychres.2019.06.016

Ngan, E. T. C., Vouloumanos, A., Cairo, T. A., Laurens, K. R., Bates, A. T., Anderson, C. M., … Liddle, P. F. (2003). Abnormal processing of speech during oddball target detection in schizophrenia. *NeuroImage*, *20*(2), 889–897. doi: 10.1016/S1053-8119(03)00385-9

Nichols, E. S., Penner, J., Ford, K. A., Wammes, M., Neufeld, R. W. J., Mitchell, D. G. V., … Osuch, E. A. (2021). Emotion regulation in emerging adults with major depressive disorder and frequent cannabis use. *NeuroImage: Clinical*, *30*, 102575. doi: 10.1016/j.nicl.2021.102575

Norbury, R., Selvaraj, S., Taylor, M. J., Harmer, C., & Cowen, P. J. (2010). Increased neural response to fear in patients recovered from depression: A 3T functional magnetic resonance imaging study. *Psychological Medicine*, *40*(3), 425–432. doi: 10.1017/S0033291709990596

Oertel, V., Kraft, D., Alves, G., Knöchel, C., Ghinea, D., Storchak, H., … Stäblein, M. (2019). Associative Memory Impairments Are Associated With Functional Alterations Within the Memory Network in Schizophrenia Patients and Their Unaffected First-Degree Relatives: An fMRI Study. *Frontiers in Psychiatry*, *10*, 33. doi: 10.3389/fpsyt.2019.00033

Oertel-Knöchel, V., Reinke, B., Feddern, R., Knake, A., Knöchel, C., Prvulovic, D., … Linden, D. E. (2014). Episodic memory impairments in bipolar disorder are associated with functional and structural brain changes. *Bipolar Disorders*, *16*(8), 830–845. doi: 10.1111/bdi.12241

Oertel-Knöchel, V., Reinke, B., Feddern, R., Knake, A., Knöchel, C., Prvulovic, D., … Linden, D. E. J. (2013). Verbal episodic memory deficits in remitted bipolar patients: A combined behavioural and fMRI study. *Journal of Affective Disorders*, *150*(2), 430–440. doi: 10.1016/j.jad.2013.04.036

Oh, J., Chun, J.-W., Joon Jo, H., Kim, E., Park, H.-J., Lee, B., & Kim, J.-J. (2015). The neural basis of a deficit in abstract thinking in patients with schizophrenia. *Psychiatry Research: Neuroimaging*, *234*(1), 66–73. doi: 10.1016/j.pscychresns.2015.08.007

Okruszek, Ł., Wordecha, M., Jarkiewicz, M., Kossowski, B., Lee, J., & Marchewka, A. (2018a). Brain correlates of recognition of communicative interactions from biological motion in schizophrenia. *Psychological Medicine*, *48*(11), 1862–1871. doi: 10.1017/S0033291717003385

Okruszek, Ł., Wordecha, M., Jarkiewicz, M., Kossowski, B., Lee, J., & Marchewka, A. (2018b). Brain correlates of recognition of communicative interactions from biological motion in schizophrenia. *Psychological Medicine*, *48*(11), 1862–1871. doi: 10.1017/S0033291717003385

Öngür, D., Cullen, T. J., Wolf, D. H., Rohan, M., Barreira, P., Zalesak, M., & Heckers, S. (2006). The Neural Basis of Relational Memory Deficits in Schizophrenia. *Archives of General Psychiatry*, *63*(4), 356. doi: 10.1001/archpsyc.63.4.356

Overbeek, G., Gawne, T. J., Reid, M. A., Salibi, N., Kraguljac, N. V., White, D. M., & Lahti, A. C. (2019). Relationship Between Cortical Excitation and Inhibition and Task-Induced Activation and Deactivation: A Combined Magnetic Resonance Spectroscopy and Functional Magnetic Resonance Imaging Study at 7T in First-Episode Psychosis. *Biological Psychiatry: Cognitive Neuroscience and Neuroimaging*, *4*(2), 121–130. doi: 10.1016/j.bpsc.2018.10.002

Pae, C.-U., Juh, R., Yoo, S.-S., Choi, B.-G., Lim, H.-K., Lee, C., … Lee, C.-U. (2008). Verbal Working Memory Dysfunction in Schizophrenia: An fMRI Investigation. *International Journal of Neuroscience*, *118*(10), 1467–1487. doi: 10.1080/00207450701591131

Panagiotaropoulou, G., Thrapsanioti, E., Pappa, E., Grigoras, C., Mylonas, D., Karavasilis, E., … Smyrnis, N. (2019). Hypo-activity of the dorsolateral prefrontal cortex relates to increased reaction time variability in patients with schizophrenia. *NeuroImage: Clinical*, *23*, 101853. doi: 10.1016/j.nicl.2019.101853

Papagni, S. A., Mechelli, A., Prata, D. P., Kambeitz, J., Fu, C. H. Y., Picchioni, M., … McGuire, P. (2011). Differential effects of DAAO on regional activation and functional connectivity in schizophrenia, bipolar disorder and controls. *NeuroImage*, *56*(4), 2283–2291. doi: 10.1016/j.neuroimage.2011.03.037

Park, H., Kirlic, N., Kuplicki, R., Paulus, M., Guinjoan, S., Aupperle, R., … Victor, T. A. (2022). Neural Processing Dysfunctions During Fear Learning but Not Reward-Related Processing Characterize Depressed Individuals With High Levels of Repetitive Negative Thinking. *Biological Psychiatry: Cognitive Neuroscience and Neuroimaging*, *7*(7), 716–724. doi: 10.1016/j.bpsc.2022.01.002

Park, J., Chun, J.-W., Park, H.-J., Kim, E., & Kim, J.-J. (2018). Involvement of amygdala-prefrontal dysfunction in the influence of negative emotion on the resolution of cognitive conflict in patients with schizophrenia. *Brain and Behavior*, *8*(8), e01064. doi: 10.1002/brb3.1064

Park, J.-I., Kim, G.-W., Jeong, G.-W., & Yang, J.-C. (2019). Brain Activation Patterns Associated with the Effects of Fearful Distractors during Working Memory Maintenance in Patients with Schizophrenia. *Clinical Psychopharmacology and Neuroscience*, *17*(1), 54–63. doi: 10.9758/cpn.2019.17.1.54

Park, K.-M., Kim, J.-J., Ku, J., Kim, S. Y., Lee, H. R., Kim, S. I., & Yoon, K.-J. (2009). Neural basis of attributional style in schizophrenia. *Neuroscience Letters*, *459*(1), 35–40. doi: 10.1016/j.neulet.2009.04.059

Parlar, M., Densmore, M., Hall, G. B. C., Lanius, R., & McKinnon, M. C. (2018). Neural and behavioural correlates of autobiographical memory retrieval in patients with major depressive disorder and a history of trauma exposure. *Neuropsychologia*, *110*, 148–158. doi: 10.1016/j.neuropsychologia.2017.07.004

Pavuluri, M. N., Passarotti, A. M., Harral, E. M., & Sweeney, J. A. (2010). Enhanced Prefrontal Function With Pharmacotherapy on a Response Inhibition Task in Adolescent Bipolar Disorder. *The Journal of Clinical Psychiatry*, *71*(11), 1526–1534. doi: 10.4088/JCP.09m05504yel

Payoux, P., Boulanouar, K., Sarramon, C., Fabre, N., Descombes, S., Galitsky, M., … Rascol, O. (2004). Cortical motor activation in akinetic schizophrenic patients: A pilot functional MRI study. *Movement Disorders*, *19*(1), 83–90. doi: 10.1002/mds.10598

Pedersen, A., Koelkebeck, K., Brandt, M., Wee, M., Kueppers, K. A., Kugel, H., … Ohrmann, P. (2012). Theory of mind in patients with schizophrenia: Is mentalizing delayed? *Schizophrenia Research*, *137*(1–3), 224–229. doi: 10.1016/j.schres.2012.02.022

Pedersen, A., Wilmsmeier, A., Wiedl, K. H., Bauer, J., Kueppers, K., Koelkebeck, K., … Ohrmann, P. (2012). Anterior cingulate cortex activation is related to learning potential on the WCST in schizophrenia patients. *Brain and Cognition*, *79*(3), 245–251. doi: 10.1016/j.bandc.2012.03.007

Penfold, C., Vizueta, N., Townsend, J. D., Bookheimer, S. Y., & Altshuler, L. L. (2015). Frontal lobe hypoactivation in medication-free adults with bipolar II depression during response inhibition. *Psychiatry Research: Neuroimaging*, *231*(3), 202–209. doi: 10.1016/j.pscychresns.2014.11.005

Perlman, S. B., Almeida, J. R., Kronhaus, D. M., Versace, A., LaBarbara, E. J., Klein, C. R., & Phillips, M. L. (2012). Amygdala activity and prefrontal cortex-amygdala effective connectivity to emerging emotional faces distinguish remitted and depressed mood states in bipolar disorder: Brain activity and connectivity in bipolar disorder. *Bipolar Disorders*, *14*(2), 162–174. doi: 10.1111/j.1399-5618.2012.00999.x

Perlstein, W. M., Dixit, N. K., Carter, C. S., Noll, D. C., & Cohen, J. D. (2003). Prefrontal cortex dysfunction mediates deficits in working memory and prepotent responding in schizophrenia. *Biological Psychiatry*, *53*(1), 25–38. doi: 10.1016/S0006-3223(02)01675-X

Phillips, R. C., Salo, T., & Carter, C. S. (2015). Distinct neural correlates for attention lapses in patients with schizophrenia and healthy participants. *Frontiers in Human Neuroscience*, *9*. doi: 10.3389/fnhum.2015.00502

Pinkham, A. E., Klein, H. S., Hardaway, G. B., Kemp, K. C., & Harvey, P. D. (2018). Neural correlates of social cognitive introspective accuracy in schizophrenia. *Schizophrenia Research*, *202*, 166–172. doi: 10.1016/j.schres.2018.07.001

Pizzagalli, D. A., Holmes, A. J., Dillon, D. G., Goetz, E. L., Birk, J. L., Bogdan, R., … Fava, M. (2009). Reduced Caudate and Nucleus Accumbens Response to Rewards in Unmedicated Individuals With Major Depressive Disorder. *American Journal of Psychiatry*, *166*(6), 702–710. doi: 10.1176/appi.ajp.2008.08081201

Pomarol-Clotet, E., Salvador, R., Sarró, S., Gomar, J., Vila, F., Martínez, Á., … McKenna, P. J. (2008). Failure to deactivate in the prefrontal cortex in schizophrenia: Dysfunction of the default mode network? *Psychological Medicine*, *38*(8), 1185–1193. doi: 10.1017/S0033291708003565

Pomarol-Clotet, Edith, Alonso-Lana, S., Moro, N., Sarró, S., Bonnin, M. C., Goikolea, J. M., … Salvador, R. (2015). Brain functional changes across the different phases of bipolar disorder. *British Journal of Psychiatry*, *206*(2), 136–144. doi: 10.1192/bjp.bp.114.152033

Pomarol-Clotet, Edith, Moro, N., Sarró, S., Goikolea, J. M., Vieta, E., Amann, B., … Salvador, R. (2012). Failure of de-activation in the medial frontal cortex in mania: Evidence for default mode network dysfunction in the disorder. *The World Journal of Biological Psychiatry*, *13*(8), 616–626. doi: 10.3109/15622975.2011.573808

Pompei, F., Jogia, J., Tatarelli, R., Girardi, P., Rubia, K., Kumari, V., & Frangou, S. (2011). Familial and disease specific abnormalities in the neural correlates of the Stroop Task in Bipolar Disorder. *NeuroImage*, *56*(3), 1677–1684. doi: 10.1016/j.neuroimage.2011.02.052

Poppe, A. B., Barch, D. M., Carter, C. S., Gold, J. M., Ragland, J. D., Silverstein, S. M., & MacDonald, A. W. (2016). Reduced Frontoparietal Activity in Schizophrenia Is Linked to a Specific Deficit in Goal Maintenance: A Multisite Functional Imaging Study. *Schizophrenia Bulletin*, *42*(5), 1149–1157. doi: 10.1093/schbul/sbw036

Potvin, S., Bourque, J., Durand, M., Lipp, O., Lalonde, P., Stip, E., … Mendrek, A. (2013). The Neural Correlates of Mental Rotation Abilities in Cannabis-Abusing Patients with Schizophrenia: An fMRI Study. *Schizophrenia Research and Treatment*, *2013*, 1–10. doi: 10.1155/2013/543842

Prata, D. P., Mechelli, A., Picchioni, M., Fu, C. H. Y., Kane, F., Kalidindi, S., … McGuire, P. K. (2011). No association of Disrupted-in-Schizophrenia-1 variation with prefrontal function in patients with schizophrenia and bipolar disorder. *Genes, Brain and Behavior*, *10*(3), 276–285. doi: 10.1111/j.1601-183X.2010.00665.x

Pretus, C., Bergé, D., Guell, X., Pérez, V., & Vilarroya, Ó. (2021). Brain activity and connectivity differences in reward value discrimination during effort computation in schizophrenia. *European Archives of Psychiatry and Clinical Neuroscience*, *271*(4), 647–659. doi: 10.1007/s00406-020-01145-8

Quevedo, K., Harms, M., Sauder, M., Scott, H., Mohamed, S., Thomas, K. M., … Smyda, G. (2018). The neurobiology of self face recognition among depressed adolescents. *Journal of Affective Disorders*, *229*, 22–31. doi: 10.1016/j.jad.2017.12.023

Ragland, J. Daniel, Gur, R. C., Valdez, J. N., Loughead, J., Elliott, M., Kohler, C., … Gur, R. E. (2005). Levels-of-Processing Effect on Frontotemporal Function in Schizophrenia During Word Encoding and Recognition. *American Journal of Psychiatry*, *162*(10), 1840–1848. doi: 10.1176/appi.ajp.162.10.1840

Ragland, J. Daniel, Gur, R. C., Valdez, J., Turetsky, B. I., Elliott, M., Kohler, C., … Gur, R. E. (2004). Event-Related fMRI of Frontotemporal Activity During Word Encoding and Recognition in Schizophrenia. *American Journal of Psychiatry*, *161*(6), 1004–1015. doi: 10.1176/appi.ajp.161.6.1004

Ragland, J. Daniel, Ranganath, C., Harms, M. P., Barch, D. M., Gold, J. M., Layher, E., … Carter, C. S. (2015). Functional and Neuroanatomic Specificity of Episodic Memory Dysfunction in Schizophrenia: A Functional Magnetic Resonance Imaging Study of the Relational and Item-Specific Encoding Task. *JAMA Psychiatry*, *72*(9), 909. doi: 10.1001/jamapsychiatry.2015.0276

Ragland, J. Daniel, Valdez, J. N., Loughead, J., Gur, R. C., & Gur, R. E. (2006). Functional magnetic resonance imaging of internal source monitoring in schizophrenia: Recognition with and without recollection. *Schizophrenia Research*, *87*(1–3), 160–171. doi: 10.1016/j.schres.2006.05.008

Ragland, J.D., Layher, E., Hannula, D. E., Niendam, T. A., Lesh, T. A., Solomon, M., … Ranganath, C. (2017). Impact of schizophrenia on anterior and posterior hippocampus during memory for complex scenes. *NeuroImage: Clinical*, *13*, 82–88. doi: 10.1016/j.nicl.2016.11.017

Ragland, J.D., Moelter, S. T., Bhati, M. T., Valdez, J. N., Kohler, C. G., Siegel, S. J., … Gur, R. E. (2008). Effect of retrieval effort and switching demand on fMRI activation during semantic word generation in schizophrenia. *Schizophrenia Research*, *99*(1–3), 312–323. doi: 10.1016/j.schres.2007.11.017

Ragland, John D., Blumenfeld, R. S., Ramsay, I. S., Yonelinas, A., Yoon, J., Solomon, M., … Ranganath, C. (2012). Neural correlates of relational and item-specific encoding during working and long-term memory in schizophrenia. *NeuroImage*, *59*(2), 1719–1726. doi: 10.1016/j.neuroimage.2011.08.055

Rai, S., Griffiths, K., Breukelaar, I. A., Barreiros, A. R., Chen, W., Boyce, P., … Korgaonkar, M. S. (2021). Investigating neural circuits of emotion regulation to distinguish euthymic patients with bipolar disorder and major depressive disorder. *Bipolar Disorders*, *23*(3), 284–294. doi: 10.1111/bdi.13042

Rametti, G., Junqué, C., Vendrell, P., Catalán, R., Penadés, R., Bargalló, N., & Bernardo, M. (2009). Hippocampal underactivation in an fMRI study of word and face memory recognition in schizophrenia. *European Archives of Psychiatry and Clinical Neuroscience*, *259*(4), 203–211. doi: 10.1007/s00406-008-0852-5

Rao, J. A., Kassel, M. T., Weldon, A. L., Avery, E. T., Briceno, E. M., Mann, M., … Weisenbach, S. L. (2015). The double burden of age and major depressive disorder on the cognitive control network. *Psychology and Aging*, *30*(2), 475–485. doi: 10.1037/pag0000027

Rapp, A. M., Langohr, K., Mutschler, D. E., Klingberg, S., Wild, B., & Erb, M. (2013). Isn’t it ironic? Neural Correlates of Irony Comprehension in Schizophrenia. *PLoS ONE*, *8*(9), e74224. doi: 10.1371/journal.pone.0074224

Rasetti, R., Mattay, V. S., White, M. G., Sambataro, F., Podell, J. E., Zoltick, B., … Weinberger, D. R. (2014). Altered Hippocampal-Parahippocampal Function During Stimulus Encoding: A Potential Indicator of Genetic Liability for Schizophrenia. *JAMA Psychiatry*, *71*(3), 236. doi: 10.1001/jamapsychiatry.2013.3911

Rasetti, R., Mattay, V. S., Wiedholz, L. M., Kolachana, B. S., Hariri, A. R., Callicott, J. H., … Weinberger, D. R. (2009). Evidence That Altered Amygdala Activity in Schizophrenia Is Related to Clinical State and Not Genetic Risk. *American Journal of Psychiatry*, *166*(2), 216–225. doi: 10.1176/appi.ajp.2008.08020261

Raucher‐Chéné, D., Obert, A., Gierski, F., Benzerouk, F., Terrien, S., Barrière, S., … Kaladjian, A. (2021). Neural correlates of semantic ambiguity resolution in paucisymptomatic bipolar disorder patients. *Psychiatry Research: Neuroimaging*, *316*, 111346. doi: 10.1016/j.pscychresns.2021.111346

Rauer, L., Trost, S., Petrovic, A., & Gruber, O. (2021). Cortical activation abnormalities in bipolar and schizophrenia patients in a combined oddball–incongruence paradigm. *European Archives of Psychiatry and Clinical Neuroscience*, *271*(8), 1487–1499. doi: 10.1007/s00406-020-01168-1

Razafimandimby, A., Hervé, P.-Y., Marzloff, V., Brazo, P., Tzourio-Mazoyer, N., & Dollfus, S. (2016). Functional deficit of the medial prefrontal cortex during emotional sentence attribution in schizophrenia. *Schizophrenia Research*, *178*(1–3), 86–93. doi: 10.1016/j.schres.2016.09.004

Regenbogen, C., Kellermann, T., Seubert, J., Schneider, D. A., Gur, R. E., Derntl, B., … Habel, U. (2015). Neural responses to dynamic multimodal stimuli and pathology-specific impairments of social cognition in schizophrenia and depression. *British Journal of Psychiatry*, *206*(3), 198–205. doi: 10.1192/bjp.bp.113.143040

Reid, M. A., Stoeckel, L. E., White, D. M., Avsar, K. B., Bolding, M. S., Akella, N. S., … Lahti, A. C. (2010). Assessments of Function and Biochemistry of the Anterior Cingulate Cortex in Schizophrenia. *Biological Psychiatry*, *68*(7), 625–633. doi: 10.1016/j.biopsych.2010.04.013

Reinen, J. M., Van Snellenberg, J. X., Horga, G., Abi-Dargham, A., Daw, N. D., & Shohamy, D. (2016). Motivational Context Modulates Prediction Error Response in Schizophrenia. *Schizophrenia Bulletin*, *42*(6), 1467–1475. doi: 10.1093/schbul/sbw045

Reinen, J. M., Whitton, A. E., Pizzagalli, D. A., Slifstein, M., Abi-Dargham, A., McGrath, P. J., … Schneier, F. R. (2021). Differential reinforcement learning responses to positive and negative information in unmedicated individuals with depression. *European Neuropsychopharmacology*, *53*, 89–100. doi: 10.1016/j.euroneuro.2021.08.002

Reiss, J. P., Campbell, D. W., Leslie, W. D., Paulus, M. P., Ryner, L. N., Polimeni, J. O., … Sareen, J. (2006). Deficit in schizophrenia to recruit the striatum in implicit learning: A functional magnetic resonance imaging investigation. *Schizophrenia Research*, *87*(1–3), 127–137. doi: 10.1016/j.schres.2006.04.027

Remijnse, P. L., Nielen, M. M. A., Van Balkom, A. J. L. M., Hendriks, G.-J., Hoogendijk, W. J., Uylings, H. B. M., & Veltman, D. J. (2009). Differential frontal–striatal and paralimbic activity during reversal learning in major depressive disorder and obsessive–compulsive disorder. *Psychological Medicine*, *39*(9), 1503–1518. doi: 10.1017/S0033291708005072

Rey, G., Desseilles, M., Favre, S., Dayer, A., Piguet, C., Aubry, J.-M., & Vuilleumier, P. (2014). Modulation of brain response to emotional conflict as a function of current mood in bipolar disorder: Preliminary findings from a follow-up state-based fMRI study. *Psychiatry Research: Neuroimaging*, *223*(2), 84–93. doi: 10.1016/j.pscychresns.2014.04.016

Richter, A., Petrovic, A., Diekhof, E. K., Trost, S., Wolter, S., & Gruber, O. (2015). Hyperresponsivity and impaired prefrontal control of the mesolimbic reward system in schizophrenia. *Journal of Psychiatric Research*, *71*, 8–15. doi: 10.1016/j.jpsychires.2015.09.005

Ritchey, M., Dolcos, F., Eddington, K. M., Strauman, T. J., & Cabeza, R. (2011). Neural correlates of emotional processing in depression: Changes with cognitive behavioral therapy and predictors of treatment response. *Journal of Psychiatric Research*, *45*(5), 577–587. doi: 10.1016/j.jpsychires.2010.09.007

Rizvi, S. J., Salomons, T. V., Konarski, J. Z., Downar, J., Giacobbe, P., McIntyre, R. S., & Kennedy, S. H. (2013). Neural response to emotional stimuli associated with successful antidepressant treatment and behavioral activation. *Journal of Affective Disorders*, *151*(2), 573–581. doi: 10.1016/j.jad.2013.06.050

Robinson, J. L., Bearden, C. E., Monkul, E. S., Tordesillas-Gutiérrez, D., Velligan, D. I., Frangou, S., & Glahn, D. C. (2009). Fronto-temporal dysregulation in remitted bipolar patients: An fMRI delayed-non-match-to-sample (DNMS) study. *Bipolar Disorders*, *11*(4), 351–360. doi: 10.1111/j.1399-5618.2009.00703.x

Robinson, O. J., Cools, R., Carlisi, C. O., Sahakian, B. J., & Drevets, W. C. (2012). Ventral Striatum Response During Reward and Punishment Reversal Learning in Unmedicated Major Depressive Disorder. *American Journal of Psychiatry*, *169*(2), 152–159. doi: 10.1176/appi.ajp.2011.11010137

Rodrigue, A. L., Schaeffer, D. J., Pierce, J. E., Clementz, B. A., & McDowell, J. E. (2018). Evaluating the Specificity of Cognitive Control Deficits in Schizophrenia Using Antisaccades, Functional Magnetic Resonance Imaging, and Healthy Individuals With Poor Cognitive Control. *Frontiers in Psychiatry*, *9*, 107. doi: 10.3389/fpsyt.2018.00107

Rodríguez-Cano, E., Sarró, S., Monté, G. C., Maristany, T., Salvador, R., McKenna, P. J., & Pomarol-Clotet, E. (2014). Evidence for structural and functional abnormality in the subgenual anterior cingulate cortex in major depressive disorder. *Psychological Medicine*, *44*(15), 3263–3273. doi: 10.1017/S0033291714000841

Rodríguez-Cano, Elena, Alonso-Lana, S., Sarró, S., Fernández-Corcuera, P., Goikolea, J. M., Vieta, E., … Pomarol-Clotet, E. (2017). Differential failure to deactivate the default mode network in unipolar and bipolar depression. *Bipolar Disorders*, *19*(5), 386–395. doi: 10.1111/bdi.12517

Rolls, E. T., Huang, C.-C., Lin, C.-P., Feng, J., & Joliot, M. (2020). Automated anatomical labelling atlas 3. *NeuroImage*, *206*, 116189. doi: 10.1016/j.neuroimage.2019.116189

Rose, E. J., Simonotto, E., & Ebmeier, K. P. (2006). Limbic over-activity in depression during preserved performance on the n-back task. *NeuroImage*, *29*(1), 203–215. doi: 10.1016/j.neuroimage.2005.07.002

Roth, R. M., Koven, N. S., Randolph, J. J., Flashman, L. A., Pixley, H. S., Ricketts, S. M., … Saykin, A. J. (2006). Functional magnetic resonance imaging of executive control in bipolar disorder. *NeuroReport*, *17*(11), 1085–1089. doi: 10.1097/01.wnr.0000227979.06013.57

Rowland, L. M., Griego, J. A., Spieker, E. A., Cortes, C. R., & Holcomb, H. H. (2010). Neural Changes Associated With Relational Learning in Schizophrenia. *Schizophrenia Bulletin*, *36*(3), 496–503. doi: 10.1093/schbul/sbq037

Roybal, D. J., Cosgrove, V. E., Kelley, R., Smallwood Shoukry, R., Larios, R. M., Novy, B., … Garrett, A. S. (2022). Aberrant Neural Response to Social Exclusion Without Significantly Greater Distress in Youth With Bipolar Disorder: Preliminary Findings. *Frontiers in Psychiatry*, *13*, 687052. doi: 10.3389/fpsyt.2022.687052

Royer, A., Schneider, F. C. G., Grosselin, A., Pellet, J., Barral, F.-G., Laurent, B., … Lang, F. (2009). Brain activation during executive processes in schizophrenia. *Psychiatry Research: Neuroimaging*, *173*(3), 170–176. doi: 10.1016/j.pscychresns.2009.02.009

Rubia, K., Russell, T., Bullmore, E. T., Soni, W., Brammer, M. J., Simmons, A., … Sharma, T. (2001). An fMRI study of reduced left prefrontal activation in schizophrenia during normal inhibitory function. *Schizophrenia Research*, *52*(1–2), 47–55. doi: 10.1016/S0920-9964(00)00173-0

Russell, T. A., Rubia, K., Bullmore, E. T., Soni, W., Suckling, J., Brammer, M. J., … Sharma, T. (2000). Exploring the Social Brain in Schizophrenia: Left Prefrontal Underactivation During Mental State Attribution. *American Journal of Psychiatry*, *157*(12), 2040–2042. doi: 10.1176/appi.ajp.157.12.2040

Rütgen, M., Pletti, C., Tik, M., Kraus, C., Pfabigan, D. M., Sladky, R., … Lamm, C. (2019). Antidepressant treatment, not depression, leads to reductions in behavioral and neural responses to pain empathy. *Translational Psychiatry*, *9*(1), 164. doi: 10.1038/s41398-019-0496-4

Salgado-Pineda, P., Junqué, C., Vendrell, P., Baeza, I., Bargalló, N., Falcón, C., & Bernardo, M. (2004). Decreased cerebral activation during CPT performance. *NeuroImage*, *21*(3), 840–847. doi: 10.1016/j.neuroimage.2003.10.027

Sapara, A., Ffytche, D. H., Birchwood, M., Cooke, M. A., Fannon, D., Williams, S. C. R., … Kumari, V. (2014). Preservation and compensation: The functional neuroanatomy of insight and working memory in schizophrenia. *Schizophrenia Research*, *152*(1), 201–209. doi: 10.1016/j.schres.2013.11.026

Sarsam, M., Parkes, L. M., Roberts, N., Reid, G. S., & Kinderman, P. (2013). The Queen and I: Neural Correlates of Altered Self-Related Cognitions in Major Depressive Episode. *PLoS ONE*, *8*(10), e78844. doi: 10.1371/journal.pone.0078844

Sass, K., Heim, S., Sachs, O., Straube, B., Schneider, F., Habel, U., & Kircher, T. (2014). Neural correlates of semantic associations in patients with schizophrenia. *European Archives of Psychiatry and Clinical Neuroscience*, *264*(2), 143–154. doi: 10.1007/s00406-013-0425-0

Satterthwaite, T. D., Wolf, D. H., Loughead, J., Ruparel, K., Valdez, J. N., Siegel, S. J., … Gur, R. C. (2010). Association of Enhanced Limbic Response to Threat With Decreased Cortical Facial Recognition Memory Response in Schizophrenia. *American Journal of Psychiatry*, *167*(4), 418–426. doi: 10.1176/appi.ajp.2009.09060808

Scheuerecker, J., Ufer, S., Zipse, M., Frodl, T., Koutsouleris, N., Zetzsche, T., … Meisenzahl, E. M. (2008). Cerebral changes and cognitive dysfunctions in medication-free schizophrenia – An fMRI study. *Journal of Psychiatric Research*, *42*(6), 469–476. doi: 10.1016/j.jpsychires.2007.04.001

Scheuerecker, Johanna, Meisenzahl, E. M., Koutsouleris, N., Roesner, M., Schöpf, V., Linn, J., … Frodl, T. (2010). Orbitofrontal volume reductions during emotion recognition in patients with major depression. *Journal of Psychiatry and Neuroscience*, *35*(5), 311–320. doi: 10.1503/jpn.090076

Schlagenhauf, F., Huys, Q. J. M., Deserno, L., Rapp, M. A., Beck, A., Heinze, H.-J., … Heinz, A. (2014). Striatal dysfunction during reversal learning in unmedicated schizophrenia patients. *NeuroImage*, *89*, 171–180. doi: 10.1016/j.neuroimage.2013.11.034

Schlagenhauf, F., Wüstenberg, T., Schmack, K., Dinges, M., Wrase, J., Koslowski, M., … Heinz, A. (2008). Switching schizophrenia patients from typical neuroleptics to olanzapine: Effects on BOLD response during attention and working memory. *European Neuropsychopharmacology*, *18*(8), 589–599. doi: 10.1016/j.euroneuro.2008.04.013

Schlösser, R. G. M., Koch, K., Wagner, G., Nenadic, I., Roebel, M., Schachtzabel, C., … Sauer, H. (2008). Inefficient executive cognitive control in schizophrenia is preceded by altered functional activation during information encoding: An fMRI study. *Neuropsychologia*, *46*(1), 336–347. doi: 10.1016/j.neuropsychologia.2007.07.006

Schneider, F., Habel, U., Reske, M., Kellermann, T., Stöcker, T., Shah, N. J., … Gaebel, W. (2007). Neural correlates of working memory dysfunction in first-episode schizophrenia patients: An fMRI multi-center study. *Schizophrenia Research*, *89*(1–3), 198–210. doi: 10.1016/j.schres.2006.07.021

Schnell, Z., Varga, E., Tényi, T., Simon, M., Hajnal, A., Járai, R., & Herold, R. (2016). Neuropragmatics and irony processing in schizophrenia – Possible neural correlates of the meta-module of pragmatic meaning construction. *Journal of Pragmatics*, *92*, 74–99. doi: 10.1016/j.pragma.2015.11.004

Schöning, S., Zwitserlood, P., Engelien, A., Behnken, A., Kugel, H., Schiffbauer, H., … Konrad, C. (2009). Working-memory fMRI reveals cingulate hyperactivation in euthymic major depression. *Human Brain Mapping*, *30*(9), 2746–2756. doi: 10.1002/hbm.20702

Segarra, N., Metastasio, A., Ziauddeen, H., Spencer, J., Reinders, N. R., Dudas, R. B., … Murray, G. K. (2016). Abnormal Frontostriatal Activity During Unexpected Reward Receipt in Depression and Schizophrenia: Relationship to Anhedonia. *Neuropsychopharmacology*, *41*(8), 2001–2010. doi: 10.1038/npp.2015.370

Seok Jeong, B., Soo Kwon, J., Yoon Kim, S., Lee, C., Youn, T., Moon, C.-H., & Yoon Kim, C. (2005). Functional imaging evidence of the relationship between recurrent psychotic episodes and neurodegenerative course in schizophrenia. *Psychiatry Research: Neuroimaging*, *139*(3), 219–228. doi: 10.1016/j.pscychresns.2004.01.008

Sepede, G., Chiacchiaretta, P., Gambi, F., Di Iorio, G., De Berardis, D., Ferretti, A., … Di Giannantonio, M. (2020). Bipolar disorder with and without a history of psychotic features: fMRI correlates of sustained attention. *Progress in Neuro-Psychopharmacology and Biological Psychiatry*, *98*, 109817. doi: 10.1016/j.pnpbp.2019.109817

Sepede, G., De Berardis, D., Campanella, D., Perrucci, M. G., Ferretti, A., Salerno, R. M., … Gambi, F. (2015). Neural correlates of negative emotion processing in bipolar disorder. *Progress in Neuro-Psychopharmacology and Biological Psychiatry*, *60*, 1–10. doi: 10.1016/j.pnpbp.2015.01.016

Sepede, G., De Berardis, D., Campanella, D., Perrucci, M. G., Ferretti, A., Serroni, N., … Gambi, F. (2012). Impaired sustained attention in euthymic bipolar disorder patients and non-affected relatives: An fMRI study: **Sustained attention in bipolar disorder**. *Bipolar Disorders*, *14*(7), 764–779. doi: 10.1111/bdi.12007

Sergerie, K., Armony, J. L., Menear, M., Sutton, H., & Lepage, M. (2010). Influence of Emotional Expression on Memory Recognition Bias in Schizophrenia as Revealed by fMRI. *Schizophrenia Bulletin*, *36*(4), 800–810. doi: 10.1093/schbul/sbn172

Shad, M. U., Keshavan, M. S., Steinberg, J. L., Mihalakos, P., Thomas, B. P., Motes, M. A., … Tamminga, C. A. (2012). Neurobiology of self-awareness in schizophrenia: An fMRI study. *Schizophrenia Research*, *138*(2–3), 113–119. doi: 10.1016/j.schres.2012.03.016

Shergill, S. S., Bullmore, E., Simmons, A., Murray, R., & McGuire, P. (2000). Functional Anatomy of Auditory Verbal Imagery in Schizophrenic Patients With Auditory Hallucinations. *American Journal of Psychiatry*, *157*(10), 1691–1693. doi: 10.1176/appi.ajp.157.10.1691

Shergill, S. S., White, T. P., Joyce, D. W., Bays, P. M., Wolpert, D. M., & Frith, C. D. (2014). Functional Magnetic Resonance Imaging of Impaired Sensory Prediction in Schizophrenia. *JAMA Psychiatry*, *71*(1), 28. doi: 10.1001/jamapsychiatry.2013.2974

Shi, H., Wang, X., Yi, J., Zhu, X., Zhang, X., Yang, J., & Yao, S. (2015). Default mode network alterations during implicit emotional faces processing in first-episode, treatment-naive major depression patients. *Frontiers in Psychology*, *6*. doi: 10.3389/fpsyg.2015.01198

Shin, N. Y., Park, H. Y., Jung, W. H., Park, J. W., Yun, J.-Y., Jang, J. H., … Kwon, J. S. (2015). Effects of Oxytocin on Neural Response to Facial Expressions in Patients with Schizophrenia. *Neuropsychopharmacology*, *40*(8), 1919–1927. doi: 10.1038/npp.2015.41

Siemerkus, J., Irle, E., Schmidt-Samoa, C., Dechent, P., & Weniger, G. (2012). Egocentric spatial learning in schizophrenia investigated with functional magnetic resonance imaging. *NeuroImage: Clinical*, *1*(1), 153–163. doi: 10.1016/j.nicl.2012.10.004

Silverstein, S. M., All, S. D., Kasi, R., Berten, S., Essex, B., Lathrop, K. L., & Little, D. M. (2010). Increased fusiform area activation in schizophrenia during processing of spatial frequency-degraded faces, as revealed by fMRI. *Psychological Medicine*, *40*(7), 1159–1169. doi: 10.1017/S0033291709991735

Silverstein, Steven M., Berten, S., Essex, B., All, S. D., Kasi, R., & Little, D. M. (2010). Perceptual organization and visual search processes during target detection task performance in schizophrenia, as revealed by fMRI. *Neuropsychologia*, *48*(10), 2886–2893. doi: 10.1016/j.neuropsychologia.2010.05.030

Simons, C. J. P., Tracy, D. K., Sanghera, K. K., O’Daly, O., Gilleen, J., Dominguez, M.-G., … Shergill, S. S. (2010). Functional Magnetic Resonance Imaging of Inner Speech in Schizophrenia. *Biological Psychiatry*, *67*(3), 232–237. doi: 10.1016/j.biopsych.2009.09.007

Singh, M. K., Chang, K. D., Mazaika, P., Garrett, A., Adleman, N., Kelley, R., … Reiss, A. (2010). Neural Correlates of Response Inhibition in Pediatric Bipolar Disorder. *Journal of Child and Adolescent Psychopharmacology*, *20*(1), 15–24. doi: 10.1089/cap.2009.0004

Singh, S., Modi, S., Goyal, S., Kaur, P., Singh, N., Bhatia, T., … Khushu, S. (2015). Functional and structural abnormalities associated with empathy in patients with schizophrenia: An fMRI and VBM study. *Journal of Biosciences*, *40*(2), 355–364. doi: 10.1007/s12038-015-9509-5

Smee, C., Krabbendam, L., O’Daly, O., Prins, A.-M., Nalesnik, N., Morley, L., … Shergill, S. (2011). An fMRI study of prefrontal dysfunction and symptomatic recovery in schizophrenia: An fMRI study of prefrontal dysfunction and symptomatic recovery. *Acta Psychiatrica Scandinavica*, *123*(6), 440–450. doi: 10.1111/j.1600-0447.2010.01632.x

Smieskova, R., Roiser, J. P., Chaddock, C. A., Schmidt, A., Harrisberger, F., Bendfeldt, K., … Borgwardt, S. (2015). Modulation of motivational salience processing during the early stages of psychosis. *Schizophrenia Research*, *166*(1–3), 17–23. doi: 10.1016/j.schres.2015.04.036

Smith, M. J., Schroeder, M. P., Abram, S. V., Goldman, M. B., Parrish, T. B., Wang, X., … Breiter, H. C. (2015). Alterations in Brain Activation During Cognitive Empathy Are Related to Social Functioning in Schizophrenia. *Schizophrenia Bulletin*, *41*(1), 211–222. doi: 10.1093/schbul/sbu023

Smoski, M. J., Felder, J., Bizzell, J., Green, S. R., Ernst, M., Lynch, T. R., & Dichter, G. S. (2009). fMRI of alterations in reward selection, anticipation, and feedback in major depressive disorder. *Journal of Affective Disorders*, *118*(1–3), 69–78. doi: 10.1016/j.jad.2009.01.034

Smoski, M. J., Rittenberg, A., & Dichter, G. S. (2011). Major depressive disorder is characterized by greater reward network activation to monetary than pleasant image rewards. *Psychiatry Research: Neuroimaging*, *194*(3), 263–270. doi: 10.1016/j.pscychresns.2011.06.012

Smucny, J., Lesh, T. A., Newton, K., Niendam, T. A., Ragland, J. D., & Carter, C. S. (2018a). Levels of Cognitive Control: A Functional Magnetic Resonance Imaging-Based Test of an RDoC Domain Across Bipolar Disorder and Schizophrenia. *Neuropsychopharmacology*, *43*(3), 598–606. doi: 10.1038/npp.2017.233

Smucny, J., Lesh, T. A., Newton, K., Niendam, T. A., Ragland, J. D., & Carter, C. S. (2018b). Levels of Cognitive Control: A Functional Magnetic Resonance Imaging-Based Test of an RDoC Domain Across Bipolar Disorder and Schizophrenia. *Neuropsychopharmacology*, *43*(3), 598–606. doi: 10.1038/npp.2017.233

Spaniel, F., Tintera, J., Rydlo, J., Ibrahim, I., Kasparek, T., Horacek, J., … Hajek, T. (2016). Altered Neural Correlate of the Self-Agency Experience in First-Episode Schizophrenia-Spectrum Patients: An fMRI Study. *Schizophrenia Bulletin*, *42*(4), 916–925. doi: 10.1093/schbul/sbv188

Spilka, M. J., Arnold, A. E., & Goghari, V. M. (2015). Functional activation abnormalities during facial emotion perception in schizophrenia patients and nonpsychotic relatives. *Schizophrenia Research*, *168*(1–2), 330–337. doi: 10.1016/j.schres.2015.07.012

Spilka, M. J., & Goghari, V. M. (2017). Similar patterns of brain activation abnormalities during emotional and non-emotional judgments of faces in a schizophrenia family study. *Neuropsychologia*, *96*, 164–174. doi: 10.1016/j.neuropsychologia.2017.01.014

Stäblein, M., Storchak, H., Ghinea, D., Kraft, D., Knöchel, C., Prvulovic, D., … Oertel-Knöchel, V. (2019). Visual working memory encoding in schizophrenia and first-degree relatives: Neurofunctional abnormalities and impaired consolidation. *Psychological Medicine*, *49*(1), 75–83. doi: 10.1017/S003329171800051X

Standke, I., Trempler, I., Dannlowski, U., Schubotz, R. I., & Lencer, R. (2021). Cerebral and behavioral signs of impaired cognitive flexibility and stability in schizophrenia spectrum disorders. *NeuroImage: Clinical*, *32*, 102855. doi: 10.1016/j.nicl.2021.102855

Stegmayer, K., Bohlhalter, S., Vanbellingen, T., Federspiel, A., Wiest, R., Müri, R. M., … Walther, S. (2018). Limbic Interference During Social Action Planning in Schizophrenia. *Schizophrenia Bulletin*, *44*(2), 359–368. doi: 10.1093/schbul/sbx059

Stolz, E., Pancholi, K. M., Goradia, D. D., Paul, S., Keshavan, M. S., Nimgaonkar, V. L., & Prasad, K. M. (2012). Brain activation patterns during visual episodic memory processing among first-degree relatives of schizophrenia subjects. *NeuroImage*, *63*(3), 1154–1161. doi: 10.1016/j.neuroimage.2012.08.030

Strakowski, S. M., Adler, C. M., Cerullo, M. A., Eliassen, J. C., Lamy, M., Fleck, D. E., … DelBello, M. P. (2008). Magnetic resonance imaging brain activation in first-episode bipolar mania during a response inhibition task: Response inhibition in first-episode mania. *Early Intervention in Psychiatry*, *2*(4), 225–233. doi: 10.1111/j.1751-7893.2008.00082.x

Strakowski, S. M., Adler, C. M., Holland, S. K., Mills, N., & DelBello, M. P. (2004). A Preliminary fMRI Study of Sustained Attention in Euthymic, Unmedicated Bipolar Disorder. *Neuropsychopharmacology*, *29*(9), 1734–1740. doi: 10.1038/sj.npp.1300492

Strakowski, S. M., Adler, C. M., Holland, S. K., Mills, N. P., DelBello, M. P., & Eliassen, J. C. (2005). Abnormal fMRI Brain Activation in Euthymic Bipolar Disorder Patients During a Counting Stroop Interference Task. *American Journal of Psychiatry*, *162*(9), 1697–1705. doi: 10.1176/appi.ajp.162.9.1697

Straube, B., Green, A., Sass, K., Kirner-Veselinovic, A., & Kircher, T. (2013). Neural integration of speech and gesture in schizophrenia: Evidence for differential processing of metaphoric gestures. *Human Brain Mapping*, *34*(7), 1696–1712. doi: 10.1002/hbm.22015

Strigo, I A, Matthews, S. C., & Simmons, A. N. (2013). Decreased frontal regulation during pain anticipation in unmedicated subjects with major depressive disorder. *Translational Psychiatry*, *3*(3), e239–e239. doi: 10.1038/tp.2013.15

Strigo, Irina A., Simmons, A. N., Matthews, S. C., Craig, A. D. (Bud), & Paulus, M. P. (2008). Association of Major Depressive Disorder With Altered Functional Brain Response During Anticipation and Processing of Heat Pain. *Archives of General Psychiatry*, *65*(11), 1275. doi: 10.1001/archpsyc.65.11.1275

Sugranyes, G., Kyriakopoulos, M., Dima, D., O’Muircheartaigh, J., Corrigall, R., Pendelbury, G., … Frangou, S. (2012). Multimodal analyses identify linked functional and white matter abnormalities within the working memory network in schizophrenia. *Schizophrenia Research*, *138*(2–3), 136–142. doi: 10.1016/j.schres.2012.03.011

Surguladze, S., Brammer, M. J., Keedwell, P., Giampietro, V., Young, A. W., Travis, M. J., … Phillips, M. L. (2005). A differential pattern of neural response toward sad versus happy facial expressions in major depressive disorder. *Biological Psychiatry*, *57*(3), 201–209. doi: 10.1016/j.biopsych.2004.10.028

Surguladze, S.A., Marshall, N., Schulze, K., Hall, M.-H., Walshe, M., Bramon, E., … McDonald, C. (2010). Exaggerated neural response to emotional faces in patients with bipolar disorder and their first-degree relatives. *NeuroImage*, *53*(1), 58–64. doi: 10.1016/j.neuroimage.2010.05.069

Surguladze, Simon A, Calvert, G. A., Brammer, M. J., Campbell, R., Bullmore, E. T., Giampietro, V., & David, A. S. (2001). Audio–visual speech perception in schizophrenia: An fMRI study. *Psychiatry Research: Neuroimaging*, *106*(1), 1–14. doi: 10.1016/S0925-4927(00)00081-0

Surguladze, Simon A., El-Hage, W., Dalgleish, T., Radua, J., Gohier, B., & Phillips, M. L. (2010). Depression is associated with increased sensitivity to signals of disgust: A functional magnetic resonance imaging study. *Journal of Psychiatric Research*, *44*(14), 894–902. doi: 10.1016/j.jpsychires.2010.02.010

Suttkus, S., Schumann, A., Cruz, F., & Bär, K. (2021). Working memory in schizophrenia: The role of the locus coeruleus and its relation to functional brain networks. *Brain and Behavior*, *11*(5). doi: 10.1002/brb3.2130

Tagamets, M. A., Cortes, C. R., Griego, J. A., & Elvevåg, B. (2014). Neural correlates of the relationship between discourse coherence and sensory monitoring in schizophrenia. *Cortex*, *55*, 77–87. doi: 10.1016/j.cortex.2013.06.011

Takahashi, H., Kato, M., Sassa, T., Shibuya, T., Koeda, M., Yahata, N., … Okubo, Y. (2010). Functional Deficits in the Extrastriate Body Area During Observation of Sports-Related Actions in Schizophrenia. *Schizophrenia Bulletin*, *36*(3), 642–647. doi: 10.1093/schbul/sbn132

Takahashi, Hidehiko, Koeda, M., Oda, K., Matsuda, T., Matsushima, E., Matsuura, M., … Okubo, Y. (2004). An fMRI study of differential neural response to affective pictures in schizophrenia. *NeuroImage*, *22*(3), 1247–1254. doi: 10.1016/j.neuroimage.2004.03.028

Takamura, M., Okamoto, Y., Okada, G., Toki, S., Yamamoto, T., Yamamoto, O., … Yamawaki, S. (2016). Disrupted Brain Activation and Deactivation Pattern during Semantic Verbal Fluency Task in Patients with Major Depression. *Neuropsychobiology*, *74*(2), 69–77. doi: 10.1159/000453399

Tan, H.-Y., Choo, W.-C., Fones, C. S. L., & Chee, M. W. L. (2005). fMRI Study of Maintenance and Manipulation Processes Within Working Memory in First-Episode Schizophrenia. *American Journal of Psychiatry*, *162*(10), 1849–1858. doi: 10.1176/appi.ajp.162.10.1849

Tan, S., Zhao, Y., Fan, F., Zou, Y., Jin, Z., Zen, Y., … Zhou, D. (2015). Brain Correlates of Self-Evaluation Deficits in Schizophrenia: A Combined Functional and Structural MRI Study. *PLOS ONE*, *10*(9), e0138737. doi: 10.1371/journal.pone.0138737

Taylor, S. F., Chen, A. C., Tso, I. F., Liberzon, I., & Welsh, R. C. (2011). Social appraisal in chronic psychosis: Role of medial frontal and occipital networks. *Journal of Psychiatric Research*, *45*(4), 526–538. doi: 10.1016/j.jpsychires.2010.08.004

Tendolkar, I., Weis, S., Guddat, O., Fernández, G., Brockhaus-Dumke, A., Specht, K., … Ruhrmann, S. (2004). Evidence for a dysfunctional retrosplenial cortex in patients with schizophrenia: A functional magnetic resonance imaging study with a semantic—perceptual contrast. *Neuroscience Letters*, *369*(1), 4–8. doi: 10.1016/j.neulet.2004.07.024

Thoresen, C., Endestad, T., Sigvartsen, N. P. B., Server, A., Bolstad, I., Johansson, M., … Jensen, J. (2014). Frontotemporal hypoactivity during a reality monitoring paradigm is associated with delusions in patients with schizophrenia spectrum disorders. *Cognitive Neuropsychiatry*, *19*(2), 97–115. doi: 10.1080/13546805.2013.776495

Tikàsz, A., Dumais, A., Lipp, O., Stip, E., Lalonde, P., Laurelli, M., … Potvin, S. (2019). Reward-related decision-making in schizophrenia: A multimodal neuroimaging study. *Psychiatry Research: Neuroimaging*, *286*, 45–52. doi: 10.1016/j.pscychresns.2019.03.007

Tikàsz, A., Potvin, S., Lungu, O., Joyal, C. C., Hodgins, S., Mendrek, A., & Dumais, A. (2016). Anterior cingulate hyperactivations during negative emotion processing among men with schizophrenia and a history of violent behavior. *Neuropsychiatric Disease and Treatment*, *12*, 1397–1410. doi: 10.2147/NDT.S107545

Townsend, J. D., Bookheimer, S. Y., Foland-Ross, L. C., Moody, T. D., Eisenberger, N. I., Fischer, J. S., … Altshuler, L. L. (2012). Deficits in inferior frontal cortex activation in euthymic bipolar disorder patients during a response inhibition task: Activation deficits in euthymic bipolar disorder patients. *Bipolar Disorders*, *14*(4), 442–450. doi: 10.1111/j.1399-5618.2012.01020.x

Townsend, J. D., Eberhart, N. K., Bookheimer, S. Y., Eisenberger, N. I., Foland-Ross, L. C., Cook, I. A., … Altshuler, L. L. (2010). fMRI activation in the amygdala and the orbitofrontal cortex in unmedicated subjects with major depressive disorder. *Psychiatry Research: Neuroimaging*, *183*(3), 209–217. doi: 10.1016/j.pscychresns.2010.06.001

Townsend, J. D., Torrisi, S. J., Lieberman, M. D., Sugar, C. A., Bookheimer, S. Y., & Altshuler, L. L. (2013). Frontal-Amygdala Connectivity Alterations During Emotion Downregulation in Bipolar I Disorder. *Biological Psychiatry*, *73*(2), 127–135. doi: 10.1016/j.biopsych.2012.06.030

Tozzi, L., Carballedo, A., Wetterling, F., McCarthy, H., O’Keane, V., Gill, M., … Frodl, T. (2016). Single-Nucleotide Polymorphism of the FKBP5 Gene and Childhood Maltreatment as Predictors of Structural Changes in Brain Areas Involved in Emotional Processing in Depression. *Neuropsychopharmacology*, *41*(2), 487–497. doi: 10.1038/npp.2015.170

Tregellas, J. R., Smucny, J., Eichman, L., & Rojas, D. C. (2012). The effect of distracting noise on the neuronal mechanisms of attention in schizophrenia. *Schizophrenia Research*, *142*(1–3), 230–236. doi: 10.1016/j.schres.2012.09.008

Tremblay, L. K., Naranjo, C. A., Graham, S. J., Herrmann, N., Mayberg, H. S., Hevenor, S., & Busto, U. E. (2005). Functional Neuroanatomical Substrates of Altered Reward Processing in Major Depressive Disorder Revealed by a Dopaminergic Probe. *Archives of General Psychiatry*, *62*(11), 1228. doi: 10.1001/archpsyc.62.11.1228

Trettin, M., Dvořák, J., Hilke, M., Wenzler, S., Hagen, M., Ghirmai, N., … Oertel, V. (2022). Neuronal response to high negative affective stimuli in major depressive disorder: An fMRI study. *Journal of Affective Disorders*, *298*, 239–247. doi: 10.1016/j.jad.2021.10.123

Tseng, H.-H., Roiser, J. P., Modinos, G., Falkenberg, I., Samson, C., McGuire, P., & Allen, P. (2016). Corticolimbic dysfunction during facial and prosodic emotional recognition in first-episode psychosis patients and individuals at ultra-high risk. *NeuroImage: Clinical*, *12*, 645–654. doi: 10.1016/j.nicl.2016.09.006

Tseng, W.-L., Thomas, L. A., Harkins, E., Stoddard, J., Zarate, C. A., Pine, D. S., … Brotman, M. A. (2016). Functional connectivity during masked and unmasked face emotion processing in bipolar disorder. *Psychiatry Research: Neuroimaging*, *258*, 1–9. doi: 10.1016/j.pscychresns.2016.10.006

Ungar, L., Nestor, P. G., Niznikiewicz, M. A., Wible, C. G., & Kubicki, M. (2010). Color Stroop and negative priming in schizophrenia: An fMRI study. *Psychiatry Research: Neuroimaging*, *181*(1), 24–29. doi: 10.1016/j.pscychresns.2009.07.005

Ursu, S., Kring, A. M., Gard, M. G., Minzenberg, M. J., Yoon, J. H., Ragland, J. D., … Carter, C. S. (2011). Prefrontal Cortical Deficits and Impaired Cognition-Emotion Interactions in Schizophrenia. *American Journal of Psychiatry*, *168*(3), 276–285. doi: 10.1176/appi.ajp.2010.09081215

Van Der Meer, L., De Vos, A. E., Stiekema, A. P. M., Pijnenborg, G. H. M., Van Tol, M.-J., Nolen, W. A., … Aleman, A. (2013). Insight in Schizophrenia: Involvement of Self-Reflection Networks? *Schizophrenia Bulletin*, *39*(6), 1288–1295. doi: 10.1093/schbul/sbs122

Van Der Meer, Lisette, Swart, M., Van Der Velde, J., Pijnenborg, G., Wiersma, D., Bruggeman, R., & Aleman, A. (2014). Neural Correlates of Emotion Regulation in Patients with Schizophrenia and Non-Affected Siblings. *PLoS ONE*, *9*(6), e99667. doi: 10.1371/journal.pone.0099667

Van Kleef, R. S., Marsman, J.-B. C., Van Valen, E., Bockting, C. L. H., Aleman, A., & Van Tol, M.-J. (2022). Neural basis of positive and negative emotion regulation in remitted depression. *NeuroImage: Clinical*, *34*, 102988. doi: 10.1016/j.nicl.2022.102988

Van Wingen, G. A., Van Eijndhoven, P., Tendolkar, I., Buitelaar, J., Verkes, R. J., & Fernández, G. (2011). Neural basis of emotion recognition deficits in first-episode major depression. *Psychological Medicine*, *41*(7), 1397–1405. doi: 10.1017/S0033291710002084

Vanes, L. D., Mouchlianitis, E., Collier, T., Averbeck, B. B., & Shergill, S. S. (2018). Differential neural reward mechanisms in treatment-responsive and treatment-resistant schizophrenia. *Psychological Medicine*, *48*(14), 2418–2427. doi: 10.1017/S0033291718000041

Vercammen, A., Morris, R., Green, M., Lenroot, R., Kulkarni, J., Carr, V., … Weickert, T. (2012). Reduced neural activity of the prefrontal cognitive control circuitry during response inhibition to negative words in people with schizophrenia. *Journal of Psychiatry & Neuroscience*, *37*(6), 379–388. doi: 10.1503/jpn.110088

Victor, T. A., Furey, M. L., Fromm, S. J., Öhman, A., & Drevets, W. C. (2010). Relationship Between Amygdala Responses to Masked Faces and Mood State and Treatment in Major Depressive Disorder. *Archives of General Psychiatry*, *67*(11), 1128. doi: 10.1001/archgenpsychiatry.2010.144

Vistoli, D., Lavoie, M.-A., Sutliff, S., Jackson, P. L., & Achim, A. M. (2017). Functional MRI examination of empathy for pain in people with schizophrenia reveals abnormal activation related to cognitive perspective-taking but typical activation linked to affective sharing. *Journal of Psychiatry and Neuroscience*, *42*(4), 262–272. doi: 10.1503/jpn.160136

Vizueta, N., Rudie, J. D., Townsend, J. D., Torrisi, S., Moody, T. D., Bookheimer, S. Y., & Altshuler, L. L. (2012). Regional fMRI Hypoactivation and Altered Functional Connectivity During Emotion Processing in Nonmedicated Depressed Patients With Bipolar II Disorder. *American Journal of Psychiatry*, *169*(8), 831–840. doi: 10.1176/appi.ajp.2012.11030349

Wagner, G., Koch, K., Schachtzabel, C., Sobanski, T., Reichenbach, J. R., Sauer, H., & Schlösser, R. G. M. (2010). Differential effects of serotonergic and noradrenergic antidepressants on brain activity during a cognitive control task and neurofunctional prediction of treatment outcome in patients with depression. *Journal of Psychiatry and Neuroscience*, *35*(4), 247–257. doi: 10.1503/jpn.090081

Wagner, G., Schachtzabel, C., Peikert, G., & Bär, K. (2015). The neural basis of the abnormal self‐referential processing and its impact on cognitive control in depressed patients. *Human Brain Mapping*, *36*(7), 2781–2794. doi: 10.1002/hbm.22807

Walsh, N. D., Williams, S. C. R., Brammer, M. J., Bullmore, E. T., Kim, J., Suckling, J., … Fu, C. H. Y. (2007). A Longitudinal Functional Magnetic Resonance Imaging Study of Verbal Working Memory in Depression After Antidepressant Therapy. *Biological Psychiatry*, *62*(11), 1236–1243. doi: 10.1016/j.biopsych.2006.12.022

Walter, H., Ciaramidaro, A., Adenzato, M., Vasic, N., Ardito, R. B., Erk, S., & Bara, B. G. (2009). Dysfunction of the social brain in schizophrenia is modulated by intention type: An fMRI study. *Social Cognitive and Affective Neuroscience*, *4*(2), 166–176. doi: 10.1093/scan/nsn047

Walter, H., Kammerer, H., Frasch, K., Spitzer, M., & Abler, B. (2009). Altered reward functions in patients on atypical antipsychotic medication in line with the revised dopamine hypothesis of schizophrenia. *Psychopharmacology*, *206*(1), 121–132. doi: 10.1007/s00213-009-1586-4

Walter, H., Vasic, N., Höse, A., Spitzer, M., & Wolf, R. C. (2007). Working memory dysfunction in schizophrenia compared to healthy controls and patients with depression: Evidence from event-related fMRI. *NeuroImage*, *35*(4), 1551–1561. doi: 10.1016/j.neuroimage.2007.01.041

Walter, H., Wolf, R. C., Spitzer, M., & Vasic, N. (2007). Increased left prefrontal activation in patients with unipolar depression: An event-related, parametric, performance-controlled fMRI study. *Journal of Affective Disorders*, *101*(1–3), 175–185. doi: 10.1016/j.jad.2006.11.017

Waltz, J. A., Schweitzer, J. B., Gold, J. M., Kurup, P. K., Ross, T. J., Jo Salmeron, B., … Stein, E. A. (2009). Patients with Schizophrenia have a Reduced Neural Response to Both Unpredictable and Predictable Primary Reinforcers. *Neuropsychopharmacology*, *34*(6), 1567–1577. doi: 10.1038/npp.2008.214

Wang, L., LaBar, K. S., Smoski, M., Rosenthal, M. Z., Dolcos, F., Lynch, T. R., … McCarthy, G. (2008). Prefrontal mechanisms for executive control over emotional distraction are altered in major depression. *Psychiatry Research: Neuroimaging*, *163*(2), 143–155. doi: 10.1016/j.pscychresns.2007.10.004

Wang, X., Li, P., Zheng, L., Liu, Z., Cui, G., Li, L., … Si, Y. (2022). The passive recipient: Neural correlates of negative self‐view in depression. *Brain and Behavior*, *12*(2). doi: 10.1002/brb3.2477

Wang, Y., Xu, C., Cao, X., Gao, Q., Li, J., Liu, Z., … Zhang, K. (2012). Effects of an antidepressant on neural correlates of emotional processing in patients with major depression. *Neuroscience Letters*, *527*(1), 55–59. doi: 10.1016/j.neulet.2012.08.034

Weathers, J., Brotman, M. A., Deveney, C. M., Kim, P., Zarate, C., Fromm, S., … Leibenluft, E. (2013). A developmental study on the neural circuitry mediating response flexibility in bipolar disorder. *Psychiatry Research: Neuroimaging*, *214*(1), 56–65. doi: 10.1016/j.pscychresns.2013.05.002

Weiss, A. P., Ellis, C. B., Roffman, J. L., Stufflebeam, S., Hamalainen, M. S., Duff, M., … Schacter, D. L. (2009). Aberrant Frontoparietal Function during Recognition Memory in Schizophrenia: A Multimodal Neuroimaging Investigation. *The Journal of Neuroscience*, *29*(36), 11347–11359. doi: 10.1523/JNEUROSCI.0617-09.2009

Weiss, A. P., Goff, D., Schacter, D. L., Ditman, T., Freudenreich, O., Henderson, D., & Heckers, S. (2006). Fronto-Hippocampal Function During Temporal Context Monitoring in Schizophrenia. *Biological Psychiatry*, *60*(11), 1268–1277. doi: 10.1016/j.biopsych.2006.06.025

Weiss, E. M., Golaszewski, S., Mottaghy, F. M., Hofer, A., Hausmann, A., Kemmler, G., … Wolfgang Fleischhacker, W. (2003). Brain activation patterns during a selective attention test—A functional MRI study in healthy volunteers and patients with schizophrenia. *Psychiatry Research: Neuroimaging*, *123*(1), 1–15. doi: 10.1016/S0925-4927(03)00019-2

Weiss, E. M., Siedentopf, C., Golaszewski, S., Mottaghy, F. M., Hofer, A., Kremser, C., … Fleischhacker, W. W. (2007). Brain activation patterns during a selective attention test—A functional MRI study in healthy volunteers and unmedicated patients during an acute episode of schizophrenia. *Psychiatry Research: Neuroimaging*, *154*(1), 31–40. doi: 10.1016/j.pscychresns.2006.04.009

Wende, K. C., Nagels, A., Stratmann, M., Chatterjee, A., Kircher, T., & Straube, B. (2015). Neural basis of altered physical and social causality judgements in schizophrenia. *Schizophrenia Research*, *161*(2–3), 244–251. doi: 10.1016/j.schres.2014.11.007

Werner, N. S., Meindl, T., Materne, J., Engel, R. R., Huber, D., Riedel, M., … Hennig-Fast, K. (2009). Functional MRI study of memory-related brain regions in patients with depressive disorder. *Journal of Affective Disorders*, *119*(1–3), 124–131. doi: 10.1016/j.jad.2009.03.003

Wessa, M., Houenou, J., Paillère-Martinot, M.-L., Berthoz, S., Artiges, E., Leboyer, M., & Martinot, J.-L. (2007). Fronto-Striatal Overactivation in Euthymic Bipolar Patients During an Emotional Go/NoGo Task. *American Journal of Psychiatry*, *164*(4), 638–646. doi: 10.1176/ajp.2007.164.4.638

Whalley, H. C., McKirdy, J., Romaniuk, L., Sussmann, J., Johnstone, E. C., Wan, H. I., … Hall, J. (2009). Functional imaging of emotional memory in bipolar disorder and schizophrenia. *Bipolar Disorders*, *11*(8), 840–856. doi: 10.1111/j.1399-5618.2009.00768.x

Whalley, M. G., Rugg, M. D., & Brewin, C. R. (2012). Autobiographical memory in depression: An fMRI study. *Psychiatry Research: Neuroimaging*, *201*(2), 98–106. doi: 10.1016/j.pscychresns.2011.08.008

White, D. M., Kraguljac, N. V., Reid, M. A., & Lahti, A. C. (2015). Contribution of substantia nigra glutamate to prediction error signals in schizophrenia: A combined magnetic resonance spectroscopy/functional imaging study. *Npj Schizophrenia*, *1*(1), 14001. doi: 10.1038/npjschz.2014.1

Williams, L. (Lea) M., Das, P., Liddell, B. J., Olivieri, G., Peduto, A. S., David, A. S., … Harris, A. W. F. (2007). Fronto-limbic and autonomic disjunctions to negative emotion distinguish schizophrenia subtypes. *Psychiatry Research: Neuroimaging*, *155*(1), 29–44. doi: 10.1016/j.pscychresns.2006.12.018

Willinger, D., Karipidis, I. I., Neuer, S., Emery, S., Rauch, C., Häberling, I., … Brem, S. (2022). Maladaptive Avoidance Learning in the Orbitofrontal Cortex in Adolescents With Major Depression. *Biological Psychiatry: Cognitive Neuroscience and Neuroimaging*, *7*(3), 293–301. doi: 10.1016/j.bpsc.2021.06.005

Wilmsmeier, A., Ohrmann, P., Suslow, T., Siegmund, A., Koelkebeck, K., Rothermundt, M., … Pedersen, A. (2010). Neural correlates of set-shifting: Decomposing executive functions in schizophrenia. *Journal of Psychiatry and Neuroscience*, *35*(5), 321–329. doi: 10.1503/jpn.090181

Wojtalik, J. A., & Barch, D. M. (2014). An fMRI Study of the Influence of a History of Substance Abuse on Working Memory-Related Brain Activation in Schizophrenia. *Frontiers in Psychiatry*, *5*. doi: 10.3389/fpsyt.2014.00001

Wolf, C., Linden, S., Jackson, M. C., Healy, D., Baird, A., Linden, D. E. J., & Thome, J. (2011). Brain Activity Supporting Working Memory Accuracy in Patients with Paranoid Schizophrenia: A Functional Magnetic Resonance Imaging Study. *Neuropsychobiology*, *64*(2), 93–101. doi: 10.1159/000323800

Wolf, D. H. (2011). Striatal intrinsic reinforcement signals during recognition memory: Relationship to response bias and dysregulation in schizophrenia. *Frontiers in Behavioral Neuroscience*, *5*. doi: 10.3389/fnbeh.2011.00081

Woodward, N. D., Waldie, B., Rogers, B., Tibbo, P., Seres, P., & Purdon, S. E. (2009). Abnormal prefrontal cortical activity and connectivity during response selection in first episode psychosis, chronic schizophrenia, and unaffected siblings of individuals with schizophrenia. *Schizophrenia Research*, *109*(1–3), 182–190. doi: 10.1016/j.schres.2008.11.028

Xiao, Q., Wu, Z., Hui, X., Jiao, Q., Zhong, Y., Su, L., & Lu, G. (2021). Manic and euthymic states in pediatric bipolar disorder patients during an emotional Go/Nogo task: A functional magnetic resonance imaging study. *Journal of Affective Disorders*, *282*, 82–90. doi: 10.1016/j.jad.2020.12.105

Yang, J.-C. (2004). Functional Neuroanatomy in Depressed Patients with Sexual Dysfunction: Blood Oxygenation Level Dependent Functional MR Imaging. *Korean Journal of Radiology*, *5*(2), 87. doi: 10.3348/kjr.2004.5.2.87

Yang, T. T., Simmons, A. N., Matthews, S. C., Tapert, S. F., Frank, G. K., Bischoff-Grethe, A., … Paulus, M. P. (2009). Depressed adolescents demonstrate greater subgenual anterior cingulate activity. *NeuroReport*, *20*(4), 440–444. doi: 10.1097/WNR.0b013e3283262e10

Yang, X., Huang, J., Lan, Y., Zhu, C., Liu, X., Wang, Y., … Chan, R. C. K. (2016). Diminished caudate and superior temporal gyrus responses to effort-based decision making in patients with first-episode major depressive disorder. *Progress in Neuro-Psychopharmacology and Biological Psychiatry*, *64*, 52–59. doi: 10.1016/j.pnpbp.2015.07.006

Yoo, S.-S., Choi, B.-G., Juh, R.-H., Park, J.-M., Pae, C.-U., Kim, J.-J., … Adkinson, N. F. (2005). Working memory processing of facial images in schizophrenia: fMRI investigation. *International Journal of Neuroscience*, *115*(3), 351–366. doi: 10.1080/00207450590520957

Yoon, J. H., Minzenberg, M. J., Raouf, S., D’Esposito, M., & Carter, C. S. (2013). Impaired Prefrontal-Basal Ganglia Functional Connectivity and Substantia Nigra Hyperactivity in Schizophrenia. *Biological Psychiatry*, *74*(2), 122–129. doi: 10.1016/j.biopsych.2012.11.018

Yoon, L., Rohrsetzer, F., Battel, L., Anés, M., Manfro, P. H., Rohde, L. A., … Swartz, J. R. (2022). Reward‐ and threat‐related neural function associated with risk and presence of depression in adolescents: A study using a composite risk score in Brazil. *Journal of Child Psychology and Psychiatry*, *63*(5), 579–590. doi: 10.1111/jcpp.13496

Young, K. D., Erickson, K., Nugent, A. C., Fromm, S. J., Mallinger, A. G., Furey, M. L., & Drevets, W. C. (2012). Functional anatomy of autobiographical memory recall deficits in depression. *Psychological Medicine*, *42*(2), 345–357. doi: 10.1017/S0033291711001371

Young, Kymberly D, Bodurka, J., & Drevets, W. C. (2016). Differential neural correlates of autobiographical memory recall in bipolar and unipolar depression. *Bipolar Disorders*, *18*(7), 571–582. doi: 10.1111/bdi.12441

Yüksel, D., Dietsche, B., Konrad, C., Dannlowski, U., Kircher, T., & Krug, A. (2018). Neural correlates of working memory in first episode and recurrent depression: An fMRI study. *Progress in Neuro-Psychopharmacology and Biological Psychiatry*, *84*, 39–49. doi: 10.1016/j.pnpbp.2018.02.003

Zarp Petersen, J., Varo, C., Skovsen, C. F., Ott, C. V., Kjærstad, H. L., Vieta, E., … Miskowiak, K. W. (2022). Neuronal underpinnings of cognitive impairment in bipolar disorder: A large data‐driven functional magnetic resonance imaging study. *Bipolar Disorders*, *24*(1), 69–81. doi: 10.1111/bdi.13100

Zedkova, L., Woodward, N. D., Harding, I., Tibbo, P. G., & Purdon, S. E. (2006). Procedural learning in schizophrenia investigated with functional magnetic resonance imaging. *Schizophrenia Research*, *88*(1–3), 198–207. doi: 10.1016/j.schres.2006.06.039

Zhang, Z., Shi, J., Yuan, Y., Hao, G., Yao, Z., & Chen, N. (2008). Relationship of auditory verbal hallucinations with cerebral asymmetry in patients with schizophrenia: An event-related fMRI study. *Journal of Psychiatric Research*, *42*(6), 477–486. doi: 10.1016/j.jpsychires.2007.04.003

Zheng, Y., Wu, C., Li, J., Wu, H., She, S., Liu, S., … Li, L. (2016). Brain substrates of perceived spatial separation between speech sources under simulated reverberant listening conditions in schizophrenia. *Psychological Medicine*, *46*(3), 477–491. doi: 10.1017/S0033291715001828

Zhong, M., Wang, X., Xiao, J., Yi, J., Zhu, X., Liao, J., … Yao, S. (2011). Amygdala hyperactivation and prefrontal hypoactivation in subjects with cognitive vulnerability to depression. *Biological Psychology*, *88*(2–3), 233–242. doi: 10.1016/j.biopsycho.2011.08.007

Zierhut, K., Bogerts, B., Schott, B., Fenker, D., Walter, M., Albrecht, D., … Schiltz, K. (2010). The role of hippocampus dysfunction in deficient memory encoding and positive symptoms in schizophrenia. *Psychiatry Research: Neuroimaging*, *183*(3), 187–194. doi: 10.1016/j.pscychresns.2010.03.007

Zweerings, J., Zvyagintsev, M., Turetsky, B. I., Klasen, M., König, A. A., Roecher, E., … Mathiak, K. (2019). Fronto‐parietal and temporal brain dysfunction in depression: A fMRI investigation of auditory mismatch processing. *Human Brain Mapping*, hbm.24623. doi: 10.1002/hbm.24623
